# Mitigating Intensive Care Unit Noise: Design-Led Modeling Solutions, Calculated Acoustic Outcomes, and Cost Implications

**DOI:** 10.1177/19375867241237501

**Published:** 2024-03-21

**Authors:** Emil E. Jonescu, Benjamin Farrel, Chamil Erik Ramanayaka, Christopher White, Giuseppe Costanzo, Lori Delaney, Rebecca Hahn, Janet Ferrier, Edward Litton

**Affiliations:** 1 Hames Sharley, Perth, Western Australia, Australia; 2 School of Arts and Humanities, Edith Cowan University, Perth, Western Australia, Australia; 3 Gabriels Hearn Farrell Pty Ltd, South Perth, Western Australia, Australia; 4 Central Queensland University, School of Engineering and Technology, Brisbane, Queensland, Australia; 5 Sage Quantity Surveyors, Perth, Western Australia, Australia; 6 School of Nursing, Midwifery and Social Work, University of Queensland, St Lucia, Brisbane, Queensland, Australia; 7 College of Medicine and Health Sciences, Australian National University, Acton, Canberra, Australia; 8 Heart and Lung Research Institute of WA, Harry Perkins Institute of Medical Research, Murdoch, Western Australia, Australia; 9 School of Health and Medical Science, Surgery, University of Western Australia, Crawley, Western Australia, Australia; 10 Cardiothoracic and Transplant Surgery Department, Fiona Stanley Hospital, Murdoch, Western Australia, Australia; 11 Intensive Care Unit, St. John of God Hospital, Subiaco, Western Australia, Australia; 12 ANZSCTS National Cardiac Surgery Data Base, St John of God Hospital, Perth Western Australia; 13 Intensive Care Unit, Fiona Stanley Hospital, Murdoch, Western Australia, Australia; 14 School of Medicine, University of Western Australia, Crawley, Western Australia, Australia

**Keywords:** intensive care unit (ICU), sleep, noise, acoustic, design, hospital, healthcare

## Abstract

**Objectives, Purpose, or Aim:**

The study aimed to decrease noise levels in the ICU, anticipated to have adverse effects on both patients and staff, by implementing enhancements in acoustic design.

**Background:**

Recognizing ICU noise as a significant disruptor of sleep and a potential hindrance to patient recovery, this study was conducted at a 40-bed ICU in Fiona Stanley Hospital in Perth, Australia.

**Methods:**

A comprehensive mixed-methods approach was employed, encompassing surveys, site analysis, and acoustic measurements. Survey data highlighted the importance of patient sleep quality, emphasizing the negative impact of noise on work performance, patient connection, and job satisfaction. Room acoustics analysis revealed noise levels ranging from 60 to 90 dB(A) in the presence of patients, surpassing sleep disruption criteria.

**Results:**

Utilizing an iterative 3D design modeling process, the study simulated significant acoustic treatment upgrades. The design integrated effective acoustic treatments within patient rooms, aiming to reduce noise levels and minimize transmission to adjacent areas. Rigorous evaluation using industry-standard acoustic software highlights the design’s efficacy in reducing noise transmission in particular. Additionally, cost implications were examined, comparing standard ICU construction with acoustically treated options for new construction and refurbishment projects.

**Conclusions:**

This study provides valuable insights into design-based solutions for addressing noise-related challenges in the ICU. While the focus is on improving the acoustic environment by reducing noise levels and minimizing transmission to adjacent areas. It is important to clarify that direct measurements of patient outcomes were not conducted. The potential impact of these solutions on health outcomes, particularly sleep quality, remains a crucial aspect for consideration.

## Introduction and Background

Existing literature suggests that Intensive Care Unit (ICU) design and inadequate acoustic control contribute to sleep deprivation, which has negative impacts on health outcomes. Up to 50% of ICU patients experience sleep disturbance, with noise being a primary factor (Beltrami et al., 2015). Sleep is a crucial biological function, with humans typically needing 7–9 hr of sleep per night. It involves two main sleep phases, non-rapid eye movement (REM) sleep and REM sleep, occurring in 90-min cycles (Daou et al., 2020). Sleep deprivation and persistent sleep loss have significant consequences for cognitive, physical, metabolic, and immunological functions (Nilius et al., 2021).

Critical illnesses requiring ICU admission disrupt normal sleep architecture and circadian rhythm. ICU patients often experience atypical sleep architecture characterized by longer sleep latency, frequent arousals, increased wakefulness, and reduced slow wave and REM sleep. Sleep quality in ICU patients is generally poor, predominantly consisting of light sleep stages susceptible to noise disturbance. As a result, ICU patients sleep during both day and night, with around 50% of their sleep occurring during the day (Broussard et al., 2012; Tembo, Parker, & Higgins, 2013). A study by Cooper et al. (2000) using polysomnography on mechanically ventilated ICU patients revealed abnormal sleep patterns in all patients (Elliott et al., 2013).

ICU sleep disruption can lead to short-term and long-term adverse effects. Observational studies have shown an association between ICU sleep deprivation and the development of delirium (Johansson et al., 2018), with delirium incidence in the ICU reported as high as 87%, leading to increased cost, longer stays, and higher mortality rates (Busch-Vishniac & Ryherd, 2023; Reade & Finfer, 2014; Weinhouse et al., 2009). Sleep disturbance in the ICU can persist in survivors of critical illness and is independently associated with poor psychological recovery after ICU admission (Helton et al., 1980; Little et al., 2012; Orr & Stahl, 1977). Persistent sleep disturbance following critical illness is also linked to long-term reduced health-related quality of life (McKinley et al., 2012; Orwelius et al., 2008).

Patients in ICUs consistently report that environmental factors, including noise, significantly contribute to sleep disruption (Delaney et al., 2017). Noise levels within the ICU typically range between 50 and 65 dB(A), with peak levels reaching 70–85 dB(A) (Delaney et al., 2017; Elliott et al., 2013), and show limited variation throughout the day. Tailored interventions targeting behavior and the clinical environment have been suggested to reduce the primary sources of noise. Prolonged exposure to ICU noise levels can lead to undesirable physiological effects, such as increased cortisol levels, heightened catabolism, and oxygen consumption (Trivedi et al., 2017: Moriyama et al., 1999). Recommendations have been made to modify the physical environment, such as interventions using alarm management guidelines that recommended “personalizing” alarm parameters and a training package that delivered an online module and an experiential simulation session, giving staff members a “patient experience” of the ICU (Darbyshire et al., 2019, 1022–1023), and sound-absorbing materials on walls and ceiling tiles (Litton Personal Communication, 2023), with earplug studies demonstrating a reduction in perceived noise by approximately 10 dB (Litton et al., 2017). Considering the challenges in the effectiveness of pharmacological interventions for managing noise-related sleep disturbances, a design-oriented approach becomes even more crucial (Wibrow et al., 2022), particularly when addressing sleep disturbance prevention, which may include various pharmacological options for managing delirium.

Design solutions are recognized as one component of a broader multifaceted intervention to improve sleep, aligning with ICU guidelines (Johansson et al., 2018; Litton et al., 2021). Research indicates that sleep deprivation in ICUs and other hospital wards is primarily caused by short, sharp, intermittent noise events, including medical alarms, staff communication, and vocalization from other patients rather than constant noise sources like air-conditioning and ventilation systems. Additionally, while not specific to health environments, other studies emphasize the importance of understanding sleep disturbance criteria and controlling noise’s impact on sleep. Research on the impact of traffic noise on sleep disturbance suggests that noise levels below Lmax 50–55 dB(A) are unlikely to awaken individuals from sleep. Additionally, one or two noise events per night at a level of Lmax 65–70 dB(A) are not expected to significantly affect health and well-being (New South Wales Government Roads and Maritime Services, 2015). However, it is important to consider contextual differences and the vulnerability of those affected.^1^

This ICU research partnership further extends the work of Johansson et al. (2018), specifically relating to critical areas of (1) conducting cost analysis, (2) fostering active collaboration with hospital and medical personnel throughout the project’s lifecycle, and (3) engaging a broader team of researchers in data collection to ensure rigorous protocol adherence while alleviating the additional workload on medical and nursing staff. Accordingly, progressing the scholarly pursuit of enhancing the healthcare environment’s acoustic quality, this study involves a multidisciplinary collaboration between intensive care specialists, healthcare professionals, architects, acoustic consultants, and quantity surveyors as a community-of-practice.

This study seeks to comprehensively investigate the impact of noise in ICU environments on patient sleep quality and clinician performance. To achieve this, the research methodology combines site visits, field studies, and 3D acoustic modeling to assess and mitigate excessive noise levels in ICUs. By providing a detailed analysis of noise and its effects on patients and clinicians, the study aims to offer practical recommendations for design- and specification-based approaches to noise mitigation. Furthermore, the study explores the economic implications of implementing noise reduction measures, including the development of an Opinion of Probable Cost (OPC). Ultimately, the research endeavors to support clinicians in providing care through design by contributing to the collective body of knowledge in ICU patient sleep-related research. In doing so, the research aims to influence the adaptation of existing and future ICU designs, aligning with healthcare design guidelines.

## Method

This Fiona Stanley Hospital Quality Improvement Activity (Approval No. 46840) utilized a mixed-methods design, including surveys, case study analysis, fieldwork observations, and environmental data collection. The design-led approach seeks to mitigate ICU noise as follows: Firstly, site visits were conducted to analyze the baseline level, frequency, characteristics, and sources of noise in the ICU, considering factors such as finishes, fixtures, equipment, and spatial layout. Acoustic calculations were conducted using the EASE 4.4 software with the Aura module. These baseline levels were compared against established guidelines, including the World Health Organization (WHO) Guidelines for Community Noise (1999) which recommend Leq 30 dB(A) and Lmax(F) 40 dB(A) at night, and AS/NZS 2017:2016 Acoustics—Recommended design sound levels and reverberation times (RTs) for building interiors of Leq 40–45 dB(A) for steady-state and quasi-steady-state noise sources like air-conditioning and traffic noise intrusion.^2^ Secondly, a field study involved placing acoustic data loggers in a single-occupancy ICU room at Fiona Stanley Hospital over several weeks in 2022.

Subsequently, comprehensive 3D acoustic modeling and specifications were developed to mitigate excessive noise while adhering to the Australasian Health Facility Guidelines for single-occupancy ICU rooms. The 3D model included the adjoining suite, considering that the sliding door between them is often open. These guidelines, which integrate ongoing research and clinical input, served as valuable intellectual property for the project. Thirdly, surveys at Fiona Stanley Hospital sought to understand the impact of noise on clinician performance, providing valuable clinical insights for the development of a new acoustic design model for a single-occupancy ICU room and advancing our understanding by building upon previous findings (Schmidt et al., 2020). Lastly, the 3D model was quantified, and their possible impacts were understood. Three models and accompanying specifications were developed and assessed using acoustic simulation software to simulate the projected acoustic performance and make further recommendations for improvements and optimal spatial setup considering cost-effectiveness.

**Figure 1.**
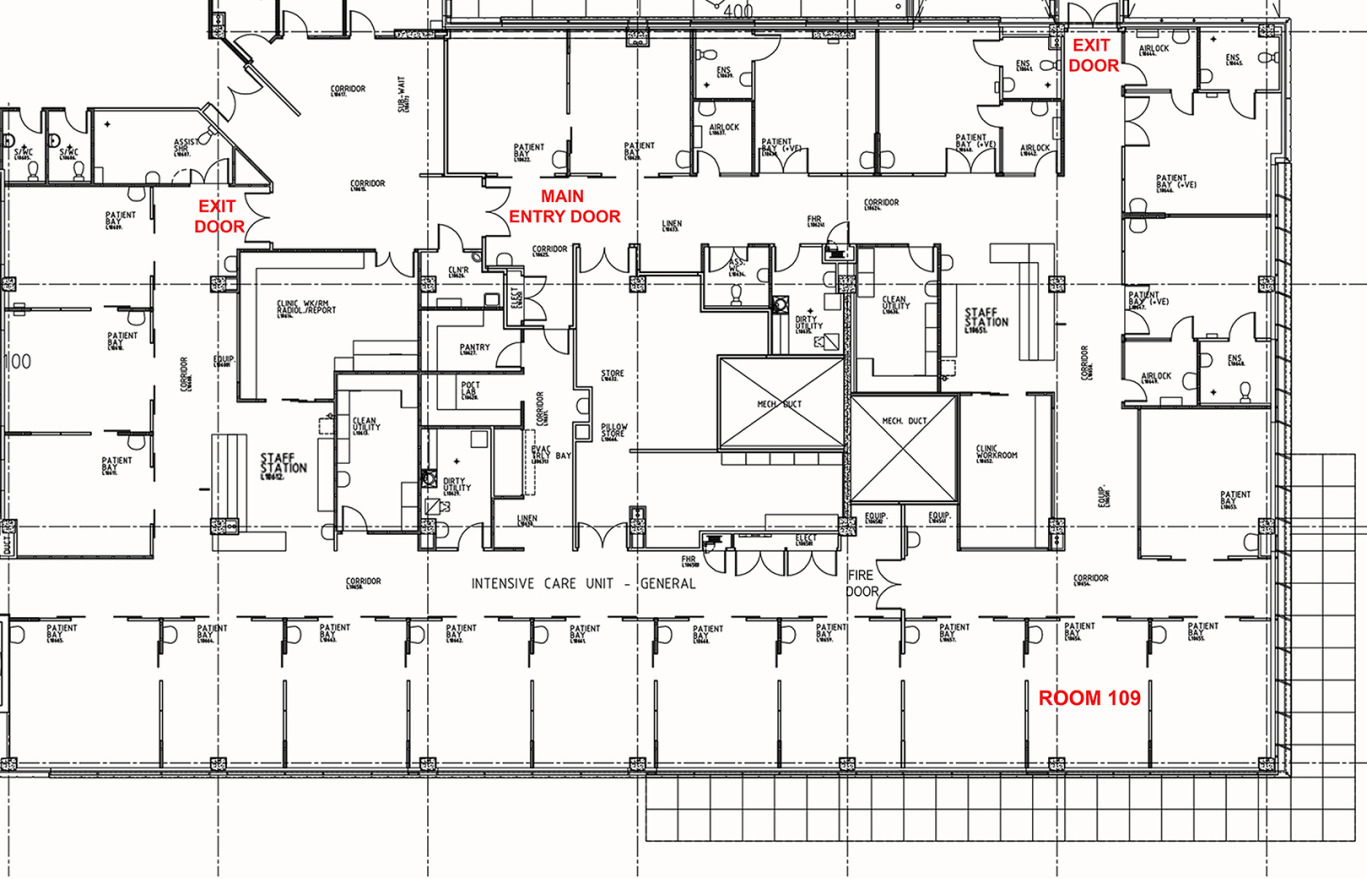
Room 109 within typical ICU layout at Fiona Stanley Hospital.

**Figure 2.**
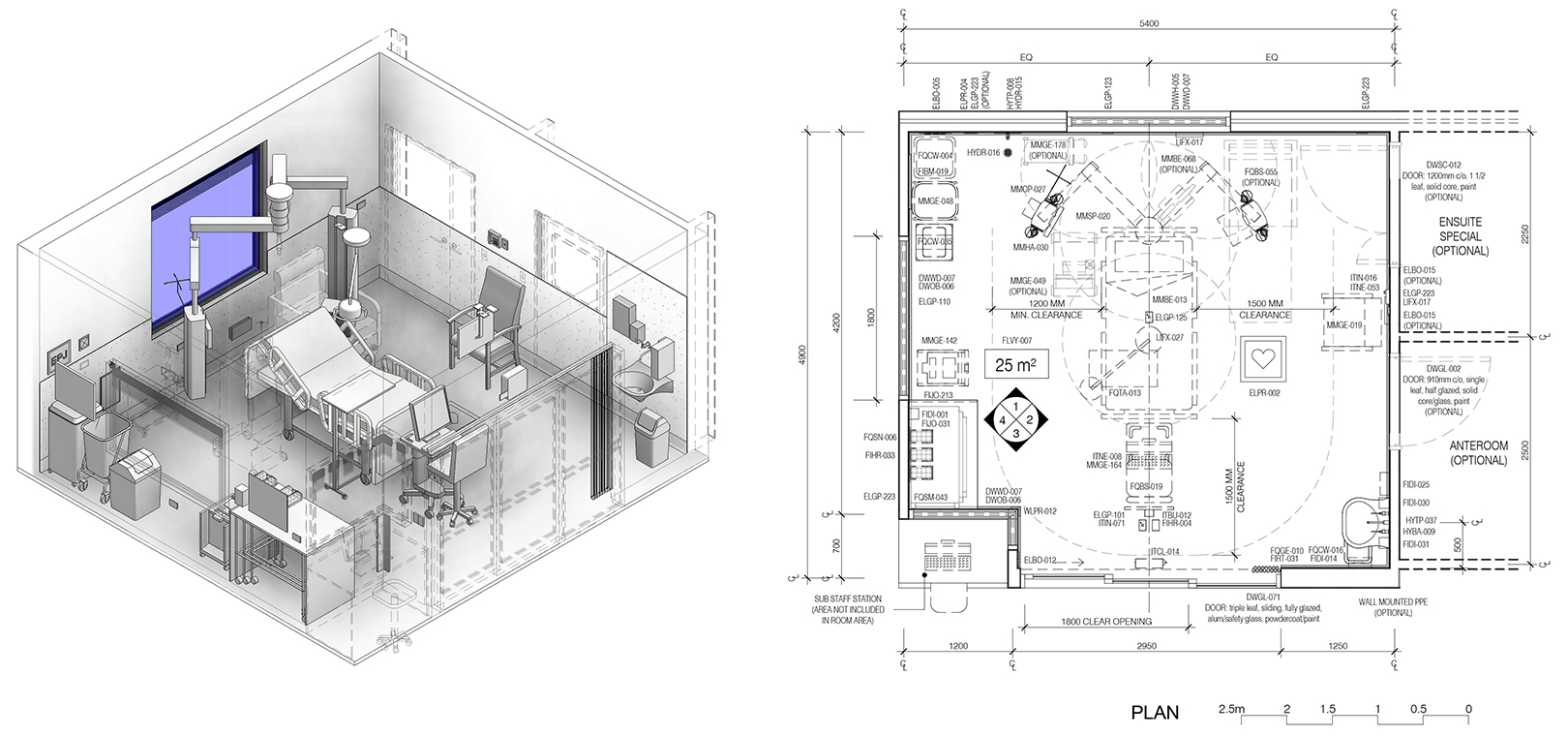
Typical layout of a one-bedroom—intensive Care room (Australasian Health Infrastructure Alliance, 2019).

The case study (Room 109) is a single-occupancy ICU room at Fiona Stanley Hospital with a floor area of 25 m^2^ and operates 24 hr a day and is intended for one patient requiring intensive medical treatment, one visitor, and six to eight staff members. Site visits were conducted (1) to assess finishes, fixtures, equipment, and spatial layout against the Australasian Health Facility Guidelines (Australasian Health Infrastructure Alliance, 2019) and (2) to examine the sound characteristics of bedside machines establishing a baseline audio profile and to commence sound logging^3^ from 10 a.m. Tuesday, August 16 to 7:00 a.m. Tuesday, August 23, 2022. Wall surfaces totaling 61.8 m^2^, consisting of plasterboard and glazing, are typical of a one-bedroom ICU room (Figures 1 and 2; Australasian Health Infrastructure Alliance, 2019).^4^ The ceiling is an Armstrong Bioguard, Noise Reduction Coefficient (NRC): 0.7, mineral fiber suspended tile with a plenum depth of approximately 1.0 m, deviating from the typical acoustically reflective flush plasterboard ceilings found in most ICU wards in Western Australia. The room’s floor surface is seamless vinyl with coved vinyl skirting.^5,6^ A calibrated Class 1 sound-level meter, the Cirrus Optimus CR199B (Serial number G061705), was used for noise monitoring. It complies with National Association of Testing Authorities (NATA) standards and government requirements for acoustic calibration and stores noise level data in the A-weighted scale (dB[A]). The equipment was calibrated before and after the monitoring process.

**Figure 3.**
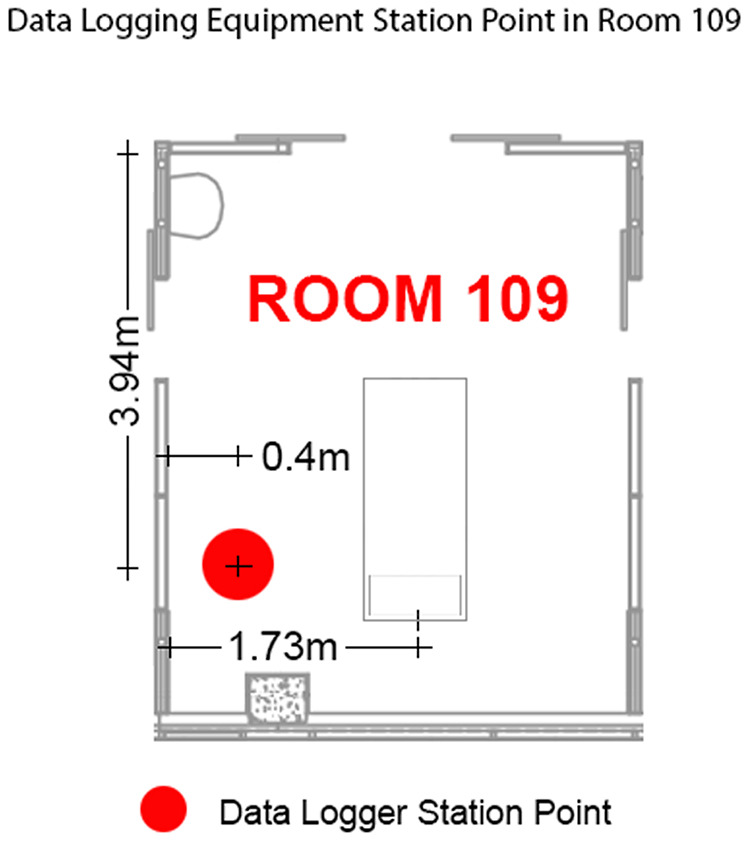
Positioning of noise data logging equipment between 10 a.m. Tuesday August 16 and 7:00 a.m. Tuesday August 23, 2022.

The placement of the noise monitor and data logger was determined in consultation with the Nurse Manager to ensure it did not disrupt normal operations (Figure 3). Adjacent to the noise monitor was a glazed sliding door onto the adjoining single-occupancy ICU room. The sliding door is commonly left open, given that clinical staff are tasked with observing the two adjoining rooms.

The data logger captured readings at 1-min intervals throughout the monitoring period. Postprocessing was performed using proprietary software to obtain noise level data, time profiles, and statistical noise levels. The data were graphed at 15-min intervals in conjunction with the LAmax noise level, representing the highest recorded noise level (peak) within each interval. This analysis aimed to identify any consistent sound signatures related to equipment, alarms, or loud conversations within the room. LAeq and LA90 noise levels were analyzed in 15-min intervals to assess average and background noise levels in the room. As a separate exercise, equipment alarms were measured in an unoccupied ICU room using a handheld sound-level meter to assess their impact on maximum in-room noise levels. This exploration aimed to identify potential noise reduction strategies by adjusting alarm volumes. The noise levels were measured using a NATA calibrated Bruel and Kjaer 2270 Hand-held analyzer (Serial No. 2644641), a Class 1 sound-level meter capturing data at 1 m from the tested equipment. Detailed noise level data, including Leq and one-third octave frequency data, can be found in Online Appendix B with dominant frequencies highlighted. Table 1 highlights that the Hamilton S1 Ventilator alarm, Cisco Telephone, and Phillips MX-800 Patient Monitor alarm produce the loudest alarms/tones. In their study, Darbyshire et al. (2019) suggested that alarms on physiological monitors that default to standard volume settings on power-up could be adjusted by the nursing staff; however, in practice, this rarely occurred, and although it was possible to adjust the volume settings for ventilator and infusion pump alarms, and in their experience, these were not adjusted routinely. Thus, on-site trials would be necessary to ensure that if the alarm volumes were to be reduced that they remain sufficiently audible.

**Table 1.** Medical Equipment and Noise Generating Device Testing in Unoccupied ICU Room.

Equipment	Volume Setting	Leq Noise Levels (5 s)	Dominant One-Third Octave Frequency
Phillips MX-800 Patient Monitor 865240	Maximum volume (Setting 10) Medium volume (Setting 5) Low volume (Setting 2)	70.2 dB(A) 55.6 dB(A) 48.8 dB(A)	2.5 kHz and 500 Hz
Hamilton S1 Ventilator	Maximum volume (Setting 10) Medium volume (Setting 5)	73.5 dB(A) 55.2 dB(A)	800 Hz
Carefusion Alaris Infusion Pump 8015	Maximum volume (volume not adjustable)	67.7 dB(A)	2 kHz
Cisco Telephone CP-7841	Maximum ring volume Medium ring volume	76.1 dB(A) 66.4 dB(A)	2 kHz

**Figure 4.**
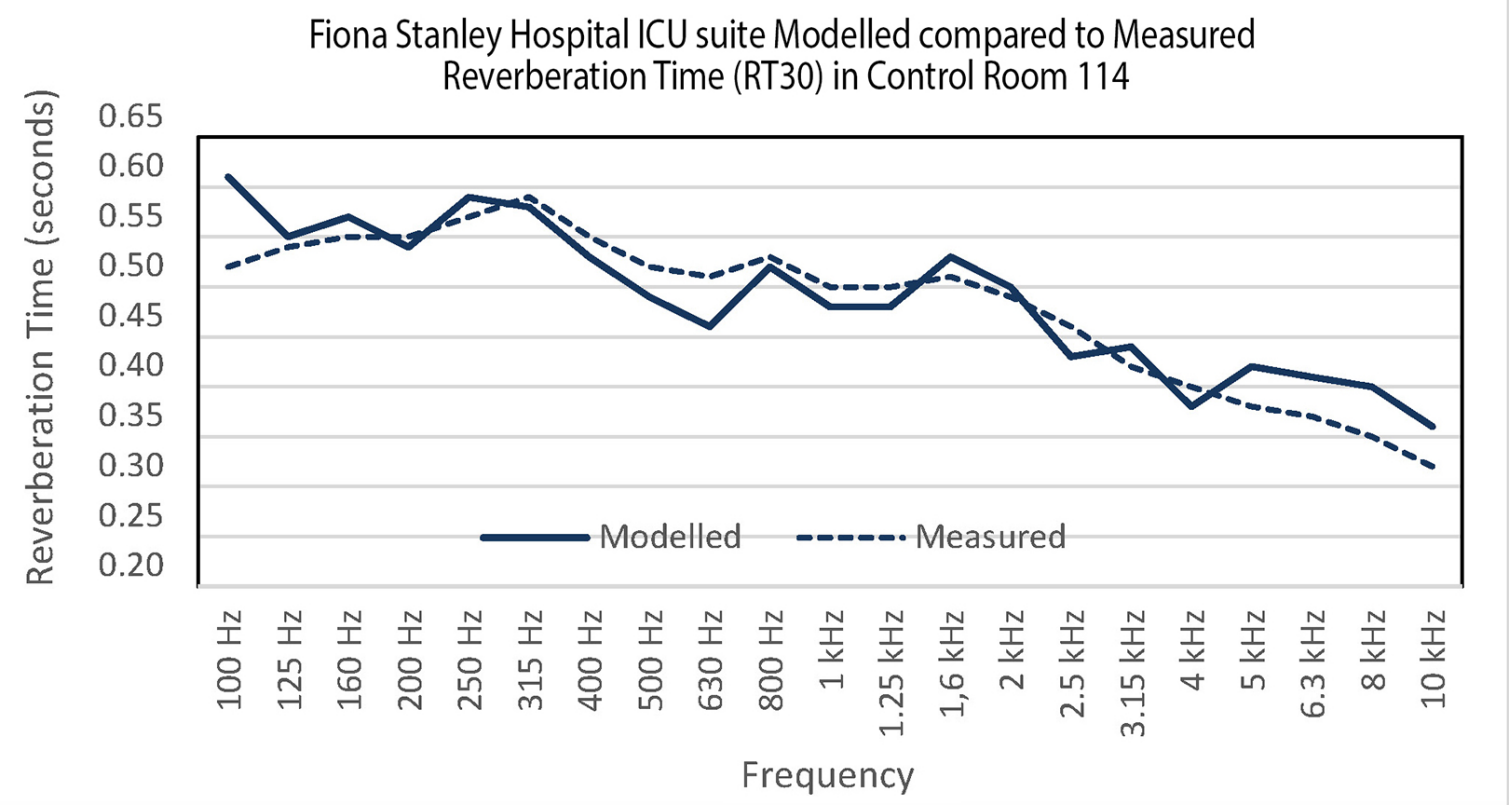
Comparison between the modeled and measured reverberation time data.

RT assessment was conducted in an identical room (Room 114) to maintain consistency. It is impractical to measure RT in occupied patient rooms due to the potential interference from monitoring equipment, patients, and staff noise. The accuracy of the base model, representing the existing ICU suites, was verified by comparing the calculated RT from the model with the on-site measurements taken during the project’s Practical Completion stage in December 2013. The calculated RT in the base model showed a strong correlation with the measured RT as depicted in Figure 4.

### Iterative Design

A design workshop involving ICU physicians was conducted to ensure that acoustic recommendations aligned with clinical requirements prior to the design process. A precise 3D digital model of the case study was recreated with REVIT software, supporting cross-disciplinary collaboration among different professionals. The digital model was employed to examine design iterations, including acoustic sound modeling that simulates various acoustical parameters of the space. This process encompasses deterministic image modeling, noise mapping, hybrid stochastic ray tracing, and cost estimation (Tiong et al., 2019).

### Survey Design for Fiona Stanley Hospital ICU Staff

The field study, which was a questionnaire survey, examined health professionals’ perceptions of noise impact on patient sleep and staff duties in the ICU. These perceptions focused on staff performance, well-being, and noise sources such as alarms and patient procedures. The study collaboratively developed this questionnaire with clinical and research experts in critical care, sleep, and healthcare design consultants.

The initial questions were informed by a literature review, and then the content of the questionnaire survey was first reviewed by an ICU nurse researcher and educator specializing in investigating sleep monitoring techniques and sleep disturbance among ICU patients. After addressing their feedback, the modified questionnaire content was reviewed by an Intensive care specialist, clinical professor/research fellow. Then, the questionnaire was reviewed by a clinical nurse Intensive Care Specialist, Clinical Professor/Research Fellow Clinical Nurse Specialist Research (ICU) for wording, layout and clarity, and adequacy of information. Upon revision, a statistician evaluated the questionnaire from a statistical perspective and provided feedback. After addressing the feedback, the final draft was sent to all pilot survey participants for confirmation. The pilot survey respondents did not participate in the actual online survey. The approved questionnaire is provided in Online Appendix E. Questions 1–5 focused on participant’s demographic data while the remaining 17 questions were the measures of noise impact on staff and patient’s well-being.

Participants in the deidentified online survey were provided written informed consent, ensuring disclosure of the study’s purpose, potential risks, and benefits. The survey link provided emphasized the voluntary nature of participation and assured confidentiality. By completing the survey, participants acknowledged their understanding of the information statement and voluntarily consented to take part in the project. The survey used Microsoft Forms ensuring anonymity and collected both qualitative and quantitative data through multiple-choice questions and freeform answers. Participants used a 1–7 Likert-type scale to rank their responses (1 = *strongly disagree*, 2 = *disagree*, 3 = *somewhat disagree*, 4 = *neither agree nor disagree*, 5 = *somewhat agree*, 6 = *agree*, and 7 = *strongly agree*), and they had the opportunity to provide comments.

The survey was emailed to ICU staff through the research office aligned with the ICU. The survey remained open for responses from 29/9/2022 to 9/11/2022. Approximately 200 eligible primary staff members (100 nurses, 50 doctors, and 50 allied health professionals and administrative staff) were targeted for participation. The inclusion criteria required a minimum employment duration of 3 months in the ICU. Exclusions encompassed individuals with limited English-language proficiency and non-ICU employees, such as visiting staff and clinical rotation students.

Data analysis was conducted using IBM SPSS Version 28 (IBM Corp, 2021). The demographic variables were analyzed using frequencies. As illustrated in Table 2, participant’s perceptions of ICU Acoustic Performance (ICUAP) were coded before analysis. Mean or median was used to evaluate sample statistics before the use of inferential statistics to test the following hypotheses (Hypothesis 1 [H1] and Hypothesis 2 [H2]): H2 was tested against the amount of experience as a result of the opinions provided by the pilot survey participants.

**H1:** The participants “strongly disagree/disagree/somewhat disagree/neither agree nor disagree/ somewhat agree/agree/strongly agree” toward ICUAP# (1–17).
**H2:** ICUAP# (1–17) is influenced by participants’ amount of ICU experience.

**Table 2.** ICU Acoustic Performance (ICUAP) IDs and Factors Descriptions Deriving From Survey Questionnaire

Question_ ID	Factor Description
ICUAP_01	The way in which the ICU is designed helps me to perform my duties.
ICUAP_02	The ICU allows me to communicate efficiently with other staff in the ICU.
ICUAP_03	I am able to communicate safely and effectively without disrupting patient sleep.
ICUAP_04	Noise levels in the ICU negatively impact on clinical care.
ICUAP_05	Noise levels in the ICU negatively impact staff well-being.
ICUAP_06	Impact of noise in the ICU is different to other areas such as trauma and emergency.
ICUAP_07	Alarms are a significant source of noise in the ICU.
ICUAP_08	Talking between clinicians is a significant source of noise in the ICU.
ICUAP_09	Patient procedures are a significant source of noise in the ICU.
ICUAP_10	Managing ventilation and life-support equipment is a significant source of noise in the ICU.
ICUAP_11	Visitors such as family members and/or ancillary staff are a significant source of noise in the ICU.
ICUAP_12	I believe a “quieter” space provides clinicians with an increased ability to think.
ICUAP_13	I believe the quality of my work is negatively impacted by a noisy environment.
ICUAP_14	I believe noise could contribute to medical errors.
ICUAP_15	I believe noise decreases my sense of connection with patients.
ICUAP_16	I believe noise decreases staff sense of satisfaction.
ICUAP_17	I believe noise negatively impacts on patient sleep quality in the ICU.

## Findings

### Survey Analysis

Out of a potential sample size of 200, a total of 74 responses were received, resulting in a response rate of 37%. Among the participants, registrars represented the highest designation category, accounting for 24% (*N* = 18), followed by consultants at 23% (*N* = 17; Table 3). Only 73 participants rated their experience and their ICU experience varied, with approximately 18% (*N* = 13) having more than 20 years of experience, 23% (*N* = 17) with 10–19 years, 37% (*N* = 27) with less than 5 years, and the remaining 22% (*N* = 16) falling into the 6–9 years category. Females comprised 56.8% of the participants (*N* = 42), and males accounted for 40.5% (*N* = 30). The two remaining participants (2.7%) did not disclose their gender.

**Table 3.** Questionnaire Survey Participants’ Demographics

Description	Frequency	Percentage	Cumulative Percentage
Designation/role			
Consultant	17	23	23
Allied health professional	13	17.6	40.5
Registrar	18	24.3	64.9
Registered nurse	13	17.6	82.4
Clinical nurse	9	12.2	94.6
Resident medical officer	2	2.7	97.3
Clinical nurse specialist/nurse unit manager	2	2.7	100
Total	74	100	
Years of ICU experience			
<5	27	37	37
6–9	16	21.9	58.9
10–19	17	23.3	82.2
20+	13	17.8	100
Total	73	100	
Gender			
Male	30	40.5	40.5
Female	42	56.8	97.3
Nonbinary	0	0	97.3
Prefer not to say	2	2.7	100.0
Total	74	100	

#### Data suitability

Under data suitability, missing data entities and outliers were examined. Seven ICUAP variables had no missing data entities, while nine variables had one missing value. Only one variable had two missing entities. Considering this, the effect of missing data was negligible. The boxplots and histograms did not indicate outliers. The ICUAP data distributions were not precisely normal according to the Shapiro–Wilk test (*p* < .05), but the kurtosis and skewness values were less than |1| for ICUAPs, except for ICUAPs 09 and 10. Agreeing with these numerical tests, the Q-Q plots showed no significant deviations from straight lines for these 15 variables, indicating approximate normality. Thus, they were tested using parametric tests while nonparametric alternatives were used for ICUAPs 09 and 10 (Kwak & Kim, 2017; Matulová & Rejentová, 2021; Razali & Wah, 2011).

#### Participant’s perception of ICUAPs: Testing H1

Table 4 illustrates the descriptive statistics of ICUAPs and the outcomes of the one-sample *t* test/Wilcoxon–signed rank test results for H1. Based on the sample data, the population central tendencies were hypothesized as shown in the table. As the given *p* values are greater than .05, there was no statistically significant difference between the population mean/median and hypothesized values.

**Table 4.** Sample and Population Central Tendencies of ICU Acoustic Performances (ICUAPs).

Factor ID	Sample Data		Hypothesized mean^a^	*t*-Test Outcomes^b^			Participant’s Agreement-Level Toward ICUAP
	Mean^a^	Standard Deviation		*t*-Value	*df*	*p* Value	
ICUAP 01	5.10	1.386	5	0.591	72	0.556	The study population “somewhat agree” that impact of noise in the ICU is different to other areas such as trauma and emergency
ICUAP 02	5.34	1.325	5.5	−1.016	72	0.313	The study population is in between “somewhat agree” and “agree” that noise levels in the ICU negatively impact on clinical care.
ICUAP 03	4.21	1.510	4	1.171	71	0.246	The study population “neither disagree nor agree” that noise levels in the ICU negatively impact staff well-being.
ICUAP 04	6.42	0.759	6.5	−0.919	73	0.361	The study population is in between “agree” and “strongly agree” that noise negatively impacts on patient sleep quality in the ICU.
ICUAP 05	4.12	1.526	4	0.686	73	0.495	The study population “neither disagree nor agree” that patient procedures are a significant source of noise in the ICU.
ICUAP 06	4.55	1.536	4.5	0.303	73	0.763	The study population is in between “neither disagree nor agree” and “somewhat agree” that managing ventilation and life-support equipment is a significant source of noise in the ICU.
ICUAP 07	4.45	1.366	4.5	−0.340	73	0.735	The study population is in between “neither disagree nor agree” and “somewhat agree” that visitors such as family members and/or ancillary staff are a significant source of noise in the ICU.
ICUAP 08	4.34	1.493	4.5	−0.902	72	0.370	The study population is in between “neither disagree nor agree” and “somewhat agree” that they communicate safely and effectively without disrupting patient sleep.
ICUAP 09	6	N/A	6	N/A		0.341	The study population “agree” that *alarms* are a significant source of noise in the ICU.
ICUAP 10	5	N/A	5	N/A		.912	The study population “somewhat agree” that *talking* between clinicians is a significant source of noise in the ICU.
ICUAP 11	4.96	1.348	5	−0.260	72	0.795	The study population “somewhat agree” that the way in which the ICU is designed helps them to perform their duties.
ICUAP 12	4.77	1.429	5	−1.392	72	0.168	The study population “somewhat agree” that the ICU allows them to communicate efficiently with other staff in the ICU.
ICUAP 13	5.76	0.991	6	−2.112	73	0.058	The study population “agree” that a “quieter” space provides clinicians with an increased ability to think.
ICUAP 14	5.04	1.467	5	0.239	72	0.811	The study population “somewhat agree” that the quality of my work is negatively impacted by a noisy environment.
ICUAP 15	5.47	1.068	5.5	−0.274	72	0.785	The study population is in between “somewhat agree” and “agree” that noise could contribute to medical errors.
ICUAP 16	4.77	1.267	5	−1.560	73	0.123	The study population “somewhat agree” that noise decreases their sense of connection with patients.
ICUAP 17	4.80	1.182	5	−1.475	73	0.144	The study population “somewhat agree” that noise decreases staff sense of satisfaction.

^a^ Median used to measure central tendency of ICUAP 09 and ICUAP 10.

^b^ One-sample Wilcoxon-signed rank test used to test H1 for ICUAP 09 and ICUAP 10.

ICUAPs 01–04 examined the effects of noise in Fiona Stanley Hospital’s ICU. Participants somewhat agreed (µ = 5) that noise in the ICU differs from other hospital areas (ICUAP 01). The greatest concern was the negative impact on patient sleep quality (ICUAP 04), rated at µ = 6.5. The negative impact on staff well-being (ICUAP 03) was of least concern (µ = 4). The impact of noise on overall clinical care (ICUAP 02) was rated at µ = 5.5.

ICUAPs 05–10 assessed staff perceptions of noise sources. Alarms (ICUAP 09) were considered the most significant noise source (median = 6), followed by clinicians’ conversations (ICUAP 10), with a median rating of “somewhat agree” (median = 5). Ventilation and life-support equipment (ICUAP 06), visitors (ICUAP 07), and staff’s ability to communicate without disrupting patient sleep (ICUAP 08) were rated at µ = 4.5. Participants neither agreed nor disagreed (µ = 4.0) that patient procedures (ICUAP 05) were a significant source of noise. ICUAPs 11–17 explored staff perceptions of current working conditions and potential areas for improvement. ICU design was seen to facilitate duties (ICUAP 11) and efficient communication among staff (ICUAP 12). The quality of work was somewhat negatively impacted by ICU noise (ICUAP 14), and noise could contribute to medical errors (ICUAP 15). A quieter ICU was desired for improved thinking ability (ICUAP 13), connection with patients (ICUAP 16), and job satisfaction (ICUAP 17), all rated between “somewhat agree” and “agree” (µ = 5.5–6.0).

#### Analysis of variance—Testing H2

As mentioned earlier, H2 tested the effect of ICU experience toward participant’s perceptions of ICUAPs. Table 4 indicates only statistically significant outcomes based on one-way analysis of variance. ICU experience influenced ICUAP 03 (*F* = 3.209, *df* = .03, *p* = .028), and this implies that ICUAP 03 ratings were different at least between two groups. While using the 20+ category as the control group, a Dunnett *t* test was conducted to compare all other groups against it. The test outcome showed that only people with less than 5 years of ICU experience considered ICUAP 03 less significant than the 20+ category (*p* = .046).

### Existing Noise Levels

The study revealed that average noise levels (Leq) during the entire monitoring period inclusive of times when there were no patients present were 53.6 dB(A) (standard deviation of 3.0) during the day and 48.5 dB(A) (standard deviation of 5.0) at night. The average noise levels taken only when the room was occupied by patients during the day (Leq (Day)) and night (Leq (Night)) was Leq 55.8 dB(A) during the day (7 a.m. to 10 p.m.), and Leq 50.4 dB(A) at night (10 p.m. to 7 a.m.).

These findings indicate that the noise levels exceed the criteria established by AS/NZS 2016:2017 and the WHO Guidelines, although the daytime Leq levels are slightly lower compared to other Australian ICUs (Elliott et al., 2010). The maximum noise levels (Lmax) in the ICU room are primarily caused by alarms from medical equipment, discussions among clinical staff, and patient vocalization. The highest Lmax (Fast) levels recorded in 15-min intervals ranged between Lmax 60 and 90 dB(A) when patients were present (see Online Appendix A), and a mean noise level Lmax 74 dB(A) was detected over the monitoring period. The measured maximum noise levels significantly exceed the recommended criteria for sleep disruption (Lmax 50–55 dB(A)).

### Acoustic Calculation of Proposed Model Option “A”

Four scenarios were modeled with equipment positioning specified: the existing suite with the Hamilton S1 ventilator alarm, the existing suite with the Philips MX-800 Patient Monitor alarm, upgraded Design Option “A” suite with the Hamilton S1 ventilator alarm, and upgraded Design Option “A” suite with the Philips MX-800 Patient Monitor alarm. Note, the AS/NZS 2107:2016 noise criteria were not directly relevant, relating only to steady-state and quasi steady-state noise sources, while the WHO Guidelines establish criteria for intermittent noise sources; an Lmax(F) criteria of 40 dB(A) at night, however, it is applicable to intermittent noise sources.

**Figure 5.**
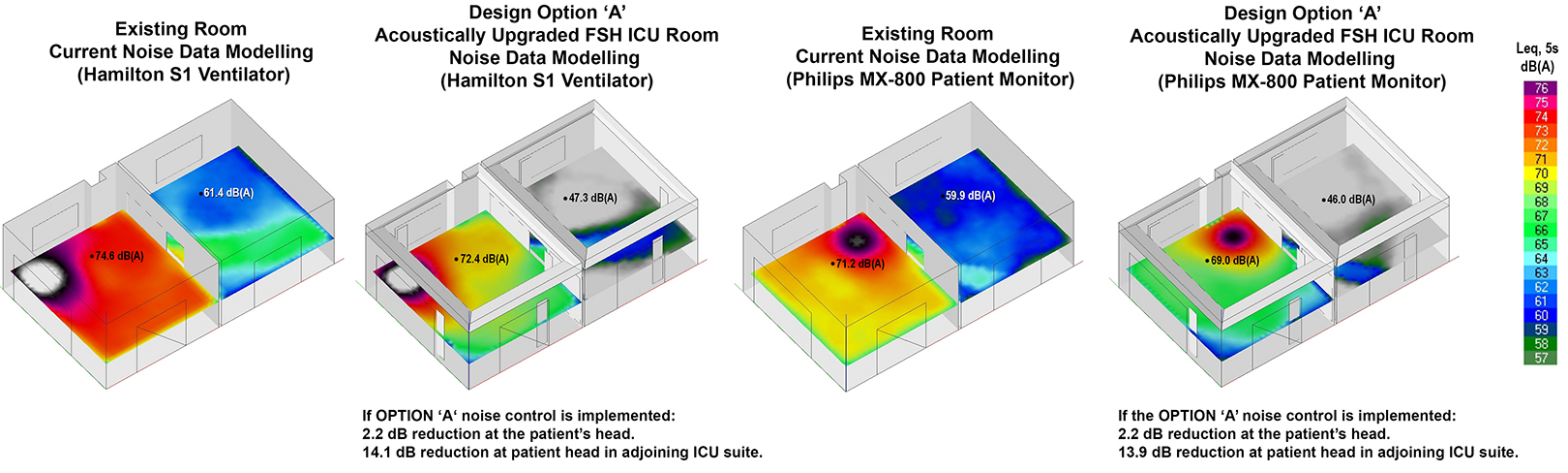
Ray-tracing ICU room modeled including the adjoining suite.

Design Option “A” achieved a modest noise reduction of slightly over 2 dB at the patient’s head position, effectively mitigating reflected noise. It did not address the direct noise transmission path from equipment to the patient. Notably, Option “A” resulted in a substantial 14 dB reduction in noise reverberation and transmission into the adjoining suite with an open sliding door. Figure 5 illustrates the noise model.

## Discussion

### Design Modeling Option “A”

The preferred prototype—“Option ‘A’”—represents a typical single-occupancy ICU room designed as an open-plan environment at a microscale. The significance of acoustic design in minimizing noise disturbances and optimizing the healthcare environment for patient well-being and recovery has been highlighted in previous studies (Devlin et al., 2018;Litton et al., 2021; Litton et al., 2017; Wibrow et al., 2022). Moreover, the findings from ICUAPs 05–10 underscore the significance of staff perceptions regarding the multifaceted nature of noise in the ICU. The identified sources of noise, including alarms, clinician communication, patient procedures, and equipment management, align with existing literature and studies, such as Ruettgers et al. (2022), which emphasize the disruptive nature of short-lasting vocalizations among healthcare professionals. The identification of alarms (ICUAP 09) as the most significant noise source underscores the importance of addressing their impact on the overall noise environment in the ICU. In response to this, “Option A” was designed to specifically target the effects of such noise sources. This strategic approach is crucial for enhancing the acoustic environment within the ICU and falls within the purview of the architect to attempt to “design-out” these noise-related challenges.

By focusing on mitigating the impact of alarms through design interventions, “Option A” aligns with the understanding that the architect plays a pivotal role in shaping the built environment to optimize functionality and well-being. The ability to “design-out” the impact of noise sources, especially those identified as prominent contributors, reflects a proactive and targeted effort to create a more conducive and supportive environment for both staff and patients within the ICU. Further to this, the design of the ICU, as highlighted in ICUAPs 12, was perceived to facilitate duties and promote efficient communication among staff. Findings from ICUAP 14 and ICUAP 15 highlight the negative impact of noise on clinician–patient connection, and the quality of work, with noise being considered a potential contributor to medical errors.

Notably, participants expressed a desire for a quieter ICU environment to mitigate ICUAPs 13, 16, and 17—all associated with improved thinking ability, enhanced connection with patients, and increased job satisfaction. These findings resonate with research by Schmidt et al. (2020), supporting the notion that heightened noise levels can lead to reduced clinician well-being and attention, as well as an increase in self-assessed distraction and performance challenges.

To address these concerns, this study achieved an optimal combination of high sound absorption and sound attenuation to effectively control excessive sound reflections. Given the proximity to adjoining single-occupancy rooms, effective sound transmission control was necessary to ensure privacy, confidentiality, and minimize disturbances. Spaces with high traffic or multipurpose usage require sufficient sound absorption and sound blocking measures to control noise levels and prevent disruptions to adjacent areas. Within a clinical setting, comprehensive acoustic control plays a vital role in minimizing cognitive degradation (Helton et al., 1980; Little et al., 2012; Orr & Stahl, 1977), crosstalk, excessive reverberation, ensure privacy, maintain confidentiality, and minimize disturbances for both staff and patients. These factors underscore the importance of implementing comprehensive acoustic strategies to create a healing environment and promote patient-centered care. The NRC quantifies the sound absorption of a surface material on a scale from 0.0 to 1.0. An NRC of 1.0 indicates 100% sound absorption, while an NRC of 0.7 (as found in this study) means 70% of the sound is absorbed and 30% is reflected.

### Surface Finish Considerations

ICU rooms typically have acoustically absorbent ceiling tiles, but their acoustic performance can vary based on site-specific specifications, installation requirements, and plenum depth. Additionally, infection control requirements must be considered when specifying sound absorption panels on all surfaces while hygiene standards for healthcare flooring solutions do little for noise reduction. A single fusion rubber underlaid resilient floor finish shows significant noise reduction without compromising hygiene standards (Huxta et al., n.d.; Paul et al., 2014). While it effectively reduces noise from rolling carts and footfalls, it does not improve airborne sound absorption from equipment alarms or staff conversations.

This study identified various areas for noise mitigation and highlight four methods for reducing noise exposure to ICU patients, including (1) reducing noise at the source, (2) general attenuation, (3) attenuation at the receiver location, and (4) attenuation to the direct and reflected transmission paths. The study particularly focused on strategies that offer significant noise reduction performance relative to cost. Three technical design options were developed, each with associated substrategies and a proposed material specification schedule. These solutions aim to comply with Health Insurance Portability and Accountability Act of 1996 (HIPAA), Hospital Consumer Assessment of Healthcare Providers and Systems (HCAHPS), National Construction Code (NCC), and International Organization for Standardization (ISO) requirements while considering maintainability and durability. Table 5 highlights the methods and substrategies considered in each design option.^7,8^ Option A was preferred for several reasons and an OPC with recommendations for economic implications, including cost-effectiveness was developed based on Option A only. Justification for excluding Options “B” and “C” include practicality and safety. For instance, Options B and C contemplated widening and chamfering the walls, leading to increased material costs. However, these modifications yielded minimal benefits and posed practical issues, such as the risk of trip hazards from extended cables and tubes for patients and clinicians, potentially compromising their safety. Additionally, flooring specifications in Options B and C resulted in higher costs but were unlikely to significantly reduce machine and vocalization noise.

**Table 5.** Methods and Substrategies (Potential and Implemented).

Method	Substrategy	Implemented in Fiona Stanley Hospital ICU
1. Reduce noise at source: Alarm volumes, loudness of spoken word	Equipment speaker design and orientation: Speakers assumed to be unidirectional. Note: Frequency and dB design (not part of this study)	No
2. General attenuation: Spatial modeling and materials	Walls: Acoustic panels, assume infection control, impact resistant, cleanable, durable to achieve claimed 90% Noise Reduction Coefficient (NRC) or higher	Yes
	Ceiling profile and material: Suspended grid ceiling with a concealed edge to achieve claimed 90% NRC or higher (assumes compliant fire rating)	Yes
	Antimicrobial acoustic curtains ^a^: Hemp disposable/washable, fire rated, antibacterial and anti-mildew chemical and nanometer silver impregnated (in front of all glazing)	Yes
	Physical layout: Chamfered wall—wall/wall—ceiling junctions	Yes
3. Attenuation at receiver	Patient earplugs	No
4. Attenuation barriers between transmission paths to patient ear	Acoustic barrier between patient and noise source	No

^a^ Antimicrobial acoustic curtains, due to its fibrous and porous structure, have high NRC values and can be used as a sound-absorbing material. It is a natural, eco-friendly, and sustainable alternative to traditional sound-absorbing materials like fiberglass or polyurethane foam. More research is needed to establish Hemp’s capabilities as a sound-absorbing curtain material in comparison to other more traditional materials available in the market.

While a 14 dB reduction in noise transmission from medical equipment to the adjoining suite (open sliding door between) is significant, the patient in the adjoining room will still be exposed to the noise emissions from the medical equipment within their own suite. This is where consideration is needed in relation to direct noise transmission from the medical equipment to the patient as discussed below. Notwithstanding the above, if the sound absorption treatment can significantly address the medical equipment noise from the adjoining ICU suite, this will still reduce the number of peak noise events that the patient is exposed to.

A greater noise reduction could be achieved if the medical equipment were positioned further away from the patient’s head; however, this is not feasible in a clinical setting due to operational requirements. Furthermore, it is not practicable to install a physical noise barrier between the medical equipment and the patients head in order to reduce the direct noise transmission. Adjusting the volume of these items to a medium setting could significantly reduce the maximum noise levels (Lmax) in ICU rooms. If determined to be safe through further studies, utilizing a medium volume setting instead of the maximum on ICU equipment could potentially yield significant noise reductions of over 15 dB. There may also be the potential in the future for manufacturers to utilize alarm tones which are more directional or incorporates beam forming such that the alarms are less audible at the patient’s bed.

### Cost Calculation of Proposed Model^9^

The cost difference between a standard ICU and an acoustically treated ICU like Option “A” depends on various factors, including size, required treatment extent, materials used, labor costs, and location. Generally, acoustically treated ICU rooms are more expensive due to additional materials and installation processes for desired acoustic performance. The OPC (Table 6) is indicative and assumes conventional procurement processes, market forces, and project-specific factors. It breaks down Alterations and Renovations, Wall Finishes, and Ceiling Finishes for Option “A.” Exclusions were noted. As the cost-effectiveness of high-spec acoustic flooring was found to have limited impact on reducing machine-generated and vocalization noises while significantly increasing costs. This intervention is likely to have a minimal effect on airborne noises but can have a more pronounced effect on impact noises on the floor surface. These cost-effectiveness considerations are reflected in the table, which presents two alternatives: Alternative 1, without high-spec acoustic flooring, and Alternative 2, without high-spec acoustic flooring and without extra-over ceiling chamfering. These alternatives aim to showcase the potential cost savings associated with different acoustic flooring products.

**Table 6.** Opinion of Probable Cost for Alternatives Design Implementation Options (Australian Dollars (AUD)).

	As Specified (Costed as per Full Option “A” Specification [Online Appendix C])	Alternative 1 (Costed with High-Spec Acoustic Flooring Omitted)	Alternative 2 (Costed With High-Spec Acoustic Flooring, and Ceiling Chamfering Omitted)
Fitout cost difference per room between the study-specified finish and a “Base Build” nonacoustically treated ICU room (applied in a *New* hospital build scenario)	$66,000	$31,200	$25,200
Fitout cost difference per room between the study-specified finish and a “Base Build” nonacoustically treated ICU room (applied in a *Retrofit* scenario)	$73,000	$36,800	$30,800

Based on the analysis, constructing a “test pod” within a *new* ICU project was determined to be the most cost-effective approach to achieving Option “A” compared with retrofitting, which involves demolition and “make good” costs and generates waste, incurring further costs.^10^ The proposal for a “test pod” is based on a cost-effectiveness analysis, indicating that it could be the most financially viable approach when compared to retrofitting. While the mathematical analysis favors the “test pod” as a cost-effective solution, the study recognizes the need for a balanced approach that combines cost-effectiveness with practicality. Therefore, it suggests an initial phase involving a one-off retrofit to assess the feasibility of noise reduction strategies. This preliminary step will allow us to validate the effectiveness of our approach without committing to the construction of a “test pod.” The findings from this one-off retrofit will serve as a crucial litmus test, enabling us to make informed decisions and tailor future approaches to meet the specific needs of the ICU environment. By adopting this measured approach, the study aims to ensure not only cost-effectiveness but also the highest quality of care for patients, while considering the long-term implications of noise reduction in healthcare settings.

Although this study has provided valuable insights into the impact of noise in ICU environments, it is essential to acknowledge certain limitations. First, the study focused on a specific type of ICU room, and the findings may not be universally applicable to all ICU settings.

This must be considered particularly when the outcomes of H2 is interpreted. During the iterative questionnaire design, it was expected by the subject matter experts that the ICU experience would influence participant’s opinions of ICUAPs. Nevertheless, only ICUAP 03 had a statistically significant relationship with the amount of ICU experience. Future studies with a larger and more diverse population are recommended to substantiate this finding.

However, the findings will be significant to other Australian hospitals due to the compliance requisite of meeting acoustic performance outlined in the NCC. The NCC typically requires hospitals to meet specific Sound Transmission Class and Impact Insulation Class ratings for walls and floors to minimize sound transmission between spaces. These ratings help ensure patient privacy and reduce disturbance, as well as room acoustics to ensure that speech intelligibility is maintained. This is particularly important in patient rooms, where effective communication with medical staff is vital. As the questionnaire data collection was limited to the ICU employees working in Fiona Stanley hospital, the sample size of patients and healthcare professionals involved in surveys and data collection was limited, which may affect the generalizability of the results. Additionally, the study primarily relied on self-reported data, which could introduce a level of subjectivity. Finally, while efforts were made to control for confounding factors, there may be other variables not accounted for in the analysis. Recognizing these limitations, we believe our findings provide a valuable foundation for future research and the development of noise reduction strategies in ICU environments.

## Conclusion

This study adds to the existing knowledge on ICU noise mitigation, focusing on design aspects. It found that room layout and equipment–patient distance limit noise reduction options to perimeter sound absorption, direct transmission pathways, or volume settings with potential for significant noise reductions. Further studies are needed to balance reduced alarm volume with staff supervision requirements.

Without an acoustic barrier, significant acoustic treatments in ICUs may yield conservative noise reduction outcomes. Future research should explore methods to mitigate direct noise transmission between equipment and patients, especially in the presence of controlled reverberant noise like Design Option “A.”

Design Option “A” was predicted to substantially reduce noise transmission into adjoining suites when the sliding door is open, benefiting clinical care and resource utilization. The most efficient approach to implementing a similar ICU design is within a new project as it excludes additional costs such as demolition, reinstatement, labor, material costs, and waste associated with retrofitting.

There is potential for medical equipment designers to modify alarm speakers’ directivity, directing the sound toward sound-absorbing wall finishes and reducing noise exposure toward patients. This study contributes to the discourse on ICU design for improved patient sleep and holds promise for future design and clinical studies in healthcare settings.

## Implications for Practice

This research has implications for practice in several ways.

1. Industry-based research highlight the importance of multidisciplinary research, bringing together complementary skills in healthcare, design, acoustics, statistics, and quantity surveying to address important design challenges.
2. Evidence-based research: The approach provides a informed spatial design modeling and specifications for a proposed single-occupancy ICU room.
3. Material research and design approaches: The study involves material research and design approaches that needed to be projected, quantified, and their possible impacts understood.
4. Implement effective acoustic treatments incorporate findings to select appropriate materials and design strategies, such as perimeter sound absorption, direct noise transmission pathways, and volume control, to significantly reduce noise levels in ICU rooms, improving patient sleep and well-being.
5. Plan room layout and equipment placement carefully consider the proximity of noise-generating equipment to patient beds, creating adequate space between patients and equipment to minimize direct noise transmission and enhance patient comfort.
6. Utilize acoustic barriers incorporate acoustic barriers, such as soundproof walls and ceilings, to mitigate sound travel between noise sources and patient ears, reducing noise transmission and improving patient restfulness.
7. Consider glazed partition doors: This includes glazed partition doors between ICU rooms, allowing visual supervision while minimizing noise transmission, enhancing patient care and staff efficiency.
8. Collaborate with equipment designers: This explores collaborations with medical equipment designers to develop innovative solutions, such as modifying alarm speakers to direct sound toward sound-absorbing surfaces, reducing noise exposure and improving the acoustic environment in ICUs.

## Supplemental Material

Supplemental Material, sj-pdf-1-her-10.1177_19375867241237501 - Mitigating Intensive Care Unit Noise: Design-Led Modeling Solutions, Calculated Acoustic Outcomes, and Cost Implications Supplemental Material, sj-pdf-1-her-10.1177_19375867241237501 for Mitigating Intensive Care Unit Noise: Design-Led Modeling Solutions, Calculated Acoustic Outcomes, and Cost Implications by Emil E. Jonescu, Benjamin Farrel, Chamil Erik Ramanayaka, Christopher White, Giuseppe Costanzo, Lori Delaney, Rebecca Hahn, Janet Ferrier and Edward Litton in HERD: Health Environments Research & Design Journal

Supplemental Material, sj-pdf-2-her-10.1177_19375867241237501 - Mitigating Intensive Care Unit Noise: Design-Led Modeling Solutions, Calculated Acoustic Outcomes, and Cost Implications Supplemental Material, sj-pdf-2-her-10.1177_19375867241237501 for Mitigating Intensive Care Unit Noise: Design-Led Modeling Solutions, Calculated Acoustic Outcomes, and Cost Implications by Emil E. Jonescu, Benjamin Farrel, Chamil Erik Ramanayaka, Christopher White, Giuseppe Costanzo, Lori Delaney, Rebecca Hahn, Janet Ferrier and Edward Litton in HERD: Health Environments Research & Design Journal

Supplemental Material, sj-pdf-3-her-10.1177_19375867241237501 - Mitigating Intensive Care Unit Noise: Design-Led Modeling Solutions, Calculated Acoustic Outcomes, and Cost Implications Supplemental Material, sj-pdf-3-her-10.1177_19375867241237501 for Mitigating Intensive Care Unit Noise: Design-Led Modeling Solutions, Calculated Acoustic Outcomes, and Cost Implications by Emil E. Jonescu, Benjamin Farrel, Chamil Erik Ramanayaka, Christopher White, Giuseppe Costanzo, Lori Delaney, Rebecca Hahn, Janet Ferrier and Edward Litton in HERD: Health Environments Research & Design Journal

Supplemental Material, sj-pdf-4-her-10.1177_19375867241237501 - Mitigating Intensive Care Unit Noise: Design-Led Modeling Solutions, Calculated Acoustic Outcomes, and Cost Implications Supplemental Material, sj-pdf-4-her-10.1177_19375867241237501 for Mitigating Intensive Care Unit Noise: Design-Led Modeling Solutions, Calculated Acoustic Outcomes, and Cost Implications by Emil E. Jonescu, Benjamin Farrel, Chamil Erik Ramanayaka, Christopher White, Giuseppe Costanzo, Lori Delaney, Rebecca Hahn, Janet Ferrier and Edward Litton in HERD: Health Environments Research & Design Journal

Supplemental Material, sj-pdf-5-her-10.1177_19375867241237501 - Mitigating Intensive Care Unit Noise: Design-Led Modeling Solutions, Calculated Acoustic Outcomes, and Cost Implications Supplemental Material, sj-pdf-5-her-10.1177_19375867241237501 for Mitigating Intensive Care Unit Noise: Design-Led Modeling Solutions, Calculated Acoustic Outcomes, and Cost Implications by Emil E. Jonescu, Benjamin Farrel, Chamil Erik Ramanayaka, Christopher White, Giuseppe Costanzo, Lori Delaney, Rebecca Hahn, Janet Ferrier and Edward Litton in HERD: Health Environments Research & Design Journal

Supplemental Material, sj-pdf-6-her-10.1177_19375867241237501 - Mitigating Intensive Care Unit Noise: Design-Led Modeling Solutions, Calculated Acoustic Outcomes, and Cost Implications Supplemental Material, sj-pdf-6-her-10.1177_19375867241237501 for Mitigating Intensive Care Unit Noise: Design-Led Modeling Solutions, Calculated Acoustic Outcomes, and Cost Implications by Emil E. Jonescu, Benjamin Farrel, Chamil Erik Ramanayaka, Christopher White, Giuseppe Costanzo, Lori Delaney, Rebecca Hahn, Janet Ferrier and Edward Litton in HERD: Health Environments Research & Design Journal

Supplemental Material, sj-pdf-7-her-10.1177_19375867241237501 - Mitigating Intensive Care Unit Noise: Design-Led Modeling Solutions, Calculated Acoustic Outcomes, and Cost Implications Supplemental Material, sj-pdf-7-her-10.1177_19375867241237501 for Mitigating Intensive Care Unit Noise: Design-Led Modeling Solutions, Calculated Acoustic Outcomes, and Cost Implications by Emil E. Jonescu, Benjamin Farrel, Chamil Erik Ramanayaka, Christopher White, Giuseppe Costanzo, Lori Delaney, Rebecca Hahn, Janet Ferrier and Edward Litton in HERD: Health Environments Research & Design Journal

## Notes

1. The study assesses in-home traffic-related disturbance and not in an ICU or clinical environment.

2. The standard does not apply to activity or equipment noise such as alarms and people speaking.

3. Data presented in Appendix A as time-profile charts for each day. Charts patterns highlight occupancy and vacancy.

4. For a comprehensive list and guidelines, see https://healthfacilityguidelines.com.au/

5. Floor finish slip rating (R10; Pendulum P3 or agreed equivalent) could not be verified during the site visit.

6. Lighting fluorescent/LED with a flush finish to the ceiling surface; a window bringing in natural light into the room is glazed, in aluminum frame with inset blinds; and curtain privacy screens were *not* installed in the room.

7. For example, large surface areas such as walls and ceilings with higher NRC potential was considered more favorably when compared with the potential contribution of available floor finish products (which introduce additional operational performance challenges) and glazing (which is typically a poor absorber of sound unless the surface is treated), and interlayers and multiple panels creating air gaps between panes typically act to reduce transmission to adjacent areas rather than absorb sound in the space requiring attenuation.

8. The way in which sound waves interact with glass may provide an insignificant benefit; however, their cumulative inclusion among other substrategy considerations were not completely discounted but were reserved should further sound attenuation be required or demonstrated to be achieved. Nano-etched (nanopores created by the union of magnetic and plasmonic activities), textured glass, or an applied absorbent film (to diffract sound waves) may be possible but not cost-effective.

9. The Western Australian construction market is considered as extremely volatile (at the time of the study). Skilled labor shortages have led to notable cost escalation increases since the 2021 calendar year, and the escalation allowances included in this study align with Australian Institute of Quantity Surveyors (AIQS) publications estimates.

10. Floor finishes in Option “A” were deemed to have limited sound absorption properties with insignificant measurable impact on in-room noise levels, as such the study assumed no changes to standard vinyl finishes.

## References

[bibr1-19375867241237501] Australasian Health Facility Guidelines. (2019). AusHFG. http://www.healthfacilityguidelines.com.au/

[bibr2-19375867241237501] Beltrami F. G. Nguyen X.-L. Pichereau C. Maury E. Fleury B. Fagondes S. (2015). Sleep in the intensive care unit. Jornal Brasileiro de Pneumologia, 41(6), 539–546. 10.1590/s1806-37562015000000056 26785964 PMC4723006

[bibr3-19375867241237501] Broussard J. L. Ehrmann D. A. Van Cauter E. Tasali E. Brady M. J. (2012). Impaired insulin signaling in human adipocytes after experimental sleep restriction. Annals of Internal Medicine, 157(8), 549–557. 10.7326/0003-4819-157-8-201210160-00005 23070488 PMC4435718

[bibr4-19375867241237501] Busch-Vishniac I. Ryherd E. (2023). Hospital soundscapes. In Schulte-Fortkamp B. Fiebig A. Sisneros J. A. Popper A. N. Fay R. R. (Eds.), Soundscapes: Humans and their acoustic environment. Springer. 10.1007/978-3-031-22779-0

[bibr6-19375867241237501] Cooper A. B. Thornley K. S. Young G. B. Slutsky A. S. Stewart T. E. Hanly P. J. (2000). Sleep in critically ill patients requiring mechanical ventilation. Chest, 117(3), 809–818. 10.1378/chest.117.3.809 10713011

[bibr7-19375867241237501] Daou M. Telias I. Younes M. Brochard L. Wilcox M. E. (2020). Abnormal sleep, circadian rhythm disruption, and delirium in the ICU: Are they related? Frontiers in Neurology, 11. 10.3389/fneur.2020.549908 PMC7530631 33071941

[bibr8-19375867241237501] Darbyshire J. L. Müller-Trapet M Cheer J. Fazi F. M. Young J. D. (2019). Mapping sources of noise in an intensive care unit. Anaesthesia, 74(8), 1018–1025. 10.1111/anae.14690 31066046 PMC6767712

[bibr9-19375867241237501] Delaney L. J. Currie M. J. Huang H.-C. C. Lopez V. Litton E. Van Haren F. (2017). The nocturnal acoustical intensity of the intensive care environment: An observational study. Journal of Intensive Care, 5(1). 10.1186/s40560-017-0237-9 PMC5504755 28702196

[bibr10-19375867241237501] Devlin J. W. Skrobik Y. Gélinas C. Needham D. M. Slooter A. J. C. Pandharipande P. P. Watson P. L. Weinhouse G. L. Nunnally M. E. Rochwerg B. Balas M. C. van den Boogaard M. Bosma K. J. Brummel N. E. Chanques G. Denehy L. Drouot X. Fraser G. L. Harris J. E. Joffe A. M. (2018). Clinical practice guidelines for the prevention and management of pain, agitation/sedation, delirium, immobility, and sleep disruption in adult patients in the ICU. Critical Care Medicine, 46(9), e825–e873. 10.1097/ccm.0000000000003299 30113379

[bibr11-19375867241237501] Elliott R. McKinley S. Cistulli P. Fien M. (2013). Characterisation of sleep in intensive care using 24-hour polysomnography: An observational study. Critical Care, 17(2), R46. 10.1186/cc12565 23506782 PMC3733429

[bibr12-19375867241237501] Elliott R. McKinley S. Eager D. (2010). A pilot study of sound levels in an Australian adult general intensive care unit. Noise and Health, 12(46), 26. 10.4103/1463-1741.59997 20160388

[bibr14-19375867241237501] Helton M. C. Gordon S. H. Nunnery S. L. (1980). The correlation between sleep deprivation and the intensive care unit syndrome. Heart & Lung: The Journal of Critical Care, 9(3), 464–468. 6901518

[bibr15-19375867241237501] Huxta M. Paley S. (n.d.). Managing noise in the healthcare space through flooring specification. ECOsurfaces. Retrieved January 11, 2023, from https://ecosurfaces.com/sites/default/files/documents/ECO_WhitePaper_NoiseHealthcare.pdf

[bibr16-19375867241237501] IBM Corp. (2021). BM SPSS Statistics for Windows, Version 28.0.

[bibr17-19375867241237501] Johansson L. Lindahl B. Knutsson S. Ögren M. Persson Waye K. Ringdal M. (2018). Evaluation of a sound environment intervention in an ICU: A feasibility study. Australian Critical Care, 31(2), 59–70. 10.1016/j.aucc.2017.04.001 28506741

[bibr18-19375867241237501] Kwak S. G. Kim J. H. (2017). Central limit theorem: The cornerstone of modern statistics. Korean Journal of Anesthesiology, 70(2), 144–156. 10.4097/kjae.2017.70.2.144 28367284 PMC5370305

[bibr20-19375867241237501] Little A. Ethier C. Ayas N. Thanachayanont T. Jiang D. Mehta S. (2012). A patient survey of sleep quality in the intensive care unit. Minerva Anestesiologica, 78(4), 406–414. 22337154

[bibr200-19375867241237501] Litton E. (2023). Teleconference with Hames Sharley [Personal communication].

[bibr21-19375867241237501] Litton E. Atkinson H. Anstey J. Anstey M. Campbell L. T. Forbes A. Hahn R. Hooper K. Kasza J. Knapp S. McGain F. Ngyuen N. Pilcher D. Reddi B. Reid C. Robinson S. Thompson K. Webb S. Young P. (2021). Optimising a targeted test reduction intervention for patients admitted to the intensive care unit: The targeted intensive care test ordering cluster trial intervention. Australian Critical Care, 34(5), 419–426. 10.1016/j.aucc.2020.11.003 33526330

[bibr22-19375867241237501] Litton E. Elliot R. Ferrier J. Webb S. A. R. (2017). Quality sleep using earplugs in the intensive care unit: The QUIET pilot randomised controlled trial. Critical Care and Resuscitation, 19(2), 128–133. 28651508

[bibr23-19375867241237501] Litton E. Elliott R. Thompson K. Watts N. Seppelt I. Webb S. A. R. (2017). Using clinically accessible tools to measure sound levels and sleep disruption in the ICU. Critical Care Medicine, 45(6), 966–971. 10.1097/ccm.0000000000002405 28362644

[bibr24-19375867241237501] Matulova M. Rejentova J. (2021). Efficiency of European airports: Parametric versus non-parametric approach. Croatian Operational Research Review, 12(1), 1–14. 10.17535/crorr.2021.0001

[bibr25-19375867241237501] McKinley S. Aitken L. M. Alison J. A. King M. Leslie G. Burmeister E. Elliott D. (2012). Sleep and other factors associated with mental health and psychological distress after intensive care for critical illness. Intensive Care Medicine, 38(4), 627–633. 10.1007/s00134-012-2477-4 22318635

[bibr266-19375867241237501] Moriyama S. Okamoto K. Tabira Y. Kikuta K. Kukita I. Hamaguchi M. Kitamura N. (1999). Evaluation of oxygen consumption and resting energy expenditure in critically ill patients with systemic inflammatory response syndrome. Critical Care Medicine, 27(10), 2133–2136. 10.1097/00003246-199910000-00009 10548194

[bibr26-19375867241237501] New South Wales Government Roads and Maritime Services. (2015). Noise criteria guideline. https://web.archive.org/web/20150916125904/http://www.rms.nsw.gov.au/documents/about/environment/noise-criteria-guideline-book.pdf

[bibr27-19375867241237501] Nilius G. Richter M. Schroeder M. (2021). Updated perspectives on the management of sleep disorders in the intensive care unit. Nature and Science of Sleep, 13, 751–762. 10.2147/nss.s284846 PMC8200142 34135650

[bibr28-19375867241237501] Orr W. C. Stahl M. L. (1977). Sleep disturbances after open heart surgery. The American Journal of Cardiology, 39(2), 196–201. 10.1097/00132586-197710000-00044 299975

[bibr29-19375867241237501] Orwelius L. Nordlund A. Nordlund P. Edell-Gustafsson U. Sjoberg F. (2008). Prevalence of sleep disturbances and long term reduced health-related quality of life after critical care: A prospective multicenter cohort study. Critical Care, 12(4), R97. 10.1186/cc6973 18673569 PMC2575585

[bibr30-19375867241237501] Paul A. L. Arena D. A. King E. A. Celmer R. LoVerde J. J. (2014). Contribution of floor treatment characteristics to background noise levels in health care facilities, Part 1. The Journal of the Acoustical Society of America, 136(4), 2219–2219. 10.1121/1.4900055

[bibr31-19375867241237501] Razali N. M. Wah Y. B. (2011). Power comparisons of Shapiro-Wil, Kolmogrov-Smirnov, Lilliefors and Anderson-Darling test. Journal of Statistical Modeling and Analytics, 2(1), 21–33.

[bibr32-19375867241237501] Reade M. C. Finfer S. (2014). Sedation and delirium in intensive care. New England Journal of Medicine, 370(16), 1566–1567. 10.1056/nejmc1402402 24738685

[bibr33-19375867241237501] Ruettgers N. Naef A. C. Rossier M. Knobel S. E. J. Jeitziner M.-M. Holtforth M. G. Zante B. Schefold J. C. Nef T. Gerber S. M. (2022). Perceived sounds and their reported level of disturbance in intensive care units: A multinational survey among healthcare professionals. PLoS One, 17(12), e0279603–e0279603. 10.1371/journal.pone.0279603 36584079 PMC9803129

[bibr34-19375867241237501] Schmidt N. Gerber S. M. Zante B. Gawliczek T. Chesham A. Gutbrod K. Müri R. M. Nef T. Schefold J. C. Jeitziner M.-M. (2020). Effects of intensive care unit ambient sounds on healthcare professionals: Results of an online survey and noise exposure in an experimental setting. Intensive Care Medicine Experimental, 8(1). 10.1186/s40635-020-00321-3 PMC7376325 32705428

[bibr35-19375867241237501] Tembo A. C. Parker V. Higgins I. (2013). The experience of sleep deprivation in intensive care patients: Findings from a larger hermeneutic phenomenological study. .Intensive and Critical Care Nursing, 29(6), 310–316. 10.1016/j.iccn.2013.05.003 23806731

[bibr36-19375867241237501] Tiong E. Seow O. Camburn B. Teo K. Silva A. Wood K. L. Jensen D. D. Yang M. C. (2019). The economies and dimensionality of design prototyping: Value, time, cost, and fidelity. Journal of Mechanical Design, 141(3). 10.1115/1.4042337

[bibr377-19375867241237501] Trivedi M. S. Holger D. Bui A. T. Craddock T. J. A. Tartar J. L. (2017). Short-term sleep deprivation leads to decreased systemic redox metabolites and altered epigenetic status. PLOS ONE, 12(7), e0181978. 10.1371/journal.pone.0181978 28738082 PMC5524320

[bibr38-19375867241237501] Weinhouse G. L. Schwab R. J. Watson P. L. Patil N. Vaccaro B. Pandharipande P. Ely E. W. (2009). Bench-to-bedside review: Delirium in ICU patients—Importance of sleep deprivation. Critical Care, 13(6), 234. 10.1186/cc8131 20053301 PMC2811939

[bibr39-19375867241237501] Wibrow B. Martinez F. E. Myers E. Chapman A. Litton E. Ho Kwok. M. Regli A. Hawkins D. Ford A. van Haren F. M. P. Wyer S. McCaffrey J. Rashid A. Kelty E. Murray K. Anstey M. (2022). Prophylactic melatonin for delirium in intensive care (pro-MEDIC): A randomized controlled trial. Intensive Care Medicine, 48(4), 414–425. 10.1007/s00134-022-06638-9 35220473

