# Supplementary material for: Mitigating Intensive Care Unit Noise: Design-Led Modeling Solutions, Calculated Acoustic Outcomes, and Cost Implications
Source: HERD. 2024 Mar 21;17(3):220–38. doi: 10.1177/19375867241237501 (PMC11457460; doi:10.1177/19375867241237501)
Supplement: Supplemental Material, sj-pdf-1-her-10.1177_19375867241237501 - Mitigating Intensive Care Unit Noise: Design-Led Modeling Solutions, Calculated Acoustic Outcomes, and Cost Implications [file sj-pdf-1-her-10.1177_19375867241237501.pdf]

Time	Duration	Name	LAeq (dB)	LAFMax (dE	Ln1 (1) (dB)	Ln2 (5) (dB)	Ln3 (10) (dI	Ln4 (50) (dI	Ln5 (90) (dI	Ln6 (95) (dI	Ln7 (99) (dI
16/8/2022 10:01	0:13:08	10	71.6	108.9	68.4	62.5	60.3	53.4	48.3	47.8	47.3
16/8/2022 10:15	0:15:00	11	53.5	79.6	64.3	56.3	53.9	49.2	47.6	47.4	47
16/8/2022 10:30	0:15:00	12	53.4	73.1	63.3	58	55.6	49.8	47.7	47.5	47
16/8/2022 10:45	0:15:00	13	57.4	76.8	70.2	63.7	58.5	49.1	47.8	47.6	47.3
16/8/2022 11:00	0:15:00	14	57.9	75.4	67.8	64.1	61.5	53.1	49.7	49.3	48.8
16/8/2022 11:15	0:15:00	15	54.3	75.1	64.1	59.2	56.9	50.6	49	48.8	48.5
16/8/2022 11:30	0:15:00	16	54.9	75.9	65.6	60.1	57.3	50.3	48.6	48.4	48.1
16/8/2022 11:45	0:15:00	17	55.4	77.2	65.8	60.4	57.6	50.8	49.1	48.9	48.7
16/8/2022 12:00	0:15:00	18	52.4	69	61.8	56.5	54.3	49.9	48.8	48.7	48.4
16/8/2022 12:15	0:15:00	19	52.1	67.5	60.4	55.8	53.8	50.2	49.1	49	48.7
16/8/2022 12:30	0:15:00	20	56.7	85.7	67.1	61.6	58.7	50.6	49.3	49.1	48.9
16/8/2022 12:45	0:15:00	21	55.9	74.3	66.1	61	58.5	51.6	50.1	49.9	49.5
16/8/2022 13:00	0:15:00	22	56.6	88.1	63.7	59.6	57.5	51.9	50.3	50.1	49.8
16/8/2022 13:15	0:15:00	23	57	77.8	66.8	62	59.6	53	50.3	50.1	49.7
16/8/2022 13:30	0:15:00	24	58.3	79.9	67.8	63.5	61.2	54.1	50.3	49.9	49.5
16/8/2022 13:45	0:15:00	25	58.3	77.5	68.1	63.6	61.2	53.9	50.2	49.8	49.4
16/8/2022 14:00	0:15:00	26	57.9	78.2	68.5	63.5	60.8	53.1	49.7	49.3	48.7
16/8/2022 14:15	0:15:00	27	55.7	76.4	67	60.8	57.8	51.1	48.5	48.2	47.8
16/8/2022 14:30	0:15:00	28	55.7	81.4	66.6	60.9	57.8	50.6	49	48.7	48.4
16/8/2022 14:45	0:15:00	29	54.5	76.6	64.1	59.5	56.3	50.9	49	48.8	48.5
16/8/2022 15:00	0:15:00	30	53.4	73.5	65.4	57.3	53.7	49.6	48.6	48.4	48.1
16/8/2022 15:15	0:15:00	31	51.9	76.8	58.5	53.3	52	50.2	49.7	49.5	48.7
16/8/2022 15:30	0:15:00	32	52.1	69.6	60.3	55.3	53.4	50.4	49.7	49.6	49.3
16/8/2022 15:45	0:15:00	33	53.9	75	64.1	58.2	55.2	50.6	49.7	49.5	49.2
16/8/2022 16:00	0:15:00	34	52.1	67.9	58.9	55.9	54.5	50.4	49.4	49.3	49.1
16/8/2022 16:15	0:15:00	35	50.5	65.4	54.9	52.5	51.4	49.9	49.4	49.3	49.1
16/8/2022 16:30	0:15:00	36	60	81.7	71.3	66	62.9	52.2	49.4	49.2	48.6
16/8/2022 16:45	0:15:00	37	59.6	85	70.1	64.9	62	51.6	46.9	46.4	45.9
16/8/2022 17:00	0:15:00	38	52.2	74.5	62.3	56.4	54.2	47.8	46.1	45.9	45.5
16/8/2022 17:15	0:15:00	39	51.5	79.3	61.4	56	53.4	47.2	45.3	45.1	44.8
16/8/2022 17:30	0:15:00	40	50.1	71.8	60.3	53.8	51.3	47.1	45.3	45.1	44.8
16/8/2022 17:45	0:15:00	41	50.2	72.8	58.8	54.6	52.4	47.5	45.4	45.1	44.8
16/8/2022 18:00	0:15:00	42	50.7	73.2	59.4	54.8	52.9	47.8	45.2	44.9	44.6

16/8/2022 18:15	0:15:00	43	49.9	68	60.3	55.6	53.2	45.5	39.7	38.9	37.6
16/8/2022 18:30	0:15:00	44	52.7	74.3	64.5	59.2	55.2	45.5	39.8	38.9	37.6
16/8/2022 18:45	0:15:00	45	52	72.2	62.3	57.6	55.1	45.7	39.8	38.8	37.5
16/8/2022 19:00	0:15:00	46	55.1	71.8	64.2	60.9	58.8	51.6	43.8	41.9	39.3
16/8/2022 19:15	0:15:00	47	52.8	74.5	62.1	58	55.8	48.9	42.8	41.4	39.3
16/8/2022 19:30	0:15:00	48	51.7	77.4	62.3	57.5	54.7	45.2	38.5	37.8	37
16/8/2022 19:45	0:15:00	49	50.2	74.7	61	55.6	52.8	43.4	37.8	37.1	36.2
16/8/2022 20:00	0:15:00	50	53.1	78.1	63.5	58.8	55.7	44.9	38.6	37.9	36.9
16/8/2022 20:15	0:15:00	51	46.8	66.8	58.1	52.1	48.8	41.9	37.7	37.1	36.3
16/8/2022 20:30	0:15:00	52	49.1	73.8	60.7	54.1	50.6	40.9	37.5	36.9	36
16/8/2022 20:45	0:15:00	53	44	61.3	54.6	49.8	46.7	39.4	37.3	37	36.4
16/8/2022 21:00	0:15:00	54	49.3	72.5	59.6	55.1	52.5	43.4	38	37.4	36.7
16/8/2022 21:15	0:15:00	55	49.1	69	58.7	54.3	52	45.1	39.3	38.5	37.4
16/8/2022 21:30	0:15:00	56	46.6	70.2	57.5	51.3	48.2	41	36.9	36.4	35.7
16/8/2022 21:45	0:15:00	57	46.1	68.7	56.7	50.9	48.6	40.8	37.5	37	36.2
16/8/2022 22:00	0:15:00	58	46.3	70.9	58.2	51.1	47.9	40.2	37.3	36.8	36.2
16/8/2022 22:15	0:15:00	59	50.4	78.5	60.5	55.7	52.5	43	37.1	36.5	35.7
16/8/2022 22:30	0:15:00	60	52.3	78.1	64	56.8	53.3	45.8	44.3	44.1	38.5
16/8/2022 22:45	0:15:00	61	49.5	73.8	59	53.9	51.3	45.7	44.2	44	43.6
16/8/2022 23:00	0:15:00	62	47	65.3	55.6	51.5	49.1	44.7	43.8	43.6	43.3
16/8/2022 23:15	0:15:00	63	50	78.2	59.4	54.6	51.6	45.6	44.1	43.9	43.6
16/8/2022 23:30	0:15:00	64	47	69.4	57.1	50.9	48.2	44.1	43.3	43.2	42.9
16/8/2022 23:45	0:15:00	65	46.6	67.7	55.4	50.5	48.1	44	43.3	43.1	42.9
17/8/2022 0:00	0:15:00	66	45	67.8	50	46.7	45.6	43.9	43.3	43.2	42.9
17/8/2022 0:15	0:15:00	67	45.2	67	51.5	47.6	46.2	44.1	43.4	43.3	43
17/8/2022 0:30	0:15:00	68	46.7	76.9	55.7	47.1	45.4	43.9	43.4	43.2	43
17/8/2022 0:45	0:15:00	69	44.3	61.2	49.3	45.1	44.4	43.7	43.3	43.2	43
17/8/2022 1:00	0:15:00	70	45	75.6	47	44.7	44.4	43.7	43.3	43.1	42.9
17/8/2022 1:15	0:15:00	71	44.7	70.1	48.5	45.8	45	44	43.4	43.3	43.1
17/8/2022 1:30	0:15:00	72	46.2	69.7	55.1	48.9	46.5	44.1	43.5	43.4	43.1
17/8/2022 1:45	0:15:00	73	45	67	52.1	46.3	44.8	43.9	43.3	43.2	43
17/8/2022 2:00	0:15:00	74	44.3	59	49.1	45.5	44.6	43.7	43.3	43.1	42.9
17/8/2022 2:15	0:15:00	75	45.5	63.5	53.4	48.3	46.2	44.1	43.4	43.3	43.1
17/8/2022 2:30	0:15:00	76	45.6	62.5	52.8	48.9	46.9	44.3	43.7	43.6	43.3

17/8/2022 2:45	0:15:00	77	44.4	57.3	47.9	45.2	44.8	44.1	43.6	43.5	43.2
17/8/2022 3:00	0:15:00	78	44.8	59.2	50.6	46.9	45.6	44	43.5	43.4	43.2
17/8/2022 3:15	0:15:00	79	44.4	55.3	48.3	45.9	45.1	44	43.5	43.4	43.2
17/8/2022 3:30	0:15:00	80	45.6	55.5	52.7	49.4	47.3	44.3	43.7	43.6	43.3
17/8/2022 3:45	0:15:00	81	46.1	58.2	53.3	50.8	48.2	44.3	43.7	43.5	43.3
17/8/2022 4:00	0:15:00	82	45	58.9	50.2	47.3	45.9	44.3	43.7	43.5	43.3
17/8/2022 4:15	0:15:00	83	44.5	55.1	48.3	46.1	45.3	44.2	43.5	43.4	43.2
17/8/2022 4:30	0:15:00	84	45.7	65.8	50.3	46.5	45.2	44.1	43.5	43.4	43.1
17/8/2022 4:45	0:15:00	85	45.5	65.9	52.9	48.1	46.6	44.2	43.5	43.4	43.1
17/8/2022 5:00	0:15:00	86	45	64.3	50.5	47.3	46	44.1	43.5	43.3	43.1
17/8/2022 5:15	0:15:00	87	45.4	70.3	51.3	47.8	46.2	44	43.4	43.3	43
17/8/2022 5:30	0:15:00	88	44.8	63.2	51.5	46.4	45	43.8	43.3	43.2	42.9
17/8/2022 5:45	0:15:00	89	46.2	71	55	47.9	46.1	43.7	43.1	43	42.8
17/8/2022 6:00	0:15:00	90	46.8	67.8	56.1	50.8	48.4	44.1	43.3	43.2	42.9
17/8/2022 6:15	0:15:00	91	50	71.7	58.3	50.2	48.2	44.1	43.3	43.2	42.9
17/8/2022 6:30	0:15:00	92	52.4	71.3	68.4	51.4	48.7	44.4	43.5	43.3	43.1
17/8/2022 6:45	0:15:00	93	49	66.5	57.9	53.8	51.7	46.2	44	43.7	43.4
17/8/2022 7:00	0:15:00	94	51	69.9	61.7	55.7	53.2	47.2	44.5	44.1	43.6
17/8/2022 7:15	0:15:00	95	50	71.3	59	54.9	52.8	46.4	43.9	43.7	43.2
17/8/2022 7:30	0:15:00	96	52.2	71.8	63.2	55.6	52.6	46.1	43.7	43.5	43.1
17/8/2022 7:45	0:15:00	97	54.9	73.5	69.6	59.2	53.9	45.2	43.5	43.3	43
17/8/2022 8:00	0:15:00	98	54.1	74.7	66.6	59.8	55.7	45.6	43.5	43.3	43
17/8/2022 8:15	0:15:00	99	54.8	73.1	67.5	60.7	56.9	46.9	43.9	43.6	43.2
17/8/2022 8:30	0:15:00	100	54.2	86.1	63.5	57.7	54.5	46.2	44.2	43.8	43.4
17/8/2022 8:45	0:15:00	101	53.1	75.2	64.9	58.4	55.3	47	44.4	44	43.5
17/8/2022 9:00	0:15:00	102	52.9	77.9	63.3	58	55.4	46.9	44.5	44.2	43.7
17/8/2022 9:15	0:15:00	103	50.8	69.8	63.2	54.6	51.8	44.9	43.7	43.5	43.1
17/8/2022 9:30	0:15:00	104	53.2	76	63.7	58.6	55.9	47.8	44.8	44.5	44
17/8/2022 9:45	0:15:00	105	55.8	77.1	67.6	61.2	58.2	47.4	43.4	43.2	42.7
17/8/2022 10:00	0:15:00	106	58.9	81.8	70.2	64.9	61.9	51.3	44.5	43.7	43.1
17/8/2022 10:15	0:15:00	107	58.1	88.8	67.5	60.9	57.7	48.6	44.6	44.1	43.3
17/8/2022 10:30	0:15:00	108	51.2	76.4	62	56	52.6	46.4	44.8	44.6	44.2
17/8/2022 10:45	0:15:00	109	47.9	72.1	56.4	50.8	48.8	45.6	44.5	44.3	44
17/8/2022 11:00	0:15:00	110	56.8	86.8	67.5	62.2	58.7	48.2	42	40.2	38.1

17/8/2022 11:15	0:15:00	111	50.2	71.8	59.1	54.8	52.9	47.5	42.3	40.7	37.8
17/8/2022 11:30	0:15:00	112	51.8	77.3	60.8	56.1	54.1	47.7	42.8	41.4	39.3
17/8/2022 11:45	0:15:00	113	54.3	75.7	65.6	59.7	56.3	48.7	43	41.4	39.2
17/8/2022 12:00	0:15:00	114	53.7	71.1	64.8	59.9	57	47.8	42.3	41.3	39.2
17/8/2022 12:15	0:15:00	115	52.9	79.7	60.2	56.8	55.9	49.6	43.9	42.5	39.5
17/8/2022 12:30	0:15:00	116	53.7	72	62.9	58.9	57.1	49.6	43.3	41.9	39.4
17/8/2022 12:45	0:15:00	117	51	76.9	60.6	55.3	53.8	45.8	40.5	39.4	37.8
17/8/2022 13:00	0:15:00	118	48.8	68.2	58.9	52.7	50.9	45.3	40.7	39.5	37.9
17/8/2022 13:15	0:15:00	119	50.9	68.7	61.5	56.4	53.7	46.2	41	39.7	37.9
17/8/2022 13:30	0:15:00	120	49.5	71.7	58.2	53.9	51.9	46.2	41.9	40.6	38.9
17/8/2022 13:45	0:15:00	121	53.3	72.2	63.9	59.5	56.4	47.7	42.7	41.5	39.2
17/8/2022 14:00	0:15:00	122	49.3	68.4	58.3	54.4	52.3	46.2	41.9	40.5	38.3
17/8/2022 14:15	0:15:00	123	50.1	80	59.3	53.4	50.9	44.5	38.8	37.9	36.8
17/8/2022 14:30	0:15:00	124	54.4	74.8	65.4	61.2	58.2	46	38.6	37.8	36.7
17/8/2022 14:45	0:15:00	125	50.2	79.4	60.8	54.4	51.8	43.5	38.7	38	36.9
17/8/2022 15:00	0:15:00	126	52.1	73.2	62.3	57.8	55.3	47.1	40.5	39.4	38
17/8/2022 15:15	0:15:00	127	51.2	70.6	60.7	56.9	54.8	47.2	41	39.6	37.9
17/8/2022 15:30	0:15:00	128	51.3	69.9	61.8	56.8	54.5	46.5	39.9	38.8	37.5
17/8/2022 15:45	0:15:00	129	56.2	72.9	66.7	62.7	60.1	49.8	40.8	39.4	37.9
17/8/2022 16:00	0:15:00	130	51.4	75.1	61.8	56.7	54.3	46.4	39.2	38.3	37.2
17/8/2022 16:15	0:15:00	131	56.2	75.9	67	62.8	59.6	49.6	40.5	39.1	37.7
17/8/2022 16:30	0:15:00	132	49.8	72.4	61.2	56.1	53	41.4	37.8	37.3	36.6
17/8/2022 16:45	0:15:00	133	54.7	79.6	65.1	60.3	57.5	46.3	38.9	38.1	36.9
17/8/2022 17:00	0:15:00	134	56.9	83.5	67	61.7	58.7	47.6	39.7	38.5	36.5
17/8/2022 17:15	0:15:00	135	57	84.3	67.3	61.6	58	44.7	37.9	37	35.7
17/8/2022 17:30	0:15:00	136	54.3	81.7	65	59	54.8	44.1	39.8	39	37.9
17/8/2022 17:45	0:15:00	137	46.1	70	56.1	50.7	48	41.9	38.9	38.4	37.6
17/8/2022 18:00	0:15:00	138	54.8	79.8	66.4	59	55.4	46.7	40.7	39.6	38.4
17/8/2022 18:15	0:15:00	139	58	86.2	68.4	61.7	58.2	47.9	41.5	40.5	39
17/8/2022 18:30	0:15:00	140	55.3	76.2	66.2	60.9	57.8	49.5	43	41.7	39.8
17/8/2022 18:45	0:15:00	141	56.3	83.3	68	60.8	57.1	47.5	41.1	40	38.6
17/8/2022 19:00	0:15:00	142	47.7	65	56.8	52.5	50.5	45.1	40.7	39.7	38.5
17/8/2022 19:15	0:15:00	143	50	70.7	58.5	55.1	53.2	47.2	42.3	41.1	39.7
17/8/2022 19:30	0:15:00	144	46.3	62.3	55.8	52.1	49.9	42.4	38.4	37.9	37.2

17/8/2022 19:45	0:15:00	145	46.1	71.8	55.2	51.1	49.1	41.7	38.1	37.5	36.8
17/8/2022 20:00	0:15:00	146	43.9	66.3	53.6	48.9	46.4	39.9	37.9	37.4	36.7
17/8/2022 20:15	0:15:00	147	41.4	60.3	50.7	45.7	42.7	38.6	36.9	36.6	35.9
17/8/2022 20:30	0:15:00	148	37.8	54.9	42.1	39.7	38.9	37.1	36	35.8	35.4
17/8/2022 20:45	0:15:00	149	39.8	60.7	48.3	43.4	41.2	37.7	36.2	36	35.5
17/8/2022 21:00	0:15:00	150	44.7	67	53.1	49.5	47.8	41.8	37.6	36.9	36.1
17/8/2022 21:15	0:15:00	151	40	58.4	49.1	43.7	41.4	37.7	36.3	36.1	35.6
17/8/2022 21:30	0:15:00	152	41.4	59.7	50.5	46.3	44.1	38.6	36.6	36.2	35.8
17/8/2022 21:45	0:15:00	153	44	69.4	52.8	48.7	46.9	40.5	37.1	36.6	36
17/8/2022 22:00	0:15:00	154	48.3	74.9	60.2	52.8	49	39	36.4	36	35.5
17/8/2022 22:15	0:15:00	155	39.1	51.8	45.3	42.3	41	38	36.7	36.5	36.1
17/8/2022 22:30	0:15:00	156	39.9	63.9	47.1	43.2	41.6	37.8	36.4	36.1	35.8
17/8/2022 22:45	0:15:00	157	39.7	59.9	48.2	42.6	40.5	37.4	36.2	36	35.6
17/8/2022 23:00	0:15:00	158	39.7	63.9	49	42.3	40.6	37.4	36.3	36.1	35.7
17/8/2022 23:15	0:15:00	159	39.5	62.1	47.8	42.4	40.3	37.5	36.3	36.1	35.7
17/8/2022 23:30	0:15:00	160	39.8	59	47.8	42.7	40.9	38.3	36.6	36.3	35.6
17/8/2022 23:45	0:15:00	161	38.8	55.6	45.7	42.5	40.8	37.6	36.1	35.8	35.3
18/8/2022 0:00	0:15:00	162	40.5	62.3	48.5	44.9	42.9	38.1	36.3	36	35.6
18/8/2022 0:15	0:15:00	163	40.7	65.4	51.1	43.2	40.5	37.1	36	35.8	35.5
18/8/2022 0:30	0:15:00	164	37.4	54.1	41.7	39.2	38.5	36.9	35.9	35.7	35.4
18/8/2022 0:45	0:15:00	165	39.6	69.3	45.5	41.1	39.2	37	36	35.8	35.4
18/8/2022 1:00	0:15:00	166	39.4	70.8	42.6	38.8	38.2	36.8	35.9	35.7	35.3
18/8/2022 1:15	0:15:00	167	37.1	53.4	40	38.6	38	36.7	35.8	35.6	35.3
18/8/2022 1:30	0:15:00	168	41.2	74.5	43.2	39.2	38.4	36.8	35.8	35.6	35.3
18/8/2022 1:45	0:15:00	169	37.9	56.3	43	40.1	39.2	37.1	36	35.7	35.4
18/8/2022 2:00	0:15:00	170	38.5	57.3	45.4	40.9	39.7	37.3	36	35.7	35.3
18/8/2022 2:15	0:15:00	171	39	58	45.3	41.9	40.5	37.7	36.3	36	35.6
18/8/2022 2:30	0:15:00	172	39.4	53.6	46.5	43.1	41.5	38	36.3	36	35.5
18/8/2022 2:45	0:15:00	173	38.4	51.7	44	41	39.9	37.6	36.3	36	35.6
18/8/2022 3:00	0:15:00	174	38.8	55.8	46.5	42.8	40.6	37.1	35.9	35.7	35.3
18/8/2022 3:15	0:15:00	175	37.8	55	44.9	39.7	38.7	36.8	35.9	35.7	35.3
18/8/2022 3:30	0:15:00	176	37.3	52.6	40.4	38.9	38.3	36.9	35.9	35.7	35.3
18/8/2022 3:45	0:15:00	177	38.9	70.2	40.5	38.6	38.1	36.6	35.7	35.5	35.1
18/8/2022 4:00	0:15:00	178	38	61.4	46	39.5	38.4	36.7	35.7	35.6	35.2

18/8/2022 4:15	0:15:00	179	41.2	74.7	44.2	39.3	38.4	36.7	35.8	35.6	35.2
18/8/2022 4:30	0:15:00	180	37.7	50	43.3	39.9	38.8	36.9	35.9	35.7	35.3
18/8/2022 4:45	0:15:00	181	38.3	67.2	42.9	39.4	38.4	36.5	35.4	35.2	34.8
18/8/2022 5:00	0:15:00	182	39.8	62.6	47.9	43.3	41.5	38	36.3	36	35.5
18/8/2022 5:15	0:15:00	183	41.1	58.8	49.1	44.6	42.5	39.4	38.1	37.7	37
18/8/2022 5:30	0:15:00	184	39.2	57	45.6	41.9	40.6	37.9	36.4	36.1	35.6
18/8/2022 5:45	0:15:00	185	40.3	68.7	45.8	40.8	39.6	37.3	36.2	36	35.6
18/8/2022 6:00	0:15:00	186	38.3	59	43.4	40	39.1	37.1	36.1	35.8	35.4
18/8/2022 6:15	0:15:00	187	41.8	61.2	54.1	45.1	41.8	37.4	36.1	35.9	35.4
18/8/2022 6:30	0:15:00	188	39	58.8	47	42.3	40.5	37	35.8	35.5	35.2
18/8/2022 6:45	0:15:00	189	39.9	56.7	49.2	44.2	41.9	37.6	36	35.7	35.2
18/8/2022 7:00	0:15:00	190	46.7	62.9	55.9	52.1	50	43.2	38.6	37.8	36.8
18/8/2022 7:15	0:15:00	191	46	62.9	54.3	50.4	48.7	43.8	39.9	39.1	37.8
18/8/2022 7:30	0:15:00	192	47.2	66.9	57.3	52.7	50.1	43.1	38.6	38	37.2
18/8/2022 7:45	0:15:00	193	47.4	64.7	57	52.2	50.2	44.5	40.5	39.7	38.3
18/8/2022 8:00	0:15:00	194	47.3	66.8	57.1	52.1	49.8	43.7	40	39.3	38
18/8/2022 8:15	0:15:00	195	47.5	69.5	56.8	52.2	50.2	43.7	39.9	39.1	38
18/8/2022 8:30	0:15:00	196	48.8	63.4	56.7	54	52.3	46.2	41.1	40.1	38.6
18/8/2022 8:45	0:15:00	197	50.1	64.9	57.3	54.8	53.4	48.1	43.6	42.6	40.8
18/8/2022 9:00	0:15:00	198	50	73.6	58.8	55.1	53.2	46.3	40.4	39.3	37.8
18/8/2022 9:15	0:15:00	199	49.5	69.2	58.7	55.1	53	45.3	40.6	39.7	38.4
18/8/2022 9:30	0:15:00	200	46.6	68.4	57.4	51.7	49.1	42.1	38.3	37.8	37.2
18/8/2022 9:45	0:15:00	201	45.7	67.7	55.8	50.8	48.2	41.3	37.7	37.2	36.6
18/8/2022 10:00	0:15:00	202	48.5	75.2	56.9	52.4	50.4	44.2	40.8	39.9	38.4
18/8/2022 10:15	0:15:00	203	50.8	70.6	60.5	56	53.9	47.2	41.7	40.5	38.8
18/8/2022 10:30	0:15:00	204	49.1	68.6	57.8	54	52.3	46.3	41	39.9	38.5
18/8/2022 10:45	0:15:00	205	50.5	79.3	58.1	54	52.3	46.6	41.6	40.3	38.3
18/8/2022 11:00	0:15:00	206	46.8	65.7	56.9	51.8	49.3	43.1	39.3	38.6	37.5
18/8/2022 11:15	0:15:00	207	47.3	72	57.3	51.1	48.8	43.5	39.7	38.9	37.7
18/8/2022 11:30	0:15:00	208	47.5	75.6	56.2	52	49.8	43.1	39	38.2	37.1
18/8/2022 11:45	0:15:00	209	48.6	69.9	59.1	54	52.1	43.3	38.9	38.2	37.2
18/8/2022 12:00	0:15:00	210	47.1	70.7	57	51.7	49.2	41.9	38.7	38.1	37.2
18/8/2022 12:15	0:15:00	211	47.2	64.9	57.5	52.4	50.5	42.5	38.7	38.1	37.2
18/8/2022 12:30	0:15:00	212	46.9	69.5	57.8	52	49	41.4	38.4	38	37.3

18/8/2022 12:45	0:15:00	213	43.7	63.5	52.7	49	46.6	40.6	38.3	37.9	37.3
18/8/2022 13:00	0:15:00	214	44.6	67.3	54.6	48.5	44.5	40.2	38.5	38.2	37.5
18/8/2022 13:15	0:15:00	215	44.1	68.9	54.3	48.2	45.6	39.9	38.1	37.7	37.1
18/8/2022 13:30	0:15:00	216	44.1	64.2	53.9	48.9	46.5	40.4	38.2	37.8	37.3
18/8/2022 13:45	0:15:00	217	48.7	78.5	58.6	52.9	50.1	43.2	39.9	39.3	38.4
18/8/2022 14:00	0:15:00	218	44.4	64.7	54.7	49.6	46.6	40.7	38.3	37.9	37.2
18/8/2022 14:15	0:15:00	219	50	74.3	60.6	55.4	52.7	43.9	39.6	39	38.1
18/8/2022 14:30	0:15:00	220	48.1	69.4	59.6	53.9	51	42.1	39	38.5	37.7
18/8/2022 14:45	0:15:00	221	44.8	65.7	53.5	49.6	47.4	41.5	38.9	38.5	37.8
18/8/2022 15:00	0:15:00	222	43.1	59.4	51.1	47.2	45.4	41.1	39	38.6	37.8
18/8/2022 15:15	0:15:00	223	46.2	69.3	56.5	51.2	48.5	41.8	39.1	38.6	37.8
18/8/2022 15:30	0:15:00	224	44.4	66.6	54.6	48.3	46.2	40.7	38.5	38	37.3
18/8/2022 15:45	0:15:00	225	40.1	63	46.7	41.8	40.5	38.6	37.4	37.1	36.7
18/8/2022 16:00	0:15:00	226	43.8	63.2	54.7	49.6	45.9	39.4	37.8	37.4	36.9
18/8/2022 16:15	0:15:00	227	43.1	69.1	53.1	44.7	42.5	38.8	37.2	36.9	36.4
18/8/2022 16:30	0:15:00	228	44.3	75.7	52	46	43.7	39.8	37.8	37.4	36.7
18/8/2022 16:45	0:15:00	229	41.5	64.7	48.9	44	42.4	39.4	37.9	37.6	37
18/8/2022 17:00	0:15:00	230	46.7	69.7	58	50.8	47.8	40.8	38.5	38.1	37.5
18/8/2022 17:15	0:15:00	231	43.5	70.3	53.2	46.6	44	40.2	38.2	37.8	37.1
18/8/2022 17:30	0:15:00	232	41.9	60.4	50.6	45.7	43.4	39.8	38.1	37.8	37.1
18/8/2022 17:45	0:15:00	233	41.1	65.2	50.6	43	41.3	39.1	37.7	37.4	36.9
18/8/2022 18:00	0:15:00	234	40.7	63.5	46.4	42.2	41.3	39.3	37.9	37.5	37
18/8/2022 18:15	0:15:00	235	45.2	66.5	54.6	51.1	48.1	41.1	38.4	37.9	37.3
18/8/2022 18:30	0:15:00	236	46.2	64.7	56.7	51.7	49.1	41.9	38.9	38.4	37.5
18/8/2022 18:45	0:15:00	237	45.3	66.3	55.9	50.8	48	40.7	38.5	38.1	37.4
18/8/2022 19:00	0:15:00	238	44.4	63.6	53.4	48.5	46.1	41.7	39.4	38.9	38.1
18/8/2022 19:15	0:15:00	239	49.1	67.8	60.1	55.2	52.7	43.3	38.5	37.9	37.1
18/8/2022 19:30	0:15:00	240	42.7	59.8	51.5	47.5	45.5	40	37.6	37.2	36.6
18/8/2022 19:45	0:15:00	241	49.3	78	60.4	53.8	50.5	41.7	38.3	37.7	36.8
18/8/2022 20:00	0:15:00	242	46.5	74.1	57.5	50.2	47	39.8	37.3	36.9	36.1
18/8/2022 20:15	0:15:00	243	44	64.7	54.7	48.4	45.3	39.5	37.4	36.9	36.2
18/8/2022 20:30	0:15:00	244	43.4	69.6	52.8	47.3	44.3	39.4	37.2	36.8	36.2
18/8/2022 20:45	0:15:00	245	42.4	66.4	51.9	46.8	43.6	38.7	37	36.6	36.1
18/8/2022 21:00	0:15:00	246	45.6	76.6	53.7	45.4	42.5	38.2	36.7	36.4	35.9

18/8/2022 21:15	0:15:00	247	40	60.5	49.2	43.6	41	37.6	36.4	36.1	35.7
18/8/2022 21:30	0:15:00	248	40.8	73.1	47.2	41.7	39.8	37.5	36.3	36	35.5
18/8/2022 21:45	0:15:00	249	39.1	61.5	47.6	41.5	39.7	37.1	35.8	35.5	35.1
18/8/2022 22:00	0:15:00	250	40.7	68	48.5	44	41.4	37.3	35.9	35.7	35.2
18/8/2022 22:15	0:15:00	251	37.5	58.7	41.1	39.1	38.5	37	35.9	35.7	35.3
18/8/2022 22:30	0:15:00	252	37.2	53.5	40	38.8	38.3	36.8	35.8	35.5	35.1
18/8/2022 22:45	0:15:00	253	37.3	52.3	41	39	38.4	36.8	35.7	35.5	35.1
18/8/2022 23:00	0:15:00	254	39.8	64.6	48.4	42.4	40.3	37.5	36	35.7	35.2
18/8/2022 23:15	0:15:00	255	41.3	67.9	48.5	43.1	41.7	38.6	36.7	36.4	35.9
18/8/2022 23:30	0:15:00	256	41	66.5	50	43.7	41.8	38.5	36.7	36.4	35.8
18/8/2022 23:45	0:15:00	257	39.9	64.2	46.9	42.2	40.9	38.3	36.8	36.5	36
19/8/2022 0:00	0:15:00	258	39.8	56.6	47	43.5	41.8	38.4	36.7	36.4	35.9
19/8/2022 0:15	0:15:00	259	39	58.6	45.3	42.3	40.8	37.7	36.3	36	35.6
19/8/2022 0:30	0:15:00	260	38.9	57.9	44	41.7	40.6	37.9	36.4	36.1	35.6
19/8/2022 0:45	0:15:00	261	39.2	56.1	45.7	42.5	41.1	37.9	36.4	36.1	35.7
19/8/2022 1:00	0:15:00	262	39.1	58.4	44.6	41.4	40.5	38.2	36.7	36.4	35.9
19/8/2022 1:15	0:15:00	263	38.9	49.9	45.4	41.5	40.4	38	36.6	36.2	35.7
19/8/2022 1:30	0:15:00	264	39.2	58.9	44.9	42.2	40.9	38.1	36.4	36	35.5
19/8/2022 1:45	0:15:00	265	39	54.3	44.3	41.9	40.8	38.2	36.6	36.2	35.6
19/8/2022 2:00	0:15:00	266	40.1	66.1	46.3	41.8	40.6	38.2	36.7	36.3	35.8
19/8/2022 2:15	0:15:00	267	38.4	48.2	42.2	40.4	39.8	38	36.7	36.3	35.8
19/8/2022 2:30	0:15:00	268	38.6	53	44.2	40.4	39.7	38	36.7	36.4	35.8
19/8/2022 2:45	0:15:00	269	38.4	48.1	42.2	40.5	39.8	38	36.6	36.3	35.8
19/8/2022 3:00	0:15:00	270	39.1	55.9	44.6	41.4	40.4	38.3	36.8	36.5	36
19/8/2022 3:15	0:15:00	271	40.2	60.1	45.9	42.9	41.6	39.3	37.9	37.5	36.8
19/8/2022 3:30	0:15:00	272	39.3	51.3	44.2	41.8	40.9	38.7	37.1	36.7	36.1
19/8/2022 3:45	0:15:00	273	41.9	71.1	50	42.7	41.3	38.7	37	36.6	36
19/8/2022 4:00	0:15:00	274	38.4	55.2	42.7	40.5	39.7	37.8	36.5	36.2	35.7
19/8/2022 4:15	0:15:00	275	38.5	57.5	42.6	40.4	39.7	37.8	36.5	36.3	35.8
19/8/2022 4:30	0:15:00	276	38.5	63.5	41.8	39.9	39.2	37.6	36.5	36.2	35.7
19/8/2022 4:45	0:15:00	277	39	48.1	46.3	41.3	40.2	38.2	36.8	36.5	36
19/8/2022 5:00	0:15:00	278	38.9	56.3	46.3	41.7	40.1	37.8	36.6	36.3	35.9
19/8/2022 5:15	0:15:00	279	40	60.5	48.6	42.8	40.9	38.3	36.9	36.6	36.1
19/8/2022 5:30	0:15:00	280	39.3	64	45.1	41.2	40	37.8	36.4	36.1	35.6

19/8/2022 5:45	0:15:00	281	39.1	56.8	45.6	42.2	40.6	38	36.5	36.2	35.7
19/8/2022 6:00	0:15:00	282	39	57.1	43.9	41.3	40.4	38.3	36.9	36.6	36.1
19/8/2022 6:15	0:15:00	283	39.3	59.3	46.8	41.6	40.3	37.9	36.5	36.2	35.8
19/8/2022 6:30	0:15:00	284	38.3	53	42	40.3	39.6	37.8	36.5	36.3	35.8
19/8/2022 6:45	0:15:00	285	40.3	63.6	46.9	43	41.5	38.6	37	36.6	36
19/8/2022 7:00	0:15:00	286	42.2	60.4	50.8	46.3	44	40.1	38.1	37.6	36.8
19/8/2022 7:15	0:15:00	287	42.4	66.6	51.2	46.2	44.3	40	37.7	37.3	36.6
19/8/2022 7:30	0:15:00	288	52.5	80	63	56.9	52.7	41.4	37.9	37.4	36.6
19/8/2022 7:45	0:15:00	289	53.5	80.5	66.1	57.9	52.7	40.8	37.7	37.2	36.7
19/8/2022 8:00	0:15:00	290	41.4	62	50	45.5	43.5	39	37.2	36.9	36.4
19/8/2022 8:15	0:15:00	291	43	67.5	53	47.2	44.7	39.3	37.2	36.8	36.4
19/8/2022 8:30	0:15:00	292	43.1	66.4	52.4	47	44.8	39.7	37.6	37.2	36.6
19/8/2022 8:45	0:15:00	293	39.8	55.3	47.1	43.2	41.5	38.3	37	36.7	36.3
19/8/2022 9:00	0:15:00	294	39.6	57.8	46.5	42.8	41.2	38.1	36.8	36.6	36.2
19/8/2022 9:15	0:15:00	295	41.8	61	51.3	46.3	43.8	38.8	37.1	36.9	36.4
19/8/2022 9:30	0:15:00	296	43.3	63.9	54.5	48	45	39	37	36.6	36.2
19/8/2022 9:45	0:15:00	297	39.9	60.4	50.5	42.1	40.1	37.5	36.5	36.2	35.8
19/8/2022 10:00	0:15:00	298	41.7	64.2	49.3	45	43.3	39.8	37.3	37	36.4
19/8/2022 10:15	0:15:00	299	43.2	62.5	53.8	47.3	44.7	40.3	38.7	38.1	37
19/8/2022 10:30	0:15:00	300	42.9	67.4	53.6	47.3	44.5	38.7	37.1	36.8	36.4
19/8/2022 10:45	0:15:00	301	46.7	77.3	56	50.8	48.3	40.9	37.5	37.1	36.4
19/8/2022 11:00	0:15:00	302	43.2	61.5	51.5	47.7	45.8	40.7	37.9	37.3	36.6
19/8/2022 11:15	0:15:00	303	51	84.6	57.5	52.3	49.8	41.9	38.2	37.6	36.6
19/8/2022 11:30	0:15:00	304	51.8	73.1	64.5	57.6	53	43.2	38.6	37.9	37.1
19/8/2022 11:45	0:15:00	305	56.1	77.9	67.6	62.7	59.2	46.1	39.2	38.5	37.6
19/8/2022 12:00	0:15:00	306	61.8	88.3	73.3	67.3	63.7	51	41.2	39.8	38.1
19/8/2022 12:15	0:15:00	307	55.6	83.3	64.6	60.2	57.9	50.8	48	47.1	44.5
19/8/2022 12:30	0:15:00	308	60.5	79.6	69.6	66.4	64.4	55.6	50.2	49.8	49.1
19/8/2022 12:45	0:15:00	309	54.6	77.1	64.8	60.3	57.9	50	48.6	48.3	47.9
19/8/2022 13:00	0:15:00	310	57.7	82.3	67.3	62.9	60.3	51.3	48.7	48.5	48.2
19/8/2022 13:15	0:15:00	311	54.7	84.3	63.2	58.4	55.8	49.7	47.6	47.3	46.8
19/8/2022 13:30	0:15:00	312	57.4	76.1	67	62.7	60.4	53.9	49.3	48.2	47.2
19/8/2022 13:45	0:15:00	313	57.1	81.7	66.1	61.8	59.3	53.8	52.4	52.1	51.8
19/8/2022 14:00	0:15:00	314	59.7	80.1	69.6	65.5	63.3	54.8	52.3	52.1	51.7

19/8/2022 14:15	0:15:00	315	56.9	74.4	66.9	62.9	60.6	51.7	48.3	48	47.5
19/8/2022 14:30	0:15:00	316	58.8	79.8	69	64.6	62.3	53.6	48.2	47.8	47.2
19/8/2022 14:45	0:15:00	317	58.1	82.7	68.5	63.9	61.1	51.8	48.3	48	47.7
19/8/2022 15:00	0:15:00	318	54.3	74.5	66.1	58.8	55.5	49.7	48.4	48	47.4
19/8/2022 15:15	0:15:00	319	56.1	75.9	67.7	61.2	56.8	49.9	48.9	48.8	48.4
19/8/2022 15:30	0:15:00	320	57.3	75.3	68.3	63.5	60.6	50.8	48.9	48.8	48.5
19/8/2022 15:45	0:15:00	321	55.7	73.2	66.1	61.7	58.6	50.7	49.2	49.1	48.8
19/8/2022 16:00	0:15:00	322	58.3	76.7	69.8	64.8	61.5	50.8	49.3	49.1	48.8
19/8/2022 16:15	0:15:00	323	56.2	77.4	66.7	62	59.4	50.4	47.6	47.3	46.8
19/8/2022 16:30	0:15:00	324	49.7	68.8	58.6	53.4	50.6	47.6	47	46.9	46.6
19/8/2022 16:45	0:15:00	325	56.1	82	66.8	62.2	59.1	49	47.3	47.2	46.7
19/8/2022 17:00	0:15:00	326	60	88.1	72.1	64.1	60.9	50.4	48	47.9	47.6
19/8/2022 17:15	0:15:00	327	53.5	73.4	64.5	58.3	55.6	49.4	47	46.8	46.4
19/8/2022 17:30	0:15:00	328	52.5	68.4	61.7	57.2	55.1	49.5	48.6	48.4	48.2
19/8/2022 17:45	0:15:00	329	52.6	71.3	62.6	57.1	54.8	49.6	48.6	48.4	48.1
19/8/2022 18:00	0:15:00	330	52.8	76.5	62.9	57.6	54.8	49.1	47.3	47	46.5
19/8/2022 18:15	0:15:00	331	60.6	76.3	71.4	70.4	64.6	48.4	46.8	46.6	46.3
19/8/2022 18:30	0:15:00	332	52	73.1	62.2	54	51.8	47.2	46.4	46.2	46
19/8/2022 18:45	0:15:00	333	51	73.4	61.2	54.9	51.9	47	46.3	46.2	45.9
19/8/2022 19:00	0:15:00	334	55.2	74.5	64.7	60.2	58.2	51.3	47.4	47	46.5
19/8/2022 19:15	0:15:00	335	53.5	74.9	64.7	59.2	55.6	48	46.5	46.3	46
19/8/2022 19:30	0:15:00	336	54.6	72.8	65.9	61.1	58.1	48.1	47.1	47	46.7
19/8/2022 19:45	0:15:00	337	61.7	82.4	72.8	67.8	65	54.5	48.2	47.8	47.3
19/8/2022 20:00	0:15:00	338	56.5	75.7	67.7	62.7	59.6	50.2	47.4	47.2	46.8
19/8/2022 20:15	0:15:00	339	54.7	80.9	64.8	59.7	56.5	50.1	48.1	47.8	47.2
19/8/2022 20:30	0:15:00	340	52.5	70.2	61.7	56.4	54.1	50.5	48.8	48.5	48
19/8/2022 20:45	0:15:00	341	54.7	81.1	63.7	58	55.6	51.2	49.2	48.8	48.3
19/8/2022 21:00	0:15:00	342	58.4	78.6	68.7	64.3	61.7	53.5	49.5	49	48.2
19/8/2022 21:15	0:15:00	343	56.6	74	67.2	62.6	59.9	51.8	48.1	47.6	47.2
19/8/2022 21:30	0:15:00	344	58.6	73.7	68.5	65.2	63	52.1	47.7	47.4	47
19/8/2022 21:45	0:15:00	345	59.3	74.8	69.1	65.8	63.8	52	47.3	47.1	46.7
19/8/2022 22:00	0:15:00	346	57.7	73.2	67.7	63.9	61.6	52.5	47.6	47.3	46.8
19/8/2022 22:15	0:15:00	347	55.1	71.1	67.5	60.4	57.2	49.3	47.4	47.2	46.9
19/8/2022 22:30	0:15:00	348	52.5	69.5	63.4	58	54.1	48.2	47.3	47.2	46.9

19/8/2022 22:45	0:15:00	349	52.1	70.1	63.2	57.3	54	48	47.1	47	46.7
19/8/2022 23:00	0:15:00	350	55.8	75.2	67	62.9	59.4	48.1	47.2	47	46.7
19/8/2022 23:15	0:15:00	351	56.3	74.7	68	62.9	59.6	48.4	46.7	46.4	45.6
19/8/2022 23:30	0:15:00	352	51.6	72.6	63.8	54.9	50.8	47.2	46.3	46.1	45.8
19/8/2022 23:45	0:15:00	353	54.2	71.8	66.2	61.2	56.9	46.7	46	45.8	45.6
20/8/2022 0:00	0:15:00	354	53.1	70.1	65.8	59.8	52.9	46.7	46	45.9	45.6
20/8/2022 0:15	0:15:00	355	47.8	63.9	52.9	51.3	49.3	46.8	46.2	46.1	45.8
20/8/2022 0:30	0:15:00	356	47.4	66.8	49.6	47.7	47.4	46.7	46.2	46.1	45.9
20/8/2022 0:45	0:15:00	357	48	68.9	53.8	50.4	49	46.8	46.2	46	45.8
20/8/2022 1:00	0:15:00	358	49.3	70.1	58.6	51.8	49.7	47	46.4	46.2	45.9
20/8/2022 1:15	0:15:00	359	48.9	64.9	57.3	52.6	50.5	46.7	46.1	45.9	45.7
20/8/2022 1:30	0:15:00	360	48.4	74.4	56.4	49.9	48.4	46.6	46	45.8	45.6
20/8/2022 1:45	0:15:00	361	51.2	72.5	61.6	57.1	54.1	46.6	46	45.8	45.6
20/8/2022 2:00	0:15:00	362	47.5	62.5	51.7	48.8	48	47	46.5	46.4	46.2
20/8/2022 2:15	0:15:00	363	48	64.6	53.6	50.5	49.3	47.1	46.5	46.4	46.2
20/8/2022 2:30	0:15:00	364	47.3	62.2	52.3	48.6	47.7	46.7	46.3	46.2	45.9
20/8/2022 2:45	0:15:00	365	47.5	62.4	52.7	49.4	48.4	46.8	46.2	46.1	45.9
20/8/2022 3:00	0:15:00	366	47.1	60	51.9	47.9	47.4	46.7	46.3	46.1	45.9
20/8/2022 3:15	0:15:00	367	49.6	70.7	59.3	53.7	50.6	47	46.3	46.2	45.9
20/8/2022 3:30	0:15:00	368	47.2	63.8	52.3	48.6	47.6	46.7	46.3	46.2	45.9
20/8/2022 3:45	0:15:00	369	48.1	65.7	55	51.7	49.7	46.8	46.3	46.2	45.9
20/8/2022 4:00	0:15:00	370	54.8	70.3	64.8	61	58.9	48.8	46.3	46.2	45.9
20/8/2022 4:15	0:15:00	371	49.1	72.7	60.8	50.9	48	46.5	46	45.9	45.7
20/8/2022 4:30	0:15:00	372	47.2	57	51.5	48.6	47.5	46.8	46.4	46.2	46
20/8/2022 4:45	0:15:00	373	48.5	67.8	55.5	51.3	49.3	46.9	46.3	46.2	46
20/8/2022 5:00	0:15:00	374	48.2	68.5	55.7	50.7	48.9	46.9	46.4	46.2	46
20/8/2022 5:15	0:15:00	375	49.1	70.6	58.6	52.3	49.5	46.8	46.2	46.1	45.8
20/8/2022 5:30	0:15:00	376	48.5	65.8	55.4	51.5	50	47.2	46.5	46.3	46.1
20/8/2022 5:45	0:15:00	377	50.3	71.6	58.8	53.6	51.8	47.8	46.7	46.5	46.3
20/8/2022 6:00	0:15:00	378	52.5	74.1	64	57.8	54.2	47.3	46.5	46.4	46.1
20/8/2022 6:15	0:15:00	379	51	70.3	61.6	55.6	51.6	47.3	46.4	46.2	45.9
20/8/2022 6:30	0:15:00	380	60	88.8	69.3	64.9	62	51.3	47.2	46.8	46.3
20/8/2022 6:45	0:15:00	381	55	74.1	66.5	60.5	57.3	49.2	47	46.7	46.3
20/8/2022 7:00	0:15:00	382	54.6	79.2	63.3	59	57.1	51.7	47.7	47	46.2

20/8/2022 7:15	0:15:00	383	55	74.4	65.9	60.7	57.8	50.2	46.8	46.5	46
20/8/2022 7:30	0:15:00	384	57.3	75	67	63	60.8	52.7	47.6	47	46.3
20/8/2022 7:45	0:15:00	385	56	75.9	65.5	61.3	59.2	52	47.1	46.6	45.9
20/8/2022 8:00	0:15:00	386	55.5	75.5	65.6	61.2	58.7	50.9	47	46.5	45.8
20/8/2022 8:15	0:15:00	387	56.7	76.5	66.4	62.1	59.7	53	48.9	48.1	46.7
20/8/2022 8:30	0:15:00	388	55.5	79.5	62.4	59.7	57.8	52.5	49.4	48.5	46.5
20/8/2022 8:45	0:15:00	389	55.6	74.3	67	59.9	57.1	51.8	48.6	48	47
20/8/2022 9:00	0:15:00	390	53.3	71	61.3	58	56.4	50.9	46.6	45.8	45.3
20/8/2022 9:15	0:15:00	391	57.6	78.9	67.8	62.4	59.9	54	50.4	49.6	48.2
20/8/2022 9:30	0:15:00	392	59.6	79.2	70.4	65.9	63.2	52.7	47.9	47.1	45.9
20/8/2022 9:45	0:15:00	393	59.8	74.7	70.1	66.1	63.7	54.5	47.3	46	45.1
20/8/2022 10:00	0:15:00	394	52	68.1	60.7	57.7	55.2	48.2	45.7	45.5	45
20/8/2022 10:15	0:15:00	395	54	74.2	64.1	59.9	57.8	48.6	46.2	45.9	45.4
20/8/2022 10:30	0:15:00	396	55.2	77.1	65.4	61.1	58.9	49.4	46.3	45.9	45.4
20/8/2022 10:45	0:15:00	397	60.4	79.2	70	66.3	64	55.6	48.7	47.9	46.3
20/8/2022 11:00	0:15:00	398	58.5	74.5	67.6	64	62.1	55.2	46.8	45.5	44.7
20/8/2022 11:15	0:15:00	399	57.5	74.8	66.4	63	61.2	54.1	46.2	45.3	44.7
20/8/2022 11:30	0:15:00	400	57.5	77.8	67.1	63.3	61	53.2	46.6	45.8	45
20/8/2022 11:45	0:15:00	401	55.6	75.3	65.3	61.3	59	51.1	45.5	45.1	44.7
20/8/2022 12:00	0:15:00	402	58.6	80.1	68.6	64.9	62.4	53	47.6	46.7	45.1
20/8/2022 12:15	0:15:00	403	57.2	73.6	66.2	62.2	60.3	54.7	49.5	48.2	46
20/8/2022 12:30	0:15:00	404	58.7	77.8	71.1	64.6	61.2	52.3	45.6	45.3	44.8
20/8/2022 12:45	0:15:00	405	59.6	81.8	70.1	65.9	63.7	52.3	45.6	45.3	45
20/8/2022 13:00	0:15:00	406	57.1	76.1	68.5	63.9	60.5	50.2	46.6	46.1	45.6
20/8/2022 13:15	0:15:00	407	55.4	75.9	65.1	61.2	59.2	49.3	44	41	38.3
20/8/2022 13:30	0:15:00	408	55.5	80.1	66.5	60.7	57.9	47.9	45.1	42.2	38.9
20/8/2022 13:45	0:15:00	409	56.4	73.6	64.1	60.9	59.6	54.4	46.6	46	45.6
20/8/2022 14:00	0:15:00	410	54	74.8	64.1	59.3	56.9	49.8	46.2	45.9	45.6
20/8/2022 14:15	0:15:00	411	52.1	70.8	61.1	56.4	54.8	49.6	46.1	45.8	45.5
20/8/2022 14:30	0:15:00	412	54.2	70.1	63.2	58.7	56.4	51.8	48.6	47.8	46.5
20/8/2022 14:45	0:15:00	413	55.4	75.2	66.7	60.8	57.8	50.4	46.9	46.7	46.3
20/8/2022 15:00	0:15:00	414	54.1	81	65.8	57.4	54	47.8	46.5	46.2	44.7
20/8/2022 15:15	0:15:00	415	60.9	94.7	69.4	63	60.1	50.7	45.3	44.5	43.6
20/8/2022 15:30	0:15:00	416	56.5	77.9	68.6	61.9	58.2	47.1	41.6	40.6	39.2

20/8/2022 15:45	0:15:00	417	54.5	81.9	66.5	58.9	54.8	45.1	40.1	39.2	38.3
20/8/2022 16:00	0:15:00	418	51.4	71.6	61.4	56.3	54.6	47.7	40	39.3	38.6
20/8/2022 16:15	0:15:00	419	52.2	64.2	58.8	56.8	55.6	50.6	45	43.2	40.2
20/8/2022 16:30	0:15:00	420	53.4	80.8	59.5	56.7	55.7	51.3	46.6	45	41.6
20/8/2022 16:45	0:15:00	421	61.1	93.6	67.3	58	54.4	44	39.5	39	38.4
20/8/2022 17:00	0:15:00	422	54.3	78.3	65	59.8	57	48.5	43.6	41.1	38.7
20/8/2022 17:15	0:15:00	423	53.8	71.6	64.3	59.1	56.3	49.4	47.8	47.6	47.2
20/8/2022 17:30	0:15:00	424	52.1	75.8	63.3	54.4	51.9	48.5	47.3	47.1	46.8
20/8/2022 17:45	0:15:00	425	52.2	76.8	61.3	56.6	53.8	48.7	47.2	46.9	46.5
20/8/2022 18:00	0:15:00	426	51.9	68	60.8	57	54.6	49.1	47.4	47.1	46.6
20/8/2022 18:15	0:15:00	427	49.8	66.5	57.3	52.7	51.3	48.5	47.2	47	46.6
20/8/2022 18:30	0:15:00	428	56.4	79	66.8	62.5	59.9	50.4	47.1	46.7	46.1
20/8/2022 18:45	0:15:00	429	56	77.1	66.4	61.8	59.2	50.9	47.9	47.5	46.9
20/8/2022 19:00	0:15:00	430	57	73.6	66.5	63.4	61.5	50.5	47.7	47.4	46.9
20/8/2022 19:15	0:15:00	431	56.6	74.7	66.4	62.6	60.4	51.6	48.2	47.7	47.1
20/8/2022 19:30	0:15:00	432	58.3	81.4	69.5	64.8	61.9	49.9	47.2	46.8	46.3
20/8/2022 19:45	0:15:00	433	48.7	69.9	57.1	52.1	49.7	46.6	45.9	45.8	45.6
20/8/2022 20:00	0:15:00	434	49.7	75.3	59.1	52.9	50.3	46.7	46	45.8	45.6
20/8/2022 20:15	0:15:00	435	47.5	60.8	52.5	49.6	48.6	46.8	46.2	46.1	45.8
20/8/2022 20:30	0:15:00	436	53.9	72.4	64.7	60.3	57.6	47.3	46.2	46.1	45.8
20/8/2022 20:45	0:15:00	437	53.1	73.7	63.9	58.9	55.8	47	46.2	46	45.7
20/8/2022 21:00	0:15:00	438	48.2	68.4	54	49.7	48.8	47.2	46.5	46.4	46.2
20/8/2022 21:15	0:15:00	439	49.8	68.5	59.4	54.5	51.3	47.2	46.5	46.3	46
20/8/2022 21:30	0:15:00	440	50.2	71.5	60.6	54.6	50.3	47.1	46.5	46.4	46.1
20/8/2022 21:45	0:15:00	441	48.8	64.1	58	53.2	49.3	46.8	46.3	46.2	45.9
20/8/2022 22:00	0:15:00	442	47.2	55.4	50.3	48.8	47.9	46.8	46.3	46.2	46
20/8/2022 22:15	0:15:00	443	54.5	72.8	65.6	60.7	57.5	47.9	46.7	46.5	46.1
20/8/2022 22:30	0:15:00	444	55	76.1	65.8	60.7	57.7	48.4	46.7	46.5	46.2
20/8/2022 22:45	0:15:00	445	47.5	57.8	51	49.4	48.7	47	46.5	46.4	46.2
20/8/2022 23:00	0:15:00	446	47.8	61.9	52.1	49.6	48.9	47.2	46.6	46.4	46.2
20/8/2022 23:15	0:15:00	447	51.6	74.9	62.2	56.4	53.5	48	46.7	46.5	46.1
20/8/2022 23:30	0:15:00	448	57.1	76.9	68.9	62.3	59.8	50.3	47.7	47.4	46.9
20/8/2022 23:45	0:15:00	449	58.4	82.9	70.2	63.6	60.2	49.8	47.5	47.2	46.8
21/8/2022 0:00	0:15:00	450	49.6	74.8	57.8	51	49.4	48	47.2	47.1	46.7

21/8/2022 0:15	0:15:00	451	56.7	78	68.4	62.2	58.8	48.8	47.7	47.3	46.8
21/8/2022 0:30	0:15:00	452	48.5	72.8	55.4	49.7	48.5	47.2	46.6	46.5	46.2
21/8/2022 0:45	0:15:00	453	47.8	68.4	52.1	48.7	48	47.1	46.6	46.4	46.2
21/8/2022 1:00	0:15:00	454	47.3	69	49.6	47.9	47.5	46.9	46.5	46.4	46.2
21/8/2022 1:15	0:15:00	455	47	59.3	49.2	47.6	47.4	46.8	46.4	46.3	46.1
21/8/2022 1:30	0:15:00	456	47.6	64.1	52.1	48.2	47.6	46.9	46.4	46.3	46.1
21/8/2022 1:45	0:15:00	457	47.3	71.1	49.3	47.8	47.5	46.9	46.4	46.3	46.1
21/8/2022 2:00	0:15:00	458	47	58.9	49.1	47.8	47.4	46.8	46.4	46.3	46.1
21/8/2022 2:15	0:15:00	459	47.1	62.8	49.3	48	47.6	46.9	46.4	46.3	46.1
21/8/2022 2:30	0:15:00	460	47	54.2	48.6	47.7	47.4	46.9	46.5	46.4	46.2
21/8/2022 2:45	0:15:00	461	47	54.1	49.1	47.9	47.5	46.9	46.4	46.3	46.1
21/8/2022 3:00	0:15:00	462	47.2	54.1	49.5	48.3	47.8	46.9	46.5	46.4	46.2
21/8/2022 3:15	0:15:00	463	47.2	57.3	49.5	48.1	47.6	47	46.5	46.4	46.2
21/8/2022 3:30	0:15:00	464	47.3	62.7	49.7	48.3	47.8	47	46.6	46.4	46.3
21/8/2022 3:45	0:15:00	465	47.6	59.7	50.5	48.8	48.3	47.2	46.7	46.6	46.3
21/8/2022 4:00	0:15:00	466	49.2	65.7	58	52.7	49.9	47.3	46.7	46.6	46.3
21/8/2022 4:15	0:15:00	467	47.4	54.5	49.4	48.3	47.9	47.2	46.7	46.6	46.4
21/8/2022 4:30	0:15:00	468	47.6	65	54.3	49.5	47.9	46.7	46.1	46	45.8
21/8/2022 4:45	0:15:00	469	47.5	64.4	53.3	49.1	48.1	46.7	46.1	46	45.7
21/8/2022 5:00	0:15:00	470	48.3	69.6	55.7	50.3	48.7	46.9	46.2	46.1	45.8
21/8/2022 5:15	0:15:00	471	50.8	66.9	60	55.6	53.3	48.2	46.8	46.6	46.2
21/8/2022 5:30	0:15:00	472	49.2	67.9	58.4	53.8	50.4	46.7	45.8	45.7	45.4
21/8/2022 5:45	0:15:00	473	48.2	70.8	56	50.1	48.6	46.4	45.8	45.7	45.4
21/8/2022 6:00	0:15:00	474	56.7	81.9	67.1	62.8	59.8	47.7	46	45.8	45
21/8/2022 6:15	0:15:00	475	54.6	74.2	65.9	61.2	57.6	48.1	46.9	46.4	44.7
21/8/2022 6:30	0:15:00	476	48.1	58.6	51.3	50	49.3	47.7	47.1	47	46.7
21/8/2022 6:45	0:15:00	477	49.9	68.1	59.3	54.2	50.5	47.7	47	46.9	46.6
21/8/2022 7:00	0:15:00	478	51	66.2	59.4	55.6	53.5	48.7	47.4	47.2	46.8
21/8/2022 7:15	0:15:00	479	50.7	70.9	59.8	54.9	52.3	48.3	47.4	47.3	47
21/8/2022 7:30	0:15:00	480	49.7	74.3	57	51.9	50.1	48.1	47.4	47.2	46.9
21/8/2022 7:45	0:15:00	481	52.1	82	59.1	50.8	49.5	48	47.3	47.2	46.9
21/8/2022 8:00	0:15:00	482	51.5	76.9	62.7	53.6	50.4	47.9	47.2	47.1	46.8
21/8/2022 8:15	0:15:00	483	49.2	70.2	56	51.3	49.8	48	47.2	47	46.7
21/8/2022 8:30	0:15:00	484	51.2	72.3	61.1	55.9	52.9	48.2	47.4	47.2	46.9

21/8/2022 8:45	0:15:00	485	53	70.3	63	58.4	56	48.8	47.6	47.4	47.1
21/8/2022 9:00	0:15:00	486	50.1	67.6	59.4	52.9	50.1	48.2	47.5	47.3	47
21/8/2022 9:15	0:15:00	487	49	69.3	55.9	50.2	49.1	47.9	47.3	47.1	46.9
21/8/2022 9:30	0:15:00	488	54.4	81.8	66.5	58	53	45.3	44.3	44.1	43.8
21/8/2022 9:45	0:15:00	489	53.3	81.4	62.5	50.5	48.1	45.4	44.8	44.6	44.4
21/8/2022 10:00	0:15:00	490	46.3	61	51.8	48.4	47.5	45.4	44.8	44.7	44.5
21/8/2022 10:15	0:15:00	491	57.1	78.1	69	63.3	59.8	48.4	45.2	45	44.7
21/8/2022 10:30	0:15:00	492	53.5	68.8	62.6	58.9	57	50	45.2	44.9	44.7
21/8/2022 10:45	0:15:00	493	58.5	80.4	70.1	63.9	60.7	52.4	45.5	44.9	41.9
21/8/2022 11:00	0:15:00	494	58.8	79.1	70.8	65.3	62.2	48.2	41.6	40.4	38.8
21/8/2022 11:15	0:15:00	495	51.2	78.9	60.9	56.8	54.3	44.6	39.8	38.9	37.8
21/8/2022 11:30	0:15:00	496	45.1	67.3	55	49.2	46.8	41.3	38	37.4	36.6
21/8/2022 11:45	0:15:00	497	44.2	67.7	53.1	49.2	46.7	41	38.4	37.9	37.2
21/8/2022 12:00	0:15:00	498	52.4	71.7	64.6	59.2	55.4	43.6	39.6	39	38.2
21/8/2022 12:15	0:15:00	499	49.5	70.4	59.5	55.1	52.7	44.1	41.4	41.2	40.8
21/8/2022 12:30	0:15:00	500	51.7	74	62.3	56.8	53.9	45.5	41.8	41.5	41.1
21/8/2022 12:45	0:15:00	501	48.7	67.5	57.9	54.2	52.1	44.5	41.4	41.2	40.9
21/8/2022 13:00	0:15:00	502	46.9	66.4	55.6	51.9	50.1	43.7	41.1	40.9	40.5
21/8/2022 13:15	0:15:00	503	49.3	69.6	58.9	54.4	52.3	45	40.1	39.4	38.7
21/8/2022 13:30	0:15:00	504	47.5	73.3	57.2	52.5	50	42.3	39	38.4	37.6
21/8/2022 13:45	0:15:00	505	44.6	64	55.8	48.8	46.1	40.2	38.2	37.8	37.4
21/8/2022 14:00	0:15:00	506	50.9	74.9	62.6	55.6	52.7	41.7	38.4	38	37.4
21/8/2022 14:15	0:15:00	507	57.3	91.5	65.4	59.5	56.6	45.7	38.5	37.5	36.4
21/8/2022 14:30	0:15:00	508	48.9	73.3	59.6	54.9	52.1	41.8	37	36.5	36
21/8/2022 14:45	0:15:00	509	53.1	71.4	65.1	59.9	56.7	42.9	37.6	37.1	36.4
21/8/2022 15:00	0:15:00	510	51.8	69.9	62.8	58.1	55.6	43.2	37.5	37	36.4
21/8/2022 15:15	0:15:00	511	51.5	73	62.3	57.6	55.1	43.9	38.6	38.1	37.3
21/8/2022 15:30	0:15:00	512	49.3	73.6	59.2	55	52.6	42.9	38.8	38.4	37.8
21/8/2022 15:45	0:15:00	513	47.3	65.5	58.2	53.7	50.8	40.8	38.7	38.3	37.4
21/8/2022 16:00	0:15:00	514	48.7	77.1	57.6	51.7	48.8	41.6	38.5	37.9	37.2
21/8/2022 16:15	0:15:00	515	52.7	69.3	62.4	58.5	56.5	48.3	41.7	40.7	39.3
21/8/2022 16:30	0:15:00	516	52.4	79.5	62.5	58.1	55.3	43.8	37.6	37.1	36.5
21/8/2022 16:45	0:15:00	517	46	66.1	57	50.9	48.3	40.4	37.8	37.4	36.8
21/8/2022 17:00	0:15:00	518	51	72.6	61.9	56.6	53.7	45.4	40	39.2	38.2

21/8/2022 17:15	0:15:00	519	49	73.5	59.8	54.2	51.3	43.8	39.6	39.1	38.5
21/8/2022 17:30	0:15:00	520	50.9	78.7	61.2	55.3	52.4	44.6	39.5	38.9	38.2
21/8/2022 17:45	0:15:00	521	50	71.6	60.3	56	53.4	44.4	39.1	38.7	38.1
21/8/2022 18:00	0:15:00	522	51	74.6	61.1	56.8	54.2	44.4	39.1	38.4	37.3
21/8/2022 18:15	0:15:00	523	45.4	66.8	57	48.7	46.2	39.6	37.6	37.3	36.8
21/8/2022 18:30	0:15:00	524	52	72.2	62.7	58.8	56.2	43.3	38.3	37.9	37.3
21/8/2022 18:45	0:15:00	525	52.7	71.5	64.3	59.2	56.3	45.5	39.4	38.5	37.6
21/8/2022 19:00	0:15:00	526	51.1	69.4	62.3	57.6	53.9	45.4	40.2	39.3	38.2
21/8/2022 19:15	0:15:00	527	51.1	73.3	64.4	56.4	50.4	42	39.4	38.9	38.1
21/8/2022 19:30	0:15:00	528	50.3	69.1	61.8	57.2	54	43	39.1	38.4	37.6
21/8/2022 19:45	0:15:00	529	53.4	76.3	65.2	58.8	55.7	44.5	39.4	38.9	38.2
21/8/2022 20:00	0:15:00	530	52.1	75.2	65.2	57.9	52.5	40.3	37.8	37.4	37
21/8/2022 20:15	0:15:00	531	56.8	82.9	67.6	62.4	59.9	48.6	39.5	38.3	37.2
21/8/2022 20:30	0:15:00	532	53.9	83.4	63.2	58.4	55.5	42.9	37.1	36.6	36
21/8/2022 20:45	0:15:00	533	45.4	70.3	59.1	47.1	44.4	37.5	36	35.7	35.2
21/8/2022 21:00	0:15:00	534	45.8	66.2	57.7	51.2	47.9	38	35.9	35.6	35.2
21/8/2022 21:15	0:15:00	535	55.2	75.4	66.4	61.5	59.1	46.3	37.8	37.1	36.2
21/8/2022 21:30	0:15:00	536	48.1	66.3	58.4	54.2	51.7	42.8	38.7	38.1	37.3
21/8/2022 21:45	0:15:00	537	44.6	62.7	55.5	50	47	40.3	38.1	37.8	37.1
21/8/2022 22:00	0:15:00	538	44.1	63.6	55.3	49.5	45.9	39.1	37.4	37.2	36.8
21/8/2022 22:15	0:15:00	539	40.8	61	49.4	43.9	42.1	38.7	37.5	37.3	37
21/8/2022 22:30	0:15:00	540	40.9	69.8	47.2	42.8	41.2	38.7	37.5	37.3	37
21/8/2022 22:45	0:15:00	541	46.7	71.9	57.6	51.9	49.1	39.7	37.4	37.2	36.8
21/8/2022 23:00	0:15:00	542	45.3	62.5	55.4	50.7	48.4	41	38	37.6	37.1
21/8/2022 23:15	0:15:00	543	45.4	66.5	55.7	50.9	48.3	39.9	37.6	37.2	36.8
21/8/2022 23:30	0:15:00	544	51.8	72.4	63.4	58	54.9	43.8	37.8	37.5	37
21/8/2022 23:45	0:15:00	545	53	81.3	64.2	56.9	52.8	42.2	37.7	37.2	36.5
22/8/2022 0:00	0:15:00	546	46.2	68.4	57.8	51.6	47.4	38.7	35.8	35.5	35
22/8/2022 0:15	0:15:00	547	43.3	68.3	51.4	46	44.6	41.3	36.5	36.1	35.6
22/8/2022 0:30	0:15:00	548	41.1	65.4	50.7	45.4	42.9	37.6	36.2	36	35.6
22/8/2022 0:45	0:15:00	549	44.4	66.2	55.1	49.7	46.8	38.7	36.7	36.4	35.9
22/8/2022 1:00	0:15:00	550	40	60.2	48	43.8	42	37.9	36.8	36.6	36.3
22/8/2022 1:15	0:15:00	551	40.7	59.8	48.4	44.6	43	38.4	36.8	36.7	36.3
22/8/2022 1:30	0:15:00	552	43.4	72.2	53.9	45.5	42	37.8	36.8	36.6	36.3

22/8/2022 1:45	0:15:00	553	47.8	67.8	59.7	54.6	51	38.7	37.3	37	36.6
22/8/2022 2:00	0:15:00	554	47.3	70.2	59.4	54.3	50	38.2	37	36.8	36.5
22/8/2022 2:15	0:15:00	555	41.2	62.7	50.1	45.5	43.2	38.4	37	36.7	36
22/8/2022 2:30	0:15:00	556	43.7	66.3	55.3	48.7	45	38.4	36.8	36.5	36.1
22/8/2022 2:45	0:15:00	557	40.5	60.6	48.5	45	42.6	38.2	36.9	36.6	36.3
22/8/2022 3:00	0:15:00	558	44.2	73.1	53.1	46.3	44.1	38.8	37	36.8	36.4
22/8/2022 3:15	0:15:00	559	45.2	68.6	57.6	48.7	44.4	38.4	37.1	36.9	36.6
22/8/2022 3:30	0:15:00	560	44.2	67.7	57.5	44.7	41.8	38.1	37	36.8	36.4
22/8/2022 3:45	0:15:00	561	43.3	68.7	55.4	46.7	42.6	38.1	37	36.8	36.5
22/8/2022 4:00	0:15:00	562	46.1	75	57.2	48.5	44.5	38.6	37.2	36.9	36.5
22/8/2022 4:15	0:15:00	563	41.7	67.2	50.8	45	43	38.7	37.2	37	36.6
22/8/2022 4:30	0:15:00	564	44	66.9	54.7	49.3	46.5	39	37.1	36.8	36.4
22/8/2022 4:45	0:15:00	565	41.4	63	49.4	45.5	44.5	38.6	37.1	36.8	36.5
22/8/2022 5:00	0:15:00	566	43.1	65.6	52.2	47.3	45.8	39.4	37.5	37.3	36.9
22/8/2022 5:15	0:15:00	567	47.3	72.6	58.3	52.6	49.2	40.9	37.9	37.5	36.9
22/8/2022 5:30	0:15:00	568	54.1	76.6	64.7	60	57.7	47	38.3	37.3	36
22/8/2022 5:45	0:15:00	569	56.6	84.9	66.2	59.1	55.5	42.8	37.7	37.4	36.8
22/8/2022 6:00	0:15:00	570	48	68.3	59.4	54.6	51.3	40.1	37.4	37.1	36.6
22/8/2022 6:15	0:15:00	571	44	67.6	55.4	47.1	43.9	39.1	38.2	38	37.7
22/8/2022 6:30	0:15:00	572	50.8	71.2	64.2	57.4	51.9	39.6	38.1	37.9	37.5
22/8/2022 6:45	0:15:00	573	51.4	73	63.1	57.8	54.3	41.6	37.4	36.9	36.1
22/8/2022 7:00	0:15:00	574	50.4	72.8	60.6	54.5	52.1	45.6	39.6	38.6	37.5
22/8/2022 7:15	0:15:00	575	47.9	67.3	56.7	52.9	51.1	45	39.7	38.9	37.8
22/8/2022 7:30	0:15:00	576	55.7	77.8	67	61.8	58.9	48.7	41.2	40	38.7
22/8/2022 7:45	0:15:00	577	52.8	73.2	65.1	59.3	55.4	41.6	38	37.6	37.2
22/8/2022 8:00	0:15:00	578	46.8	69.2	58.5	52.6	49.1	40.3	38.1	37.8	37.3
22/8/2022 8:15	0:15:00	579	45.1	66.7	56.5	49.2	45.8	39.4	37.9	37.6	37.3
22/8/2022 8:30	0:15:00	580	52.1	77.5	64.3	57.8	54.8	42.9	38.6	38.2	37.6
22/8/2022 8:45	0:15:00	581	50.6	76.6	62.3	54.1	50.4	40.9	38.3	38	37.4
22/8/2022 9:00	0:15:00	582	48.2	74.3	58	51.6	48.4	41.2	38.6	38.2	37.6
22/8/2022 9:15	0:15:00	583	52.7	75.7	65.3	58.3	53.8	42.1	39	38.5	37.8
22/8/2022 9:30	0:15:00	584	55.2	74.3	66.5	62.3	59.6	41.5	38.6	38.2	37.6
22/8/2022 9:45	0:15:00	585	54.5	79.3	66.3	60.8	56.8	42	38.6	38.2	37.6
22/8/2022 10:00	0:15:00	586	55.1	74.1	67	62.4	59	43.2	38.7	38.2	37.6

22/8/2022 10:15	0:15:00	587	62.9	84.5	73	69	66.7	57.4	47.4	45.1	40.5
22/8/2022 10:30	0:15:00	588	58.5	77.4	69.9	65.2	62.2	48.6	41.4	40.2	38.7
22/8/2022 10:45	0:15:00	589	61	77.4	71.9	67.8	65.1	53.2	41.9	41	40.1
22/8/2022 11:00	0:15:00	590	55.1	74.6	67.4	61.2	57.6	46	40.2	39.5	38.7
22/8/2022 11:15	0:15:00	591	51.3	73.5	63.4	56.8	53.2	43.2	39	38.3	37.4
22/8/2022 11:30	0:15:00	592	43.5	64.7	53.9	48.1	45.5	39.3	37.3	36.9	36.4
22/8/2022 11:45	0:15:00	593	46.8	66.9	57	52.1	49.6	42.5	38.4	37.8	37
22/8/2022 12:00	0:15:00	594	51.4	80.9	62.9	54.7	50.7	40.7	37.5	37	36.4
22/8/2022 12:15	0:15:00	595	47.1	72.4	59.4	48.6	44.2	38.9	37.2	36.8	36.3
22/8/2022 12:30	0:15:00	596	50.9	76.1	63.2	54.2	48	39.3	37.2	36.9	36.4
22/8/2022 12:45	0:15:00	597	49.2	70.2	59.8	54.2	51.1	44	39.3	38.4	37.3
22/8/2022 13:00	0:15:00	598	46	68.2	56.5	51.7	48.6	41.4	38.9	38.5	37.9
22/8/2022 13:15	0:15:00	599	44.7	69.2	55.7	48.9	45.1	40.2	38.3	37.9	37.3
22/8/2022 13:30	0:15:00	600	43.4	64.4	52.4	48.5	46	40.2	38.3	38	37.5
22/8/2022 13:45	0:15:00	601	48.1	78.8	55.4	49.4	45.8	39.9	38.2	37.8	37.3
22/8/2022 14:00	0:15:00	602	49.7	71.4	61.5	54	50.7	43.6	38.9	38.3	37.5
22/8/2022 14:15	0:15:00	603	46	68.7	56.5	51.3	48.7	41	37.9	37.5	36.9
22/8/2022 14:30	0:15:00	604	46.5	66.9	56.5	52	49.7	41.7	38.3	37.8	37.2
22/8/2022 14:45	0:15:00	605	46.2	69.3	57.4	50.6	47.5	39.9	37.4	37	36.2
22/8/2022 15:00	0:15:00	606	47	73.4	55.9	51.2	48.9	42	38.3	37.7	36.6
22/8/2022 15:15	0:15:00	607	53.7	73.2	69.5	55.6	50.9	41.6	37.7	37.2	36.6
22/8/2022 15:30	0:15:00	608	50.5	77.3	62.8	49.1	45.4	39.9	37.6	37.2	36.6
22/8/2022 15:45	0:15:00	609	55.9	78.5	67.9	62	58.5	44.1	38.2	37.6	36.9
22/8/2022 16:00	0:15:00	610	48.4	65.2	61.6	53.9	49.8	41.5	37.9	37.5	36.9
22/8/2022 16:15	0:15:00	611	42.6	61.8	51.1	47.9	45.4	39.2	37.2	36.8	36.3
22/8/2022 16:30	0:15:00	612	44.5	64.9	54.4	49.6	47.1	40.6	38.1	37.7	37
22/8/2022 16:45	0:15:00	613	45.2	61.4	54.2	50.2	48.4	42.6	38.5	38	37.4
22/8/2022 17:00	0:15:00	614	48.7	68.3	60.4	54.1	51.2	43	38.9	38.4	37.7
22/8/2022 17:15	0:15:00	615	49	70.3	59.2	54	51.5	44.8	40.3	39.7	38.9
22/8/2022 17:30	0:15:00	616	48.7	72.1	60.8	53.3	50.7	43.3	39.9	39.4	38.9
22/8/2022 17:45	0:15:00	617	46	69.9	57.5	50.5	47	40.5	38.9	38.6	38.2
22/8/2022 18:00	0:15:00	618	44.6	65.9	55.6	48.4	45.2	40.6	39	38.7	38.2
22/8/2022 18:15	0:15:00	619	52.5	78.1	64.8	57.6	53.3	43.8	40	39.5	39
22/8/2022 18:30	0:15:00	620	44.6	63.9	54.5	49.4	47.2	41	39.6	39.3	38.9

22/8/2022 18:45	0:15:00	621	44.7	69.9	53.6	48.8	46.1	40.7	39.4	39.2	38.8
22/8/2022 19:00	0:15:00	622	54.2	76.6	66.4	58.6	55.3	46.5	41	40.4	39.6
22/8/2022 19:15	0:15:00	623	52.9	72	64.9	58.3	54.9	46.7	40.9	40.3	39.7
22/8/2022 19:30	0:15:00	624	47.1	71.4	56.4	50.9	49.5	43.7	40.3	39.9	39.3
22/8/2022 19:45	0:15:00	625	43.8	63.5	54.2	47	44.7	40.9	39.6	39.4	39
22/8/2022 20:00	0:15:00	626	47.3	67.6	60.6	51.4	46.2	40.9	39.5	39.3	39
22/8/2022 20:15	0:15:00	627	49.6	71.3	61.1	54.8	51.2	43.3	39.1	38.4	37.5
22/8/2022 20:30	0:15:00	628	49	75.3	61.2	52.8	48.3	39.2	36.3	35.7	34.9
22/8/2022 20:45	0:15:00	629	45.8	67.4	58.2	50.4	46.4	40.9	38.4	37.7	36.7
22/8/2022 21:00	0:15:00	630	47.8	68.4	59.4	53.2	49.6	41.3	38.2	37.7	37
22/8/2022 21:15	0:15:00	631	45.4	67.7	58.6	47.2	43.4	40.4	38.3	37.9	37.3
22/8/2022 21:30	0:15:00	632	50.4	75.1	62.4	56	52.5	41.7	38.3	37.8	37.1
22/8/2022 21:45	0:15:00	633	43.6	63.4	53.7	48.7	46.1	39.8	37.8	37.4	36.9
22/8/2022 22:00	0:15:00	634	41.1	63.3	48.5	44.5	42.8	39.3	37.4	37	36.5
22/8/2022 22:15	0:15:00	635	48.9	70.3	63.6	47.8	44.2	39.8	37.7	37.3	36.7
22/8/2022 22:30	0:15:00	636	46.9	67.2	58	51.7	48.6	42.4	39.2	38.7	38
22/8/2022 22:45	0:15:00	637	41.7	68.3	49.4	44.5	42.6	39.2	38	37.8	37.4
22/8/2022 23:00	0:15:00	638	43.8	63.5	51.5	47.6	47	41	38.3	37.9	37.4
22/8/2022 23:15	0:15:00	639	43.2	61.5	52.8	47.9	45.1	40	38.3	38	37.6
22/8/2022 23:30	0:15:00	640	40	58.6	45.1	41.8	40.8	39.1	38	37.7	37.3
22/8/2022 23:45	0:15:00	641	40.1	59.9	45.5	42.5	41.3	39.1	37.9	37.7	37.3
23/8/2022 0:00	0:15:00	642	46.6	70.6	58	51.9	48.1	40.3	36.9	36.1	34.9
23/8/2022 0:15	0:15:00	643	43.2	63.4	55.1	46.5	43.3	38.5	36.7	36.3	35.4
23/8/2022 0:30	0:15:00	644	39.6	58.9	46.4	42.7	41.3	38.3	37.1	36.8	36.4
23/8/2022 0:45	0:15:00	645	43.1	68.2	48.9	45.4	44.6	40.5	38	37.6	36.9
23/8/2022 1:00	0:15:00	646	47.2	63.8	57.9	53.5	50.3	42.3	38.7	38.3	37.6
23/8/2022 1:15	0:15:00	647	43.3	65	54	47.3	44.3	39.6	38	37.7	37.2
23/8/2022 1:30	0:15:00	648	50.7	86.5	51.8	46.8	44.4	39.8	37.9	37.6	37.1
23/8/2022 1:45	0:15:00	649	44.1	73.5	53	47.8	44.7	39.5	37.9	37.6	37.2
23/8/2022 2:00	0:15:00	650	45.8	73.3	57	49.7	46.5	39.6	37.7	37.2	36.2
23/8/2022 2:15	0:15:00	651	42	64.8	50.5	44.8	43	39	37.4	37.1	36.5
23/8/2022 2:30	0:15:00	652	45.5	73	54.3	48.4	45.8	39.5	37.9	37.6	37.1
23/8/2022 2:45	0:15:00	653	48.8	70.8	61.4	53.2	48.3	39.8	38	37.7	37.2
23/8/2022 3:00	0:15:00	654	51.8	78.3	61.8	54.4	50.3	39.9	37.8	37.5	37.1

23/8/2022 3:15	0:15:00	655	56.8	87.6	66.4	57.4	53.7	43.1	38.9	38.4	37.7
23/8/2022 3:30	0:15:00	656	43.6	63.6	55.1	48.5	44.5	39.4	38.3	38.1	37.7
23/8/2022 3:45	0:15:00	657	44.3	68.8	55.3	46.4	44.2	39.8	38.5	38.3	37.9
23/8/2022 4:00	0:15:00	658	46.7	76.4	57.4	51	47	39.7	38.3	38	37.6
23/8/2022 4:15	0:15:00	659	40	57.2	46.2	42.3	41	39	38	37.8	37.4
23/8/2022 4:30	0:15:00	660	47.8	73.8	59.9	51.9	46.1	39.6	38.2	37.9	37.5
23/8/2022 4:45	0:15:00	661	50.7	82.8	57.2	49.3	46	39.8	38.2	37.9	37.5
23/8/2022 5:00	0:15:00	662	44.5	63	57.1	49.8	46.6	39.6	38.1	37.8	37.4
23/8/2022 5:15	0:15:00	663	43.7	75	51.7	45.4	43.2	39.8	38.3	38	37.5
23/8/2022 5:30	0:15:00	664	44.8	74.4	54.3	47.2	44.1	39.6	38.4	38.2	37.8
23/8/2022 5:45	0:15:00	665	47.7	79.9	54.4	47.1	44.5	40.2	38.6	38.4	38
23/8/2022 6:00	0:15:00	666	41.4	64	50.1	43.6	41.7	39.1	38.2	38	37.7
23/8/2022 6:15	0:15:00	667	40.4	56.8	46.7	42.7	41.4	39.4	38.4	38.1	37.8
23/8/2022 6:30	0:15:00	668	46.6	72.8	58.1	47.9	45.8	40.8	38.7	38.4	37.7
23/8/2022 6:45	0:15:00	669	57.2	83.5	68.9	60.3	56.4	45.1	40.7	39.8	38.4
23/8/2022 7:00	0:15:00	670	65.4	100.8	69.2	59.8	55.9	48.1	43.2	42.2	40.5
23/8/2022 7:15	0:15:00	671	81.5	117.5	72.3	59.5	55.5	46.1	41.1	40.5	39.6
23/8/2022 7:30	0:15:00	672	51.5	77.2	61.2	56	53.6	46.6	42.7	41.9	40.6
23/8/2022 7:45	0:15:00	673	46.2	67.1	54	50.4	48.9	43.9	40.4	39.9	39.2
23/8/2022 8:00	0:15:00	674	49.6	72.7	59	54.4	52.2	45.6	41.5	40.8	39.7
23/8/2022 8:15	0:15:00	675	50.2	77.3	60.8	53.8	51	44.8	41.1	40.4	39.5
23/8/2022 8:30	0:15:00	676	48.1	69.9	57.5	52.7	50.6	44.4	41	40.5	39.6
23/8/2022 8:45	0:15:00	677	44.6	64.1	53	49.3	47.1	42.3	40.3	39.9	39.3
23/8/2022 9:00	0:15:00	678	47.7	67.8	56.5	53	50.4	44.2	41.2	40.6	39.9
23/8/2022 9:15	0:15:00	679	45.6	68.4	53.5	49.5	47.8	43.8	41.2	40.6	39.8
23/8/2022 9:30	0:15:00	680	45.5	65	54.2	49.5	47.4	43.1	40.7	40.1	39.3
23/8/2022 9:45	0:15:00	681	49.5	65.1	58.6	54.9	52.7	46.3	43.1	42.4	41.1
23/8/2022 10:00	0:15:00	682	57.5	84.6	70.1	62.1	56.7	47	42.9	42.2	40.7
23/8/2022 10:15	0:15:00	683	50.8	78.2	61.6	54	50.9	44.7	41.7	41.1	40
23/8/2022 10:30	0:15:00	684	61.4	84	71.1	66	64	56.2	46.9	45.7	44.4
23/8/2022 10:45	0:15:00	685	60.2	87.6	69.1	65.4	63.5	55.5	46.1	44.9	44.1
23/8/2022 11:00	0:15:00	686	57.7	81.8	66.4	63.2	61.3	52.8	46	45.3	44.6
23/8/2022 11:15	0:15:00	687	56.5	78.5	66.8	61.4	59.3	51.3	45.1	44.6	44.1
23/8/2022 11:30	0:15:00	688	57.7	84.5	68.9	62.7	59.9	49.6	44.7	44.3	43.8

23/8/2022 11:45	0:15:00	689	54.8	77.6	66.1	60.4	57.5	49.1	45.5	45	44.5
23/8/2022 12:00	0:15:00	690	57	76.7	69.1	62.6	59.2	49.6	45.3	44.9	44.3
23/8/2022 12:15	0:15:00	691	61	85.2	71.9	66	62.8	51.7	45.1	43.7	39.7
23/8/2022 12:30	0:15:00	692	60.3	84.4	73	64.5	61.2	49	40.2	39.3	38.3
23/8/2022 12:45	0:15:00	693	53.2	76	64.4	58.5	55.7	46.9	41.1	40.4	39.5
23/8/2022 13:00	0:15:00	694	45.9	64.9	56.2	50.6	48.3	42.1	40.1	39.7	39.2
23/8/2022 13:15	0:15:00	695	48.7	74.3	58.9	54.2	51.5	42.2	39.6	39.3	38.7
23/8/2022 13:30	0:15:00	696	54.5	72.3	66.9	62.9	55.4	43.4	40.1	39.6	38.8
23/8/2022 13:45	0:15:00	697	45.8	71.3	55.9	48.7	46.4	41.9	40.5	40.3	39.8
23/8/2022 14:00	0:15:00	698	47.9	63.3	57.5	53.3	51	44.2	41.3	40.9	40.3
23/8/2022 14:15	0:15:00	699	50.9	71.2	63.4	56.2	52.7	43.4	40.8	40.4	39.9
23/8/2022 14:30	0:15:00	700	51.4	71.3	62.8	57.5	54.4	45.3	41.4	40.8	40.1
23/8/2022 14:45	0:15:00	701	52.6	72.3	63.7	57.1	54.6	47.6	42.9	42	40.9
23/8/2022 15:00	0:15:00	702	55	80.1	66.8	60.7	57.1	47.6	42.8	41.9	40.7
23/8/2022 15:15	0:15:00	703	51	70.6	61.3	56.7	54.1	45.6	41.6	41.1	40.2
23/8/2022 15:30	0:15:00	704	49	67.9	60	54.7	51.6	43.6	41.1	40.7	40.1
23/8/2022 15:45	0:15:00	705	44.2	64.8	53.2	47.8	45.2	41.6	40.4	40.1	39.6
23/8/2022 16:00	0:15:00	706	52.4	77.5	63.4	56.6	52.3	44.1	41.1	40.6	39.9
23/8/2022 16:15	0:15:00	707	52.9	77.3	64.5	57	53.3	43.5	40.7	40.3	39.7
23/8/2022 16:30	0:15:00	708	49.1	69.5	60.2	55.2	52.4	42.7	40.5	40.1	39.6
23/8/2022 16:45	0:15:00	709	49.5	79.5	59.6	53	48.2	41.9	40.3	40	39.6
23/8/2022 17:00	0:15:00	710	52.2	74.9	64.4	56.7	53.1	43.7	40.8	40.4	39.8
23/8/2022 17:15	0:15:00	711	48.3	69	59.6	54.4	50.6	42.2	39.9	39.5	38.9
23/8/2022 17:30	0:15:00	712	54.9	77.3	66.6	61.3	57.9	44.2	39.9	39.4	38.7
23/8/2022 17:45	0:15:00	713	54.2	74.4	65.8	61.3	57.7	44.5	40.5	40	39.1
23/8/2022 18:00	0:15:00	714	51.6	70.9	62.4	57.9	55.1	45.2	41.3	40.6	39.6
23/8/2022 18:15	0:15:00	715	63.5	87.1	75.4	69.1	65.6	54	41.4	40.4	39.4
23/8/2022 18:30	0:15:00	716	54.3	81.9	65.3	60	56.8	42.9	40.1	39.7	39.3
23/8/2022 18:45	0:15:00	717	50.6	77.5	61.6	55.1	52	43.4	40.6	40.2	39.7
23/8/2022 19:00	0:15:00	718	55.9	81	66.4	62.7	58.1	46.9	41.8	41.1	40.1
23/8/2022 19:15	0:15:00	719	58.5	90.1	66.5	64	61.1	44.2	39.6	39.2	38.5
23/8/2022 19:30	0:15:00	720	49.2	79.6	58	49	46	41.1	39	38.7	38
23/8/2022 19:45	0:15:00	721	48.8	74.9	59.7	49.8	46.7	41.4	38.8	38.3	37.7
23/8/2022 20:00	0:15:00	722	43.6	67.8	54.2	46.4	44	39.8	38.4	38.1	37.7

23/8/2022 20:15	0:15:00	723	45.3	66.6	56	50.2	47.3	41.2	38.7	38.3	37.8
23/8/2022 20:30	0:15:00	724	43.6	65	52.9	48.9	46.4	40	38.2	37.9	37.4
23/8/2022 20:45	0:15:00	725	55.7	83.7	67.1	60	54.7	41.3	38.8	38.4	37.9
23/8/2022 21:00	0:15:00	726	59.5	82.2	71.4	64.4	61.6	50.8	41.7	40.3	38.9
23/8/2022 21:15	0:15:00	727	53.6	74.5	66	59	55.7	43.9	39.8	39.5	38.9
23/8/2022 21:30	0:15:00	728	49.2	71.4	61.9	53.1	49.3	41.2	39.2	38.8	38.2
23/8/2022 21:45	0:15:00	729	54.6	86.1	64.7	55.1	51.4	41.6	39.8	39.5	38.9
23/8/2022 22:00	0:15:00	730	58.3	80.6	67.5	62.9	61	53.6	46.7	45.3	43.3
23/8/2022 22:15	0:15:00	731	55.1	77	64.7	60.9	58.8	49.5	46.4	46.2	45.7
23/8/2022 22:30	0:15:00	732	56.8	82.3	67	62	59.7	50.9	45.8	44.8	42.9
23/8/2022 22:45	0:15:00	733	55	71.1	65.4	61.4	59.2	47.5	42.5	42.1	41.4
23/8/2022 23:00	0:15:00	734	55.7	83.8	65.6	60.3	57.6	46.4	42.9	42.5	41.9
23/8/2022 23:15	0:15:00	735	52.9	74.9	63.5	58.9	56.4	44.8	40.4	39.6	38.9
23/8/2022 23:30	0:15:00	736	44.4	69	54.2	48.4	45.8	41.3	39.5	39.2	38.7
23/8/2022 23:45	0:15:00	737	41.7	53.4	48	44	42.8	40.9	39.9	39.7	39.4
24/8/2022 0:00	0:15:00	738	50.2	67.9	61.2	55.7	53	44.5	40.6	40.2	39.7
24/8/2022 0:15	0:15:00	739	49.2	73.9	61.4	54.2	50.6	42.1	40.1	39.8	39.4
24/8/2022 0:30	0:15:00	740	47.8	75.7	58.8	52.3	48.3	41.5	40.2	40	39.7
24/8/2022 0:45	0:15:00	741	46.9	69.6	58.1	50.9	47.6	41.8	40.5	40.2	39.9
24/8/2022 1:00	0:15:00	742	48.2	78.9	56	48.8	46	41.2	40.1	39.9	39.6
24/8/2022 1:15	0:15:00	743	47.8	72.8	58.3	52.9	50.1	41.8	40.1	39.9	39.5
24/8/2022 1:30	0:15:00	744	55.3	80.2	65.2	60.4	58.1	50.3	42.2	40.9	39.8
24/8/2022 1:45	0:15:00	745	57	85.8	66.5	61.7	59.4	51	45.8	45	44.1
24/8/2022 2:00	0:15:00	746	56.7	75.9	68.1	63	59.9	49.3	46.7	46.4	46
24/8/2022 2:15	0:15:00	747	55.9	82	65.8	61.1	58.6	49.9	47	46.6	46.2
24/8/2022 2:30	0:15:00	748	57.3	74.6	67.1	63.1	61	52.1	46.6	46.1	45.6
24/8/2022 2:45	0:15:00	749	55.7	80.9	65.1	61	58.8	50.3	46.5	46.1	45.5
24/8/2022 3:00	0:15:00	750	58.9	77.1	67.8	64	62.4	55.3	48.3	47.3	46.2
24/8/2022 3:15	0:15:00	751	62.1	89.6	71.9	61.8	58.4	49.1	46.1	45.8	45.4
24/8/2022 3:30	0:15:00	752	52.1	70.7	61.8	58.4	55.2	46.9	45.7	45.5	45.2
24/8/2022 3:45	0:15:00	753	55.2	81.9	63.7	60	57.6	48.3	45.9	45.7	45.3
24/8/2022 4:00	0:15:00	754	56	79	66	62.5	60.5	46.3	44.3	44.1	43.6
24/8/2022 4:15	0:15:00	755	45.8	63.6	54.1	48.6	46.6	44.4	43.6	43.5	43.1
24/8/2022 4:30	0:15:00	756	46	69.1	52.5	47.7	46.4	44.6	43.8	43.6	43.3

24/8/2022 4:45	0:15:00	757	48.4	69.5	58.4	52.7	49.8	45	43.9	43.7	43.4
24/8/2022 5:00	0:15:00	758	47.1	68.4	56.7	50	47.3	44.8	44.1	43.9	43.6
24/8/2022 5:15	0:15:00	759	48.3	72	58.5	51.8	48.8	45	44.1	43.9	43.6
24/8/2022 5:30	0:15:00	760	55.5	78.2	66.1	61.7	59.4	46.4	43.8	43	41.6
24/8/2022 5:45	0:15:00	761	55.7	76.2	65.8	61.1	59.1	51.3	46.2	45.7	45.2
24/8/2022 6:00	0:15:00	762	49.9	77.6	60	53.4	50.6	46.4	45.6	45.4	45.1
24/8/2022 6:15	0:15:00	763	50.6	71	63	53.5	50.7	46.4	45.4	45.3	45
24/8/2022 6:30	0:15:00	764	49.7	67.9	60.9	53.7	50.7	46.5	45.5	45.4	45.1
24/8/2022 6:45	0:15:00	765	47.5	67.7	53.8	49.7	48.3	46.4	45.6	45.4	45.2
24/8/2022 7:00	0:15:00	766	53.8	70.2	61.9	58.5	56.6	51.3	47.4	46.6	45.6
24/8/2022 7:15	0:15:00	767	54.7	76.2	64.8	60	57.4	50.2	46.8	46.4	45.8
24/8/2022 7:30	0:15:00	768	55.6	80.1	66.4	60.3	57.1	48.6	46.4	46.2	45.8
24/8/2022 7:45	0:15:00	769	52	71.2	62.6	57.9	54.6	47.4	46	45.7	45.2
24/8/2022 8:00	0:15:00	770	52	75.7	64.2	54.5	51.7	46.9	45.9	45.8	45.5
24/8/2022 8:15	0:15:00	771	52.9	76.4	63.1	57.8	54.9	47.3	45.8	45.6	45.4
24/8/2022 8:30	0:15:00	772	50.3	71.5	61.4	54.7	51.1	46.5	45.6	45.5	45.3
24/8/2022 8:45	0:15:00	773	57.1	75.8	67.5	63.4	60.9	50.8	46.3	46	45.6
24/8/2022 9:00	0:15:00	774	58.3	79.7	68.6	64.3	61.8	51.8	45.9	45.6	45.2
24/8/2022 9:15	0:15:00	775	51.7	71.7	62	56.7	54.3	47.6	45.8	45.6	45.3
24/8/2022 9:30	0:15:00	776	57.3	77.4	67.6	62.9	60.6	52.4	47.8	47.1	46.1
24/8/2022 9:45	0:15:00	777	53.4	76	63	59.7	56.2	48.2	46.5	46.3	45.9
24/8/2022 10:00	0:15:00	778	51.9	71.9	62.1	58.1	54.9	47.6	46.3	46	45.7
24/8/2022 10:15	0:15:00	779	51.6	69.6	61.4	57.3	53.6	47.8	46.4	46.2	45.8
24/8/2022 10:30	0:15:00	780	57.8	80.2	68.1	63.2	60.6	51.6	47.6	47	46.4
24/8/2022 10:45	0:15:00	781	53.4	78.3	63.4	57.5	54.5	48.9	47.2	46.8	46.2
24/8/2022 11:00	0:15:00	782	55.1	74	66.3	61.3	57.3	48.8	46.8	46.5	46
24/8/2022 11:15	0:15:00	783	54.7	73.9	65.6	60.5	57.8	48.9	46.9	46.6	46.2
24/8/2022 11:30	0:15:00	784	55.9	74.9	66	61.8	59.5	49.8	47.1	46.8	46.2
24/8/2022 11:45	0:15:00	785	53.9	76.1	64.6	58.6	55.9	49.1	47	46.6	45.9
24/8/2022 12:00	0:15:00	786	51.9	69.1	60.4	57.3	54.5	48.9	47.3	47.1	46.6
24/8/2022 12:15	0:15:00	787	53.6	74.4	63.3	56.2	53.7	49	47.4	47.1	46.7
24/8/2022 12:30	0:15:00	788	51.3	66.8	60.4	55	53.5	49	47.4	47.1	46.7
24/8/2022 12:45	0:15:00	789	54.2	71.5	64.5	59.7	57	50.1	47.4	47	46.5
24/8/2022 13:00	0:15:00	790	52.3	73.2	62.8	56.3	54.1	49.1	47.5	47.2	46.7

24/8/2022 13:15	0:15:00	791	53.8	75.8	64.6	58.1	55.6	49.6	47.3	46.9	46.3
24/8/2022 13:30	0:15:00	792	53.9	73.7	64.1	58.9	56.2	50	47.6	47.1	46.4
24/8/2022 13:45	0:15:00	793	51	69.2	61.3	55.3	52.3	48.4	46.8	46.5	45.9
24/8/2022 14:00	0:15:00	794	53.9	71.6	65.3	60	56.6	48.9	47	46.7	46.2
24/8/2022 14:15	0:15:00	795	54	76.7	63.7	59.3	57.1	49.8	47.5	47.1	46.4
24/8/2022 14:30	0:15:00	796	52.2	77.1	62	56.9	54.3	48.6	47	46.6	46
24/8/2022 14:45	0:15:00	797	49.8	73.8	59.3	52.2	50	47.8	46.8	46.6	46.2
24/8/2022 15:00	0:15:00	798	53.2	70.9	62.8	58.7	56.3	49	46.8	46.5	46.1
24/8/2022 15:15	0:15:00	799	53.4	71.7	64.6	59.7	56.1	47.6	46.2	46	45.8
24/8/2022 15:30	0:15:00	800	54.8	77.9	65.5	61	58	48.5	46.2	46	45.5
24/8/2022 15:45	0:15:00	801	55.4	77	65.4	60.8	58.7	49.5	45.1	44.8	44.4
24/8/2022 16:00	0:15:00	802	53.5	75.2	63.3	59.5	57.1	46.9	44.6	44.5	44.1
24/8/2022 16:15	0:15:00	803	53.8	78.7	64	58.5	56.5	48.2	45.1	44.8	44.3
24/8/2022 16:30	0:15:00	804	54.2	72.4	63.9	60.1	58	48.5	45	44.8	44.5
24/8/2022 16:45	0:15:00	805	55.6	73.8	69.3	60.4	56.8	46.6	45.1	44.9	44.6
24/8/2022 17:00	0:15:00	806	56.5	80.3	70.1	61	56.1	46.9	45.4	45.2	45
24/8/2022 17:15	0:15:00	807	47.2	61.5	53.7	49.9	48.5	46.2	45.4	45.2	44.9
24/8/2022 17:30	0:15:00	808	54.2	73.8	66.5	59.7	55.9	47.1	45.5	45.3	45
24/8/2022 17:45	0:15:00	809	52.9	76.7	64.8	58.8	54.7	46.3	45.4	45.2	44.9
24/8/2022 18:00	0:15:00	810	48.9	70.3	57.2	52.8	50.5	46.3	45.4	45.2	45
24/8/2022 18:15	0:15:00	811	51.1	71.2	61	55.6	52.8	47.5	45.8	45.6	45.2
24/8/2022 18:30	0:15:00	812	47.7	62.8	54.8	50.9	49.2	46.4	45.5	45.3	45
24/8/2022 18:45	0:15:00	813	48.8	73.5	55.7	52.4	51.4	46.7	45.5	45.3	45
24/8/2022 19:00	0:15:00	814	51.3	74.7	57.9	54.8	53.4	49.7	47.2	46.7	46.1
24/8/2022 19:15	0:15:00	815	51	69.6	59.4	56.1	54.1	48	46.1	45.8	45.4
24/8/2022 19:30	0:15:00	816	48.6	75.5	56.6	50.9	48.8	46.2	45.2	45.1	44.7
24/8/2022 19:45	0:15:00	817	48.9	68.6	55.8	51.4	50.1	47.5	46.7	46.6	46.4
24/8/2022 20:00	0:15:00	818	51	66.8	57.7	54.9	53.5	49.5	47.2	46.9	46.5
24/8/2022 20:15	0:15:00	819	55.1	80.7	63.8	59.1	57	50.6	47	46.7	46.3
24/8/2022 20:30	0:15:00	820	53.9	77.4	62.7	58.6	56.5	49.1	46.8	46.5	46.2
24/8/2022 20:45	0:15:00	821	56.5	75.5	67.6	63.7	60.9	47.9	46.8	46.7	46.4
24/8/2022 21:00	0:15:00	822	53.3	76.9	65.1	58.5	54.3	47.4	46.6	46.5	46.2
24/8/2022 21:15	0:15:00	823	49.7	65.7	57.4	53.9	52.3	47.6	46.5	46.4	46.2
24/8/2022 21:30	0:15:00	824	52.7	71.7	61.7	57.8	56	49.3	46.7	46.6	46.3

24/8/2022 21:45	0:15:00	825	49.5	71.4	58.5	52.3	50.3	47.1	46.5	46.4	46.1
24/8/2022 22:00	0:15:00	826	48	61	53.9	50.2	48.9	47.2	46.7	46.6	46.3
24/8/2022 22:15	0:15:00	827	51.8	70	64.9	54.3	51.2	47.4	46.6	46.5	46.3
24/8/2022 22:30	0:15:00	828	50.1	65.8	59.2	55.6	52.8	47.3	46.6	46.4	46.2
24/8/2022 22:45	0:15:00	829	56.4	77.7	68.9	61.9	57.8	48.3	46.7	46.5	46.3
24/8/2022 23:00	0:15:00	830	54.4	76.7	63.7	60	58	49.7	47.1	46.9	46.5
24/8/2022 23:15	0:15:00	831	59.1	79.8	71.7	64.4	61.5	48.4	46.9	46.7	46.3
24/8/2022 23:30	0:15:00	832	50	66.2	61.3	54	49.9	47.4	46.9	46.8	46.5
24/8/2022 23:45	0:15:00	833	51	65.3	62.5	55.6	53.3	47.6	46.9	46.7	46.5
25/8/2022 0:00	0:15:00	834	51	66.9	62.3	56.4	52.1	47.4	46.8	46.7	46.5
25/8/2022 0:15	0:15:00	835	48.3	62	55.3	51	49.1	47.3	46.8	46.6	46.4
25/8/2022 0:30	0:15:00	836	48.6	65.5	55.9	51.2	49.6	47.4	46.8	46.7	46.4
25/8/2022 0:45	0:15:00	837	48.2	62.1	54	49.9	48.6	47.5	47	46.8	46.6
25/8/2022 1:00	0:15:00	838	48.7	66.9	56.5	51.2	49	47.4	46.8	46.7	46.4
25/8/2022 1:15	0:15:00	839	48.7	60.9	52.9	49.7	49.1	48.3	47.7	47.4	47
25/8/2022 1:30	0:15:00	840	49.2	64.4	57.2	51.4	49.7	48	47.3	47.1	46.9
25/8/2022 1:45	0:15:00	841	48.2	67.7	53	48.5	48.1	47.5	47	46.9	46.7
25/8/2022 2:00	0:15:00	842	51.3	70.4	63	54.8	51.3	47.9	47.2	47	46.8
25/8/2022 2:15	0:15:00	843	52.5	70.8	63.7	58.4	54.9	47.8	46.6	46.4	46.1
25/8/2022 2:30	0:15:00	844	50.2	71.9	59.6	54.2	51.7	47.3	46.5	46.4	46.2
25/8/2022 2:45	0:15:00	845	48.9	68.3	57.9	53.1	49.1	46.9	46.5	46.4	46.2
25/8/2022 3:00	0:15:00	846	50.6	66.8	61.4	55.7	52.5	47	46.5	46.3	46.2
25/8/2022 3:15	0:15:00	847	49.6	71.2	58.7	53.6	49.5	47	46.5	46.3	46.2
25/8/2022 3:30	0:15:00	848	48.9	68.8	58	52.5	49	46.8	46.3	46.2	46
25/8/2022 3:45	0:15:00	849	47.5	61	54	49	47.7	46.8	46.4	46.3	46.1
25/8/2022 4:00	0:15:00	850	48.6	64.7	55.5	51.5	49.8	47.2	46.6	46.5	46.3
25/8/2022 4:15	0:15:00	851	47.3	60.9	53.1	48.4	47.4	46.7	46.3	46.2	46
25/8/2022 4:30	0:15:00	852	47.7	69.6	54.3	49.6	47.8	46.7	46.3	46.2	46
25/8/2022 4:45	0:15:00	853	46.9	59.8	50.8	47.4	47.1	46.6	46.2	46.1	45.9
25/8/2022 5:00	0:15:00	854	54.3	76.4	65.8	60.6	57.6	46.9	46.3	46.2	45.9
25/8/2022 5:15	0:15:00	855	50.5	68.2	61.2	55	52	46.8	46	45.9	45.7
25/8/2022 5:30	0:15:00	856	48.4	74.9	57.1	49.6	47.6	46.6	46.2	46.2	46
25/8/2022 5:45	0:15:00	857	48.5	66.4	56.7	51.9	49.6	46.7	46.3	46.2	46
25/8/2022 6:00	0:15:00	858	52.5	80.1	57.6	50.8	49	47.1	46.5	46.4	46.2

25/8/2022 6:15	0:15:00	859	52.2	74.4	63.1	58.1	54.1	47.7	46.9	46.7	46.4
25/8/2022 6:30	0:15:00	860	54	71.9	66.7	60.3	54	47.5	46.8	46.7	46.5
25/8/2022 6:45	0:15:00	861	51.4	67.7	61.8	57.5	54.2	47.4	46.8	46.7	46.5
25/8/2022 7:00	0:15:00	862	52.5	72.2	61.5	57	54.8	49.5	47.5	47.2	46.9
25/8/2022 7:15	0:15:00	863	50.1	79.1	56	50.6	49.4	47.8	47.1	46.9	46.7
25/8/2022 7:30	0:15:00	864	51.5	69.8	61.3	57.9	53.2	47.8	47	46.9	46.6
25/8/2022 7:45	0:15:00	865	50.4	67.1	59.5	55.6	52	47.7	46.9	46.8	46.6
25/8/2022 8:00	0:15:00	866	50.5	70.3	59.9	54.8	52.3	47.7	46.9	46.8	46.6
25/8/2022 8:15	0:15:00	867	51.5	70.1	61.2	56.4	53.9	48.3	47	46.8	46.5
25/8/2022 8:30	0:15:00	868	51.5	74.1	60.9	54.6	51.7	47.9	47	46.8	46.5
25/8/2022 8:45	0:15:00	869	51.1	73.9	59.5	54.6	52.7	48	46.8	46.6	46.2
25/8/2022 9:00	0:15:00	870	52.8	74	65.6	54.3	51.4	47.9	47.4	47.3	47.1
25/8/2022 9:15	0:15:00	871	53	76.5	64.9	54.9	52.6	48.2	47.2	47	46.6
25/8/2022 9:30	0:15:00	872	54.6	75.1	66.5	60.1	57.3	48.7	47.1	46.9	46.6
25/8/2022 9:45	0:15:00	873	61.3	78.8	72.6	69.4	66.4	50.3	47.7	47.4	47
25/8/2022 10:00	0:15:00	874	56.8	76.6	68.3	62.5	59.9	50.2	47.4	47.1	46.7
25/8/2022 10:15	0:15:00	875	51	78	59.5	54.4	51.9	48	46.9	46.7	46.3
25/8/2022 10:30	0:15:00	876	50.5	76	59	54	51.5	47.5	46.5	46.4	46.1
25/8/2022 10:45	0:15:00	877	49.3	65.6	59.5	52.8	50.2	47.3	46.4	46.3	46
25/8/2022 11:00	0:15:00	878	49.9	74.2	57.7	53.7	51.9	47.6	46.3	46.2	45.8
25/8/2022 11:15	0:15:00	879	54.4	72.5	64.9	60	57.7	49.3	47	46.8	46.4
25/8/2022 11:30	0:15:00	880	55.8	75.9	66.6	61.6	58.8	50.1	47.8	47.5	46.9
25/8/2022 11:45	0:15:00	881	50.8	64.5	59.6	54.3	52.6	48.7	47.7	47.5	47.2
25/8/2022 12:00	0:15:00	882	51	72	63.2	52.6	50.8	47.7	47.1	47	46.8
25/8/2022 12:15	0:15:00	883	51.6	74.4	63.2	54.1	52.3	48.1	47.1	46.9	46.3
25/8/2022 12:30	0:15:00	884	55.2	84.6	66.3	58.7	55.3	47.5	46	45.8	45.5
25/8/2022 12:45	0:15:00	885	54.4	76.9	64.9	60.5	57.7	48.1	46	45.9	45.6
25/8/2022 13:00	0:15:00	886	54.9	73.1	65.1	61.1	58.5	49.6	46.3	46.1	45.8
25/8/2022 13:15	0:15:00	887	55.5	77.6	65	61.3	59.2	50.7	46.5	46.2	45.9
25/8/2022 13:30	0:15:00	888	56.1	76.4	67.8	62.1	59.2	48.3	46.2	46	45.7
25/8/2022 13:45	0:15:00	889	63.1	84.3	73.8	69.4	66.7	56	47.7	47	46.4
25/8/2022 14:00	0:15:00	890	56.5	76.2	67.1	62.7	60.2	49.7	46.6	46.3	45.9
25/8/2022 14:15	0:15:00	891	60.7	84.3	70.6	66.7	64.3	53.1	46.8	45.5	44
25/8/2022 14:30	0:15:00	892	57.5	76	67.1	63.7	61.5	52.2	46.5	45.8	45

25/8/2022 14:45	0:15:00	893	53	73.9	63.5	58.4	55.8	48.7	47.1	46.8	46.4
25/8/2022 15:00	0:15:00	894	54.3	74.8	63.7	59.6	57	50.2	47.6	47.2	46.7
25/8/2022 15:15	0:15:00	895	51.8	70.9	63.3	56.4	53.6	48.3	47.2	47	46.7
25/8/2022 15:30	0:15:00	896	50	70.7	59.2	53.8	51.1	47.7	47	46.8	46.5
25/8/2022 15:45	0:15:00	897	54.1	81.8	64.9	58.6	54.3	47.6	46.7	46.5	46.1
25/8/2022 16:00	0:15:00	898	60.1	81.7	72.4	66.2	62.5	49.6	47.3	47.1	46.7
25/8/2022 16:15	0:15:00	899	48.7	67.8	54.7	50.8	49.5	47.7	47.1	47	46.8
25/8/2022 16:30	0:15:00	900	49	71.1	55.2	50.9	49.6	47.8	47.2	47	46.8
25/8/2022 16:45	0:15:00	901	50.5	72.3	58.7	53.7	51.6	48.2	47.5	47.3	47.1
25/8/2022 17:00	0:15:00	902	48.5	68.5	53.3	49.5	48.8	47.9	47.4	47.3	47
25/8/2022 17:15	0:15:00	903	48.9	67.3	56.2	50.8	49.3	47.7	47.2	47.1	46.8
25/8/2022 17:30	0:15:00	904	52.1	72.3	63	57.1	53.5	48.1	47.3	47.2	46.9
25/8/2022 17:45	0:15:00	905	49.5	77.1	55.2	50.6	49.1	47.7	47.1	47	46.8
25/8/2022 18:00	0:15:00	906	50.7	70.9	61.1	55	51.8	47.7	47	46.9	46.7
25/8/2022 18:15	0:15:00	907	52.1	66.7	64.3	57.8	53.3	47.7	46.9	46.7	46.4
25/8/2022 18:30	0:15:00	908	51.5	71.7	61.3	56.6	53.7	47.9	46.9	46.8	46.5
25/8/2022 18:45	0:15:00	909	50.5	69.3	59.9	54.1	51.5	48.1	47.3	47.1	46.1
25/8/2022 19:00	0:15:00	910	52.3	70.1	60.5	56.3	54.4	49.8	47.9	47.6	47.2
25/8/2022 19:15	0:15:00	911	52.2	71.2	62	56.7	54.1	49.3	47.5	47.3	47
25/8/2022 19:30	0:15:00	912	52.6	80.5	60.8	54.6	51.9	48.6	47.5	47.3	46.9
25/8/2022 19:45	0:15:00	913	59.1	75.3	70.1	66.5	63.6	49.7	48.5	48.3	48
25/8/2022 20:00	0:15:00	914	61.3	80.8	71.2	68.6	66.2	50.9	49.1	48.9	48.5
25/8/2022 20:15	0:15:00	915	55.1	71.7	68.2	60.5	54.2	49.2	48.4	48.2	47.9
25/8/2022 20:30	0:15:00	916	50.2	64.5	56.9	53.1	51.4	49.1	48.3	48.1	47.7
25/8/2022 20:45	0:15:00	917	55	76.9	67.5	60.5	54.3	49	48	47.9	47.6
25/8/2022 21:00	0:15:00	918	57.3	79.1	70.4	61.1	57	49.7	48.3	48.1	47.7
25/8/2022 21:15	0:15:00	919	50.3	67.6	55.8	52.9	51.8	49.2	48.2	48	47.7
25/8/2022 21:30	0:15:00	920	50	64.9	55.6	52.8	51.6	49.1	48.2	48	47.7
25/8/2022 21:45	0:15:00	921	49.5	64.9	57.1	51.2	49.9	48.4	47.9	47.7	47.5
25/8/2022 22:00	0:15:00	922	49.6	65.9	56.2	51.5	50.4	48.6	47.9	47.8	47.5
25/8/2022 22:15	0:15:00	923	51.1	71.3	59.8	53.3	51.6	48.8	48	47.8	47.6
25/8/2022 22:30	0:15:00	924	49.8	68	58.4	51.6	49.9	48.4	47.8	47.6	47.4
25/8/2022 22:45	0:15:00	925	49	62.6	53.2	50.2	49.5	48.6	48	47.9	47.7
25/8/2022 23:00	0:15:00	926	51.4	68.5	62.7	54.5	52	49	48.3	48.1	47.8

25/8/2022 23:15	0:15:00	927	49.3	65.5	55.6	51.1	49.5	48.4	47.9	47.8	47.5
25/8/2022 23:30	0:15:00	928	49	65.7	54.7	49.7	49.3	48.5	47.9	47.8	47.5
25/8/2022 23:45	0:15:00	929	48.7	65.1	50.7	49.4	49.1	48.4	47.9	47.8	47.5
26/8/2022 0:00	0:15:00	930	49.2	65.8	54.7	50.1	49.5	48.6	48	47.8	47.6
26/8/2022 0:15	0:15:00	931	49.1	62.9	54.5	49.9	49.4	48.6	48	47.9	47.6
26/8/2022 0:30	0:15:00	932	49.8	72	56.8	51	49.8	48.7	48.1	48	47.7
26/8/2022 0:45	0:15:00	933	48.8	58.7	50.8	49.6	49.3	48.6	48	47.9	47.6
26/8/2022 1:00	0:15:00	934	49.4	64.9	54	50.8	50	48.8	48.1	47.9	47.7
26/8/2022 1:15	0:15:00	935	48.8	66.8	52.5	50	49.3	48.4	47.8	47.7	47.4
26/8/2022 1:30	0:15:00	936	50.2	70.5	58.8	51.2	49.5	48.5	47.9	47.7	47.5
26/8/2022 1:45	0:15:00	937	48.9	66.2	52.2	50.1	49.5	48.5	47.9	47.7	47.4
26/8/2022 2:00	0:15:00	938	49	65.2	53	50.1	49.5	48.6	48	47.8	47.6
26/8/2022 2:15	0:15:00	939	48.5	56.7	50.1	49.2	49	48.3	47.8	47.7	47.4
26/8/2022 2:30	0:15:00	940	48.4	55.3	49.6	49.1	48.9	48.3	47.8	47.6	47.4
26/8/2022 2:45	0:15:00	941	48.4	53.9	49.6	49.1	48.9	48.3	47.8	47.7	47.5
26/8/2022 3:00	0:15:00	942	48.5	61.2	49.9	49.2	49	48.4	47.9	47.7	47.5
26/8/2022 3:15	0:15:00	943	48.7	64.1	50.1	49.3	49.1	48.5	48	47.8	47.6
26/8/2022 3:30	0:15:00	944	48.9	59.3	54.3	49.5	49.2	48.5	47.9	47.6	47
26/8/2022 3:45	0:15:00	945	49.2	62	56.8	49.8	49.2	48.4	47.8	47.7	47.4
26/8/2022 4:00	0:15:00	946	48.9	65.2	51.9	49.6	49.3	48.5	48	47.8	47.6
26/8/2022 4:15	0:15:00	947	48.4	57.6	49.9	49.1	48.9	48.3	47.8	47.7	47.4
26/8/2022 4:30	0:15:00	948	48.9	58.5	51.3	49.7	49.4	48.6	48.1	47.9	47.6
26/8/2022 4:45	0:15:00	949	49.2	64.6	51.8	50.3	49.9	48.9	48.2	48	47.8
26/8/2022 5:00	0:15:00	950	48.9	63.7	52.5	49.9	49.3	48.5	47.9	47.8	47.6
26/8/2022 5:15	0:15:00	951	48.7	66.9	52	49.9	49.3	48.3	47.7	47.6	47.3
26/8/2022 5:30	0:15:00	952	49.1	68.7	54.6	50.3	49.3	48.2	47.7	47.5	47.3
26/8/2022 5:45	0:15:00	953	49.3	67.7	55.3	51.3	50.1	48.3	47.7	47.6	47.3
26/8/2022 6:00	0:15:00	954	49.9	70.5	56.9	51.9	50.6	48.5	47.7	47.6	47.3
26/8/2022 6:15	0:15:00	955	51.6	75.9	61.3	54.9	51.8	48.3	47.6	47.5	47.2
26/8/2022 6:30	0:15:00	956	49.9	72	57.8	51.7	50.2	48.2	47.6	47.4	47.2
26/8/2022 6:45	0:15:00	957	51.3	67.3	61.6	54.5	52.6	49.3	48	47.8	47.4
26/8/2022 7:00	0:15:00	958	52	66.6	60.6	55.7	54	50.2	48.6	48.3	47.9
26/8/2022 7:15	0:15:00	959	52.8	66.9	59.9	57.1	55.6	51	48.4	48.2	47.8
26/8/2022 7:30	0:15:00	960	58	76.9	69.4	64.5	61.2	50.4	48.2	48	47.6

26/8/2022 7:45	0:15:00	961	58.9	77.1	70.3	65.3	62.8	50.4	48	47.8	47.5
26/8/2022 8:00	0:15:00	962	58.8	76.5	70.5	65.7	62.2	49.3	47.9	47.8	47.5
26/8/2022 8:15	0:15:00	963	56.6	72.3	66.7	62.6	60.1	51.7	48.6	48.3	47.9
26/8/2022 8:30	0:15:00	964	55.3	72.4	66.6	61.7	58.3	49.3	46.1	45.8	45.5
26/8/2022 8:45	0:15:00	965	57.7	84.1	68.2	63.2	60.3	51.4	47	46.6	46
26/8/2022 9:00	0:15:00	966	57.6	77.8	69.1	64.6	61	48.7	42.5	41.4	40.2
26/8/2022 9:15	0:15:00	967	56.5	80.2	68.2	63.1	59.6	46.8	41.1	40.4	39.6
26/8/2022 9:30	0:15:00	968	57.4	79.2	69.4	63.7	60	49.1	43.6	42.1	39.7
26/8/2022 9:45	0:15:00	969	55.8	78.4	67.4	61.8	58	47.4	41.5	40.4	39
26/8/2022 10:00	0:15:00	970	60.1	87.1	71.3	65.7	62.6	50.1	42.1	40.5	38.7
26/8/2022 10:15	0:15:00	971	67.1	103.4	73	65.6	62.1	51.6	45.8	44.9	43.5
26/8/2022 10:30	0:15:00	972	60.6	90.6	71.1	61.9	57.9	48.7	45.2	44.7	44.2
26/8/2022 10:45	0:15:00	973	59	82.8	72.2	63.1	59.1	49.9	45.2	44.6	43.9
26/8/2022 11:00	0:15:00	974	60.2	91	70	62.5	58.8	49.4	44.8	44.3	43.6
26/8/2022 11:15	0:15:00	975	64.2	90.6	76.1	70.6	67.1	53.1	46.5	45.7	44.8
26/8/2022 11:30	0:15:00	976	59.3	83.1	71.9	63.9	60	49.9	46.1	45.5	44.9
26/8/2022 11:45	0:15:00	977	57.6	79.7	69.2	62.8	59.7	50.5	46.8	46.2	45.5
26/8/2022 12:00	0:15:00	978	53.9	75.1	64.4	59.4	56.9	49.1	45.9	45.5	44.9
26/8/2022 12:15	0:15:00	979	54.6	76.9	64.8	60.3	57.9	49.1	45.9	45.4	44.9
26/8/2022 12:30	0:15:00	980	55.4	76	65.2	61.2	58.8	50.2	46.2	45.7	45.1
26/8/2022 12:45	0:15:00	981	54.6	74	64.7	60.5	58.2	49.4	45.8	45.4	44.9
26/8/2022 13:00	0:15:00	982	55.3	74.1	65.3	61.2	58.8	50.9	46.7	46	45.2
26/8/2022 13:15	0:15:00	983	56.7	74.3	67.1	62.5	60.2	51.8	47	46.3	45.4
26/8/2022 13:30	0:15:00	984	57.7	79.1	67.7	63.2	60.9	52.5	47	46.3	45.3
26/8/2022 13:45	0:15:00	985	52.8	73.4	62.7	57.8	55.4	48.8	46	45.5	44.8
26/8/2022 14:00	0:15:00	986	55.7	86.1	66	57.4	54.2	46.8	42.5	41.8	40.7
26/8/2022 14:15	0:15:00	987	60.2	81.1	72.1	66.8	63.1	51.1	44.3	43.3	41.9
26/8/2022 14:30	0:15:00	988	57	84.8	68.3	60.8	57.4	48.1	42.5	41.7	41
26/8/2022 14:45	0:15:00	989	60	83.6	71.5	65.4	62.5	53.1	45.5	44.3	42.6
26/8/2022 15:00	0:15:00	990	62.2	79.4	70.9	68	66.4	57.5	50.3	49.7	49
26/8/2022 15:15	0:15:00	991	61.7	89.5	71.6	67.5	65	53.4	49.3	49	48.5
26/8/2022 15:30	0:15:00	992	60.3	80.1	71.8	66.7	63.8	52.4	48.9	48.6	48.1
26/8/2022 15:45	0:15:00	993	52.1	75.9	62	55	52.2	49.3	48.4	48.2	47.9
26/8/2022 16:00	0:15:00	994	61.1	80.1	72.6	67.8	64.6	52.2	49.1	48.8	48.3

26/8/2022 16:15	0:15:00	995	56.8	78.5	68.9	61.8	57.1	50.9	49.5	49.3	49
26/8/2022 16:30	0:15:00	996	54.5	75.9	65.6	58.5	54.6	50.6	49.5	49.4	49
26/8/2022 16:45	0:15:00	997	57.3	79.9	69.8	61.4	57.8	50.8	49.7	49.5	49.2
26/8/2022 17:00	0:15:00	998	62	78.6	73	68.6	66	52.6	49.7	49.5	49.1
26/8/2022 17:15	0:15:00	999	53.6	76	64	57.8	55.3	50.2	49.4	49.3	49
26/8/2022 17:30	0:15:00	1000	59.1	88.8	69.9	64.1	60.6	51.3	49.7	49.4	49.1
26/8/2022 17:45	0:15:00	1001	56.8	79.6	68.5	62.1	58.3	51.2	49.7	49.5	49
26/8/2022 18:00	0:15:00	1002	57.7	77.1	69.3	62.7	59.3	52.7	49.7	49.4	49
26/8/2022 18:15	0:15:00	1003	57.7	76.2	68.9	63.1	60.1	52.6	49.8	49.5	49.2
26/8/2022 18:30	0:15:00	1004	57	78	68.7	62.6	58	50.7	49.4	49.2	48.9
26/8/2022 18:45	0:15:00	1005	54.1	76.6	64.1	57.1	54.7	50.6	49.5	49.3	49
26/8/2022 19:00	0:15:00	1006	55.3	77.5	66.8	58.5	55.7	51.2	50	49.8	49.5
26/8/2022 19:15	0:15:00	1007	57.7	81.3	70.1	59.9	56.5	50.4	48.8	48.5	48.1
26/8/2022 19:30	0:15:00	1008	61.9	84.3	73.6	67.1	64.1	53.3	49.4	49	48.4
26/8/2022 19:45	0:15:00	1009	59.3	84	69.7	65.1	63.1	51.7	49.3	49	48.7
26/8/2022 20:00	0:15:00	1010	60.7	96.6	66.6	59.3	55.3	50.1	49	48.8	48.4
26/8/2022 20:15	0:15:00	1011	56.8	77	68.5	63	58.7	50.1	48.7	48.4	48.1
26/8/2022 20:30	0:15:00	1012	52.3	76.5	60.7	55.8	52.9	49.5	48.5	48.3	48
26/8/2022 20:45	0:15:00	1013	55.6	82	66.3	58.1	55.6	50.2	48.6	48.4	48
26/8/2022 21:00	0:15:00	1014	60.2	78.7	71.7	66.4	63.3	53.9	49.4	49	48.4
26/8/2022 21:15	0:15:00	1015	60	77.8	69.7	65.4	63.3	56.3	51.2	50.4	49.2
26/8/2022 21:30	0:15:00	1016	61	78.7	72.1	67.4	64.9	52.6	49.4	49	48.5
26/8/2022 21:45	0:15:00	1017	50.9	68.6	59.8	53.4	51.4	49.2	48.4	48.2	47.9
26/8/2022 22:00	0:15:00	1018	51.8	78.2	60.9	54.3	52.2	49.2	48.3	48.1	47.8
26/8/2022 22:15	0:15:00	1019	57.3	75.7	67.4	63	60.1	53.1	50.7	50.1	49.2
26/8/2022 22:30	0:15:00	1020	58.7	75.7	68.2	64.6	62.6	53.6	50.9	50.6	50.3
26/8/2022 22:45	0:15:00	1021	58	76	68.1	64.1	61.6	52.5	51	50.7	50.3
26/8/2022 23:00	0:15:00	1022	61.9	80.5	72.5	68.5	65.9	53.5	50.6	50.4	50.1
26/8/2022 23:15	0:15:00	1023	60.8	78.6	71.5	67.1	64.4	54.6	51.4	51	50.6
26/8/2022 23:30	0:15:00	1024	61.2	79.5	71.6	67.4	65	54.8	51.2	50.9	50.4
26/8/2022 23:45	0:15:00	1025	61.2	80.2	71.7	67.7	65.2	52.9	49.3	48.9	48.4
27/8/2022 0:00	0:15:00	1026	59.6	77	69.5	65.4	63.3	54.5	45.7	43.8	42.6
27/8/2022 0:15	0:15:00	1027	57.2	80.4	68.4	62.5	59.3	48.7	43.1	42.7	42
27/8/2022 0:30	0:15:00	1028	53.4	74.2	64.8	59.8	56.6	45.7	42.5	42.1	41.5

27/8/2022 0:45	0:15:00	1029	48.4	70.6	59.3	53	50.4	43.4	42.1	41.9	41.5
27/8/2022 1:00	0:15:00	1030	49.6	81.2	56.8	50	47.6	43.4	42.3	42.1	41.7
27/8/2022 1:15	0:15:00	1031	47.4	71.6	57.3	51.7	49.1	43.5	42.3	42	41.7
27/8/2022 1:30	0:15:00	1032	45.8	66.5	55.4	48.4	46.1	43.5	42.4	42.2	41.8
27/8/2022 1:45	0:15:00	1033	48.1	74.3	57.7	52.4	50	43.9	42.4	42.2	41.8
27/8/2022 2:00	0:15:00	1034	50.1	71.2	62.1	55.5	52.1	44	42.2	41.8	41.3
27/8/2022 2:15	0:15:00	1035	55.3	76.4	66.9	61.8	58.7	44.3	41.7	41.4	40.9
27/8/2022 2:30	0:15:00	1036	61.4	85.5	71.3	66.7	64.6	55.8	48.2	47.6	46.2
27/8/2022 2:45	0:15:00	1037	58.6	84.2	69.3	64.5	62.2	50.7	48	47.7	47.3
27/8/2022 3:00	0:15:00	1038	57.2	79.7	67.4	63.7	61.3	49.8	48.2	47.9	47.6
27/8/2022 3:15	0:15:00	1039	49.9	65.6	56.7	51.4	50.2	49	48.4	48.2	48
27/8/2022 3:30	0:15:00	1040	57.7	79	68.9	64.4	61.4	49.8	48.8	48.6	48.3
27/8/2022 3:45	0:15:00	1041	62	89.2	72.3	67.4	64.9	52.6	49	48.7	48.3
27/8/2022 4:00	0:15:00	1042	52.4	72.8	62.7	56.2	52.6	49.5	48.8	48.6	48.4
27/8/2022 4:15	0:15:00	1043	52	75.5	60.9	54.9	52.6	49.7	48.8	48.6	48.3
27/8/2022 4:30	0:15:00	1044	52.5	73.5	62.5	56.5	53.5	49.4	48.6	48.4	48.1
27/8/2022 4:45	0:15:00	1045	55	78.1	63.4	59.7	57.9	51.9	49.1	48.9	48.6
27/8/2022 5:00	0:15:00	1046	55.2	74.1	64.5	60.1	58.1	51.3	49.1	48.9	48.6
27/8/2022 5:15	0:15:00	1047	53	72	63.8	57.8	53.9	49.6	48.8	48.7	48.4
27/8/2022 5:30	0:15:00	1048	56.4	74.9	67.5	62.9	59.8	50.2	49	48.7	48.4
27/8/2022 5:45	0:15:00	1049	62.6	92.9	73.1	66.9	63.8	50.2	48.7	48.5	48.1
27/8/2022 6:00	0:15:00	1050	54	74	65.9	59	54	49.5	48.8	48.6	48.4
27/8/2022 6:15	0:15:00	1051	53.3	74.4	65.2	56	53.3	49.6	48.7	48.5	48.3
27/8/2022 6:30	0:15:00	1052	59.6	78.2	70.4	66.4	63.8	50.9	48.7	48.5	48.1
27/8/2022 6:45	0:15:00	1053	56	83.5	66.6	59.7	56.4	50.9	48.9	48.6	48.2
27/8/2022 7:00	0:15:00	1054	62.4	77.6	70.6	67.7	66.2	59.8	51.3	50.2	49.3
27/8/2022 7:15	0:15:00	1055	60.8	79.6	71.1	66.9	64.6	54.4	48.1	47.6	47.1
27/8/2022 7:30	0:15:00	1056	54.9	72.1	65.7	60.9	57.9	49.6	47.7	47.4	47
27/8/2022 7:45	0:15:00	1057	50.7	70.9	59.9	54	51.7	48.2	47.2	47.1	46.8
27/8/2022 8:00	0:15:00	1058	54.1	74.6	65.5	59.6	54.9	49	47.8	47.6	47.3
27/8/2022 8:15	0:15:00	1059	59.1	93.8	66.4	60.7	57.1	49	47.4	47.2	46.9
27/8/2022 8:30	0:15:00	1060	51.5	70.2	61.6	55.4	52.8	48.5	47.3	47.1	46.7
27/8/2022 8:45	0:15:00	1061	54.5	76.7	65.3	60.1	57.3	48.9	47.1	46.9	46.5
27/8/2022 9:00	0:15:00	1062	50.1	72.3	60	53.2	50.4	47.4	46.6	46.5	46.2

27/8/2022 9:15	0:15:00	1063	51.7	75	62.2	56.3	53.1	47.6	46.7	46.5	46.3
27/8/2022 9:30	0:15:00	1064	53.4	80.1	64.6	55.8	51.4	47.5	46.6	46.4	46.2
27/8/2022 9:45	0:15:00	1065	51.2	74.8	62.4	51.7	49.8	47.5	46.7	46.5	46.3
27/8/2022 10:00	0:15:00	1066	56.2	77	68.1	62.7	58.7	49.3	47.3	47	46.5
27/8/2022 10:15	0:15:00	1067	52.9	72	64.1	55.8	54	49.2	47.2	46.9	46.6
27/8/2022 10:30	0:15:00	1068	54.8	76.7	67	60.6	55.8	48.3	46.8	46.6	46.3
27/8/2022 10:45	0:15:00	1069	52.1	73.7	63.9	54.5	51	47.4	46.5	46.4	46.1
27/8/2022 11:00	0:15:00	1070	53.7	73.4	66	59.1	54.9	48	46.8	46.6	46.2
27/8/2022 11:15	0:15:00	1071	50.2	70.2	60.3	53.9	51.3	47.7	46.8	46.6	46.4
27/8/2022 11:30	0:15:00	1072	57.3	76.1	69.5	64.2	59.9	48.8	46.8	46.6	46.2
27/8/2022 11:45	0:15:00	1073	51.8	78.6	61.3	54.5	52.4	48.1	46.8	46.5	46.2
27/8/2022 12:00	0:15:00	1074	51.6	70.5	61.4	56	53.6	48.1	46.7	46.5	46.2
27/8/2022 12:15	0:15:00	1075	59.7	81.7	69.8	65.7	63.5	52.2	48.2	47.7	46.9
27/8/2022 12:30	0:15:00	1076	54.2	74.5	65.3	58.6	55.8	49.8	48.7	48.6	48.3
27/8/2022 12:45	0:15:00	1077	52.6	71.5	63.8	57.2	53	49.1	48.5	48.4	48.2
27/8/2022 13:00	0:15:00	1078	52.1	68.2	61.7	57.2	54.1	49.3	48.6	48.4	48.2
27/8/2022 13:15	0:15:00	1079	51.5	75.3	58.6	54.1	52.4	49.3	48.6	48.4	48.2
27/8/2022 13:30	0:15:00	1080	52	70.2	63	54.9	51.4	49	48.4	48.3	48.1
27/8/2022 13:45	0:15:00	1081	56.1	74.6	67.2	63.1	59.2	49.7	48.8	48.6	48.3
27/8/2022 14:00	0:15:00	1082	54.9	76.3	66.2	61.1	55.6	49.3	48.5	48.3	48.1
27/8/2022 14:15	0:15:00	1083	59	78.9	70.4	66.2	62.8	50.4	48.8	48.6	48.2
27/8/2022 14:30	0:15:00	1084	59.5	78.3	71.4	65.8	62.5	51.2	49	48.8	48.4
27/8/2022 14:45	0:15:00	1085	51.7	79.5	61.8	52.8	51	47.9	46.9	46.7	46.4
27/8/2022 15:00	0:15:00	1086	61.3	81.5	72.7	67.8	65	50.9	47.2	46.9	46.4
27/8/2022 15:15	0:15:00	1087	58.8	78.6	70.6	64.8	61.9	50	47.6	47.2	46.7
27/8/2022 15:30	0:15:00	1088	51.5	69.9	62.4	55.3	52.7	48.8	47.6	47.4	47.1
27/8/2022 15:45	0:15:00	1089	51.1	69.9	60.2	55.1	53	48.5	47.5	47.3	47
27/8/2022 16:00	0:15:00	1090	54.8	81.3	65.4	58.6	54.9	48.9	47.9	47.7	47.4
27/8/2022 16:15	0:15:00	1091	53.8	75.9	64.8	58.9	55.3	48.9	47.9	47.7	47.4
27/8/2022 16:30	0:15:00	1092	50.7	70.5	59.6	54.4	52.4	48.7	47.8	47.6	47.2
27/8/2022 16:45	0:15:00	1093	53.6	74	65.3	58.4	54.6	49	48	47.8	47.5
27/8/2022 17:00	0:15:00	1094	54.9	76.9	66.1	60	56.7	49.6	48.4	48.2	47.8
27/8/2022 17:15	0:15:00	1095	64.3	85.9	76	70.1	67.2	52.2	48.3	47.8	47.2
27/8/2022 17:30	0:15:00	1096	57.9	80	68.7	65	61.7	49.4	47.9	47.6	47.2

27/8/2022 17:45	0:15:00	1097	50.7	69.2	59.8	53.6	51.5	48.8	47.9	47.7	47.4
27/8/2022 18:00	0:15:00	1098	52.8	76.2	65.9	56	52.8	48.9	47.8	47.6	47.3
27/8/2022 18:15	0:15:00	1099	51.3	72.3	59.9	54.3	52.4	49	47.8	47.6	47.2
27/8/2022 18:30	0:15:00	1100	57.8	76.9	70.2	63.5	60.2	49.7	48	47.8	47.4
27/8/2022 18:45	0:15:00	1101	56.3	86.6	65.7	60	56.8	49.3	47.8	47.5	47.2
27/8/2022 19:00	0:15:00	1102	58.4	79.4	69.4	64.9	61.8	51.2	48.2	47.9	47.4
27/8/2022 19:15	0:15:00	1103	58.5	80.1	67.5	63.9	62	54.8	49.4	48.6	47.8
27/8/2022 19:30	0:15:00	1104	59.2	80	71.1	66	62.1	50	48.3	48	47.5
27/8/2022 19:45	0:15:00	1105	64.4	85.2	75.6	70.9	68.5	51.9	48.6	48.3	47.9
27/8/2022 20:00	0:15:00	1106	56.9	81	69.4	62.9	57.5	48.9	47.7	47.4	47.1
27/8/2022 20:15	0:15:00	1107	61.2	81.8	72.9	67.1	64	50.9	48.2	47.9	47.4
27/8/2022 20:30	0:15:00	1108	60.5	83.7	73.1	66.9	61.4	49.4	48.1	47.9	47.5
27/8/2022 20:45	0:15:00	1109	60.1	79.2	72.3	66.7	63.3	50.5	48.4	48.2	47.7
27/8/2022 21:00	0:15:00	1110	58.4	81.9	70.6	63.9	59.7	50.3	48.4	48.1	47.7
27/8/2022 21:15	0:15:00	1111	56.5	75.3	67.9	62.7	59.4	49.6	48	47.7	47.4
27/8/2022 21:30	0:15:00	1112	54.6	74.3	66.5	60.4	55	48.7	47.8	47.6	47.4
27/8/2022 21:45	0:15:00	1113	61.9	84	73.9	66.6	63.5	49	47.8	47.6	47.3
27/8/2022 22:00	0:15:00	1114	57.4	75.9	68.9	64.7	60.7	49	47.6	47.3	47
27/8/2022 22:15	0:15:00	1115	57.5	84.6	70.1	64.2	57.7	48.2	47.3	47.1	46.8
27/8/2022 22:30	0:15:00	1116	54.8	73.8	66.1	60.9	56.9	48.8	47.7	47.5	47.2
27/8/2022 22:45	0:15:00	1117	60.6	79.8	72.5	68.1	64.3	49.1	47.8	47.6	47.2
27/8/2022 23:00	0:15:00	1118	57.9	79.5	70.6	64.3	57.5	48.5	47.6	47.4	47.2
27/8/2022 23:15	0:15:00	1119	67.7	87.1	80.1	74.7	70.9	51.7	48.1	47.8	47.3
27/8/2022 23:30	0:15:00	1120	61	82.4	73.5	68.1	62	48.3	47.3	47.1	46.8
27/8/2022 23:45	0:15:00	1121	62	80.3	74.8	70.5	63.7	48.3	47.4	47.3	47
28/8/2022 0:00	0:15:00	1122	61.7	81.3	74.6	69.2	62.4	48.8	47.8	47.6	47.2
28/8/2022 0:15	0:15:00	1123	60.8	78.2	73.6	68.2	63	49.3	47.8	47.6	47.2
28/8/2022 0:30	0:15:00	1124	60.6	83.3	74.6	65.8	60.3	48.8	47.8	47.6	47.3
28/8/2022 0:45	0:15:00	1125	57.3	78.4	70.9	61.6	54.4	48.5	47.5	47.3	46.9
28/8/2022 1:00	0:15:00	1126	58.8	79.3	71	65.5	61.2	48.7	47.1	46.8	46.4
28/8/2022 1:15	0:15:00	1127	59.2	81.5	70.6	65.7	61.8	49.3	47.2	47	46.4
28/8/2022 1:30	0:07:49	1128	60.2	81.9	71.8	66.1	62.5	50.4	47.8	47.6	47.1
