# Supplementary material for: Mitigating Intensive Care Unit Noise: Design-Led Modeling Solutions, Calculated Acoustic Outcomes, and Cost Implications
Source: HERD. 2024 Mar 21;17(3):220–38. doi: 10.1177/19375867241237501 (PMC11457460; doi:10.1177/19375867241237501)
Supplement: Supplemental Material, sj-pdf-2-her-10.1177_19375867241237501 - Mitigating Intensive Care Unit Noise: Design-Led Modeling Solutions, Calculated Acoustic Outcomes, and Cost Implications [file sj-pdf-2-her-10.1177_19375867241237501.pdf]

FIELD NOISE LEVEL MEASUREMENTS

	FIONA STANLEY HOSPITAL - EQUIPMENT ALARMS	DATE/TIME	LENGTH	ENVIRONMENTAL NOISE LEVELS LA90	LA10	LA1	LAmax		OCTAVE FREQUENCY DATA - LEQ dB 31.5	63	125	250	500	1 K	2 K	4 K	8 K	LAEQ
**Project 100**	Phillips MX-800 Patient Monitor - Alarm at max volume (setting 10)	29/08/2022 10:33	00:00:08	66.5	71.7	73.3	73.4		50.9	44.6	45.9	40.4	64.7	56.8	68.2	42.2	24.4	**70.2**
	SPL at 1 metre			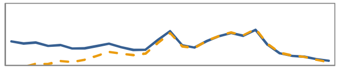				1/3 Octave	47.8	40.3	36.2	38.0	50.0	37.6	61.3	42.0	22.3	
									44.3	41.6	39.9	33.7	**64.5**	48.4	56.8	27.8	18.3	
									45.6	36.0	43.9	34.0	41.3	56.1	**66.8**	23.7	15.6	
**Project 101**	Phillips MX-800 Patient Monitor - Alarm at medium volume (setting 5)	29/08/2022 10:33	00:00:11	52.5	57.3	57.7	57.6		51.1	46.4	42.3	39.7	52.9	45.0	52.8	33.2	25.4	**55.6**
	SPL at 1 metre			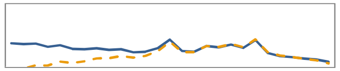				1/3 Octave	47.0	41.5	37.2	37.3	39.1	33.5	44.8	31.5	22.3	
									45.6	43.7	38.7	32.5	**52.7**	42.6	39.4	26.2	20.8	
									46.2	37.9	36.5	33.3	34.6	40.5	**51.8**	24.5	17.3	
**Project 102**	Phillips MX-800 Patient Monitor - Alarm at low volume (setting 2)	29/08/2022 10:34	00:00:08	46.3	49.7	51.3	51.5		51.3	45.6	43.5	39.8	45.7	42.9	44.6	29.7	23.3	**48.8**
	SPL at 1 metre			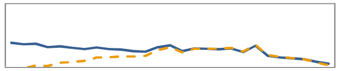				1/3 Octave	47.7	40.7	37.8	36.8	40.7	38.7	38.5	26.8	21.3	
									45.5	42.0	40.3	34.2	43.6	38.2	32.7	24.1	17.3	
									46.1	39.6	37.7	33.5	34.3	37.5	**43.0**	22.8	14.2	
**Project 103**	Hamilton S1 Ventilator - Alarm at maximum volume (setting 10)	29/08/2022 10:46	00:00:04	71.2	75.5	75.8	75.8		53.2	42.5	42.5	42.1	54.4	64.8	67.8	68.6	45.6	**73.5**
	SPL at 1 metre			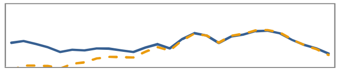				1/3 Octave	47.9	40.7	35.6	35.8	45.5	**63.2**	57.8	67.0	44.4	
									50.4	32.8	38.6	32.8	38.7	59.2	60.9	63.1	38.7	
									45.7	36.5	38.4	40.2	53.7	47.4	66.3	52.4	30.6	
**Project 104**	Hamilton S1 Ventilator - Alarm at medium volume (setting 5)	29/08/2022 10:46	00:00:07	52.8	55.4	56.8	58.2		52.6	42.0	44.8	43.7	51.7	46.6	49.2	48.7	31.6	**55.2**
	SPL at 1 metre			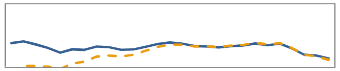				1/3 Octave	47.2	39.6	36.5	36.6	45.9	42.7	42.2	43.8	28.6	
									49.9	31.6	41.6	37.1	48.2	42.1	43.4	46.3	27.4	
									45.1	37.2	40.6	41.4	46.4	40.5	46.5	38.7	22.7	
**Project 105**	Carefusion Alaris Infusion Pump - Alarm at max setting	29/08/2022 10:48	00:00:10	64.4	70.7	71.9	72.0		51.5	44.5	41.4	44.6	47.9	56.3	65.7	45.1	45.2	**67.7**
	(Volume did not appear to be adjustable)			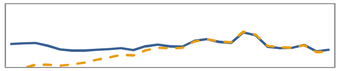				1/3 Octave	45.7	42.9	35.2	39.3	44.9	50.8	47.7	41.6	44.1	
	SPL at 1 metre								46.9	37.1	36.6	36.1	42.1	53.5	**64.4**	39.4	34.0	
									47.3	35.3	37.6	42.2	41.6	49.3	59.6	39.5	36.5	
**Project 106**	Cisco Phone ringing - maximum ring volume	29/08/2022 10:50	00:00:06	54.0	78.3	78.6	78.6		54.5	42.6	48.1	45.8	60.2	55.8	75.0	39.7	26.2	**76.1**
	SPL at 1 metre			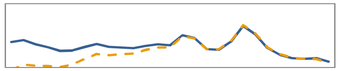				1/3 Octave	49.0	40.7	40.9	40.4	45.2	55.7	49.7	39.3	22.0	
									52.1	34.8	45.9	39.0	43.6	37.5	**74.8**	28.6	23.0	
									45.6	35.5	41.4	42.8	59.9	36.7	61.2	23.2	17.6	
**Project 107**	Cisco phone ringing - medium ring volume	29/08/2022 10:50	00:00:06	64.9	67.4	67.8	67.9		52.8	42.6	43.7	44.5	51.3	49.8	65.1	32.0	25.1	**66.4**
	SPL at 1 metre			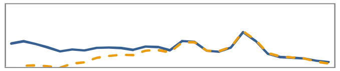				1/3 Octave	46.6	40.4	35.5	39.5	41.0	49.5	40.3	30.2	22.9	
									50.3	33.9	39.7	36.5	35.9	35.2	**64.9**	24.8	19.0	
									46.1	36.9	40.2	41.8	50.7	33.7	51.0	23.8	16.9	
