# Supplementary material for: Mitigating Intensive Care Unit Noise: Design-Led Modeling Solutions, Calculated Acoustic Outcomes, and Cost Implications
Source: HERD. 2024 Mar 21;17(3):220–38. doi: 10.1177/19375867241237501 (PMC11457460; doi:10.1177/19375867241237501)
Supplement: Supplemental Material, sj-pdf-3-her-10.1177_19375867241237501 - Mitigating Intensive Care Unit Noise: Design-Led Modeling Solutions, Calculated Acoustic Outcomes, and Cost Implications [file sj-pdf-3-her-10.1177_19375867241237501.pdf]

LEGEND

CWT50	MATERIAL / ITEM - REFER LEGEND
PNT02-01	SELECTED FINISH - REFER SCHEDULES

MATERIAL / ITEM SCHEDULE

TAG	DESCRIPTION
APW001	40mm THICK WALL PANEL
APW002	40mm THICK CORNICE
CFB001	CURTAIN WITH ANTIMICROBIAL COATING
CSU001	SUSPENDED GRID ACOUSTIC TILE CEILING
RES001	ACOUSTIC SHEET VINYL WITH UNDERLAY
RES003	COVED VINYL SKIRTING
RES004	ACOUSTIC WALL VINYL

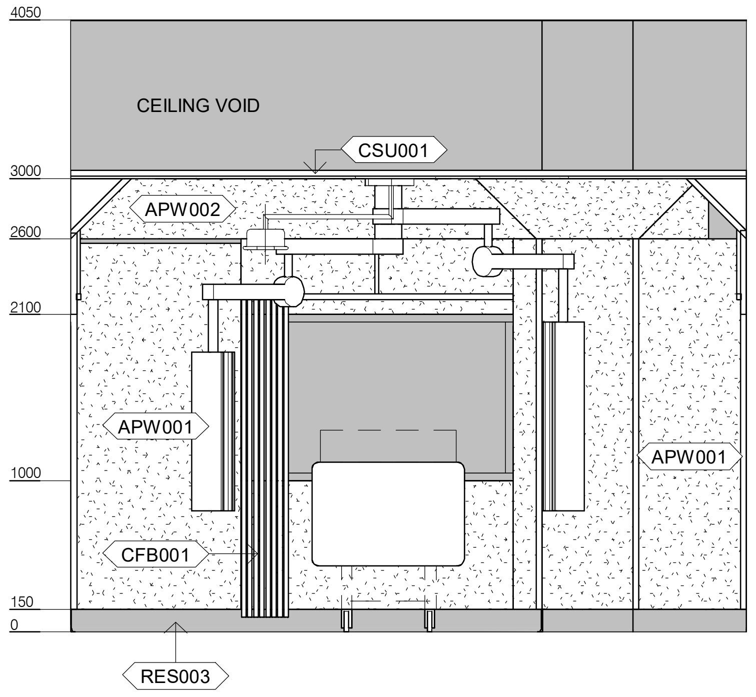

ELEVATION A
1 : 50

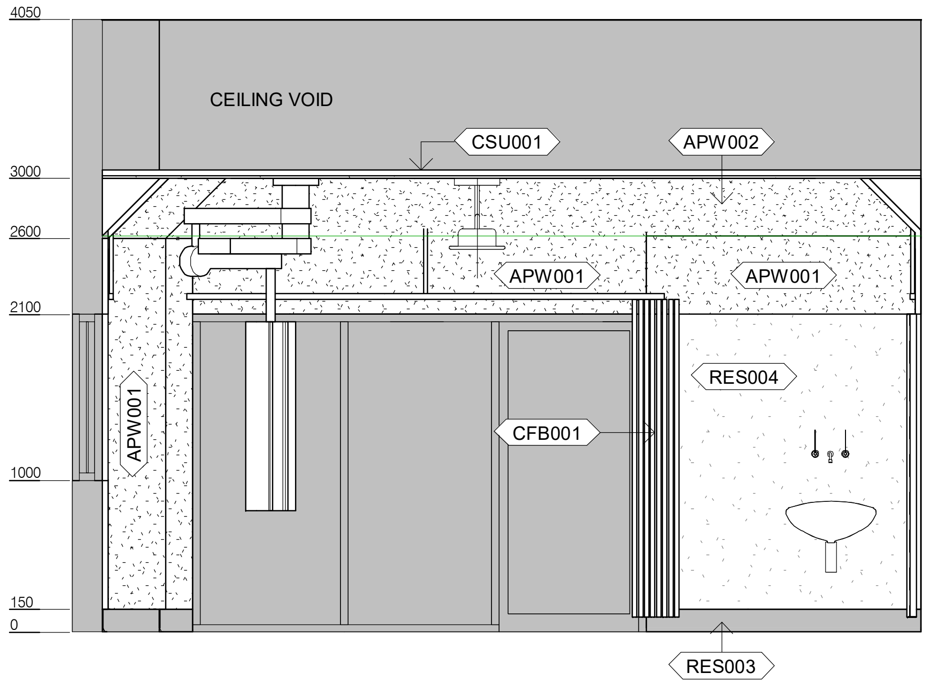

ELEVATION B
1 : 50

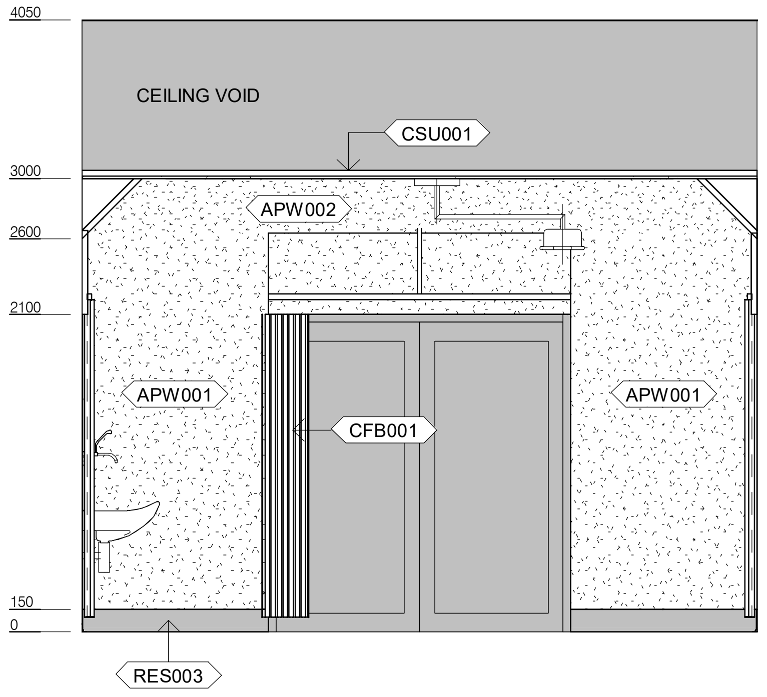

ELEVATION C
1 : 50

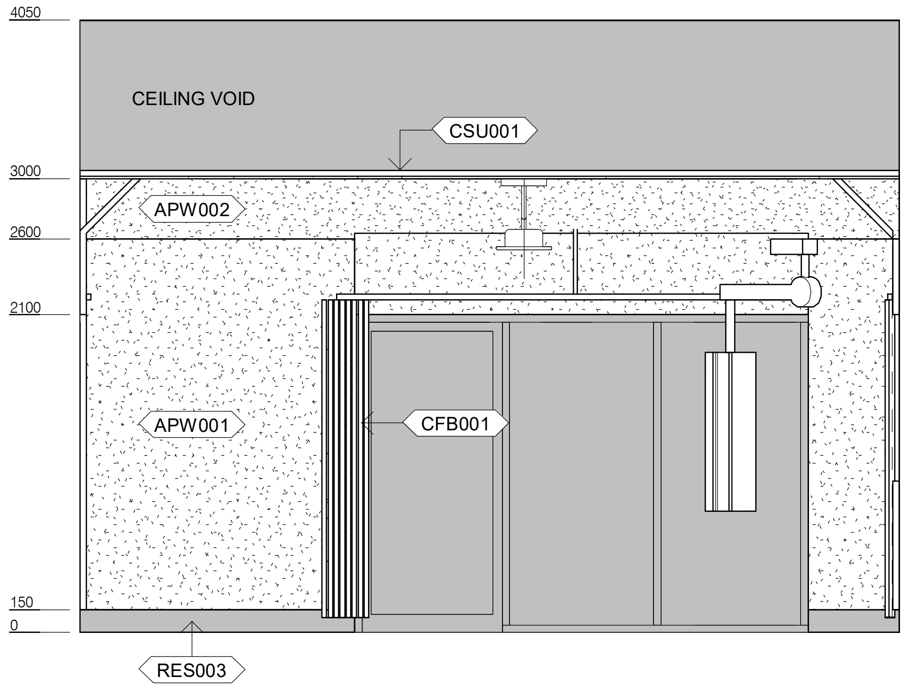

ELEVATION D
1 : 50

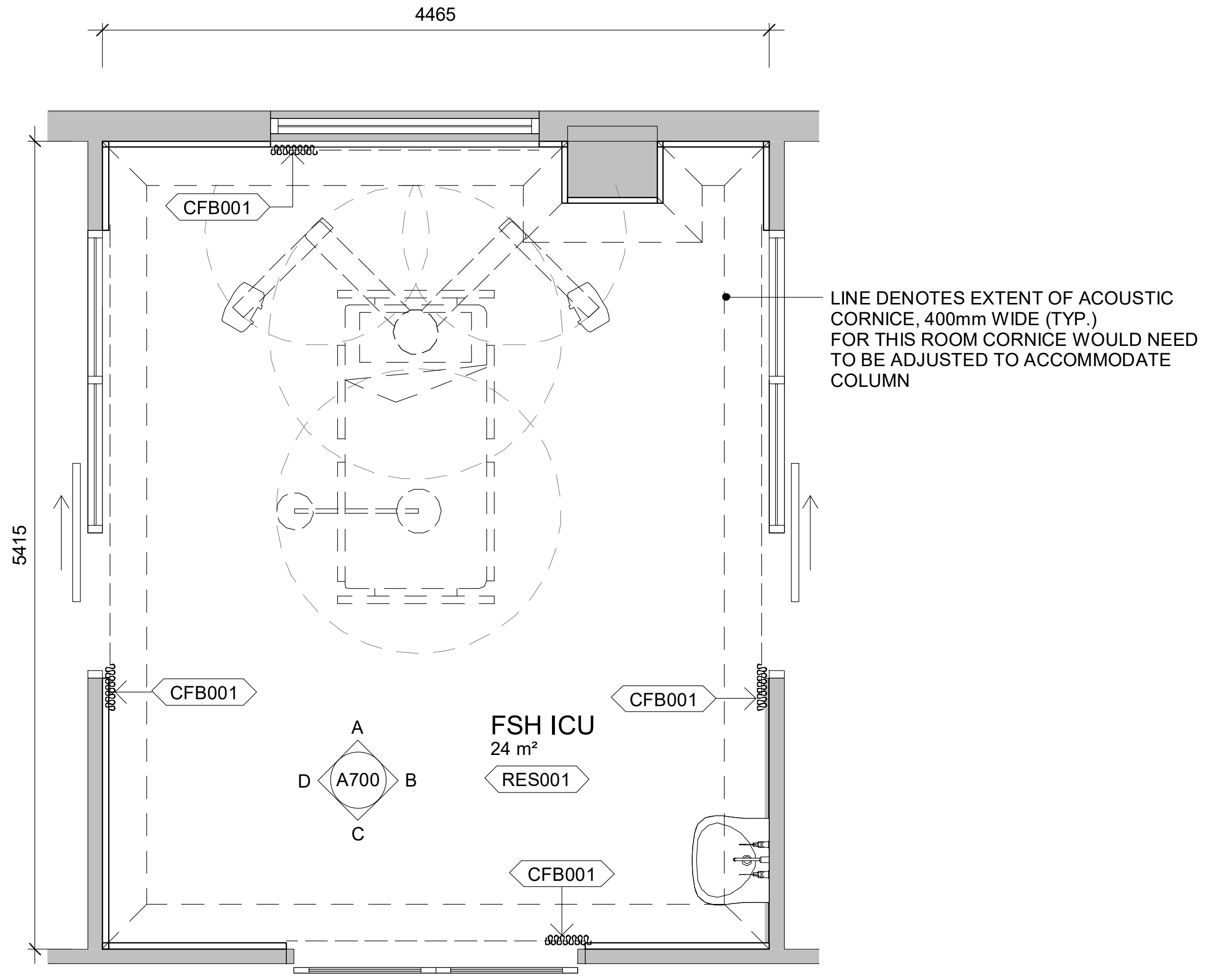

FLOOR PLAN
1 : 50

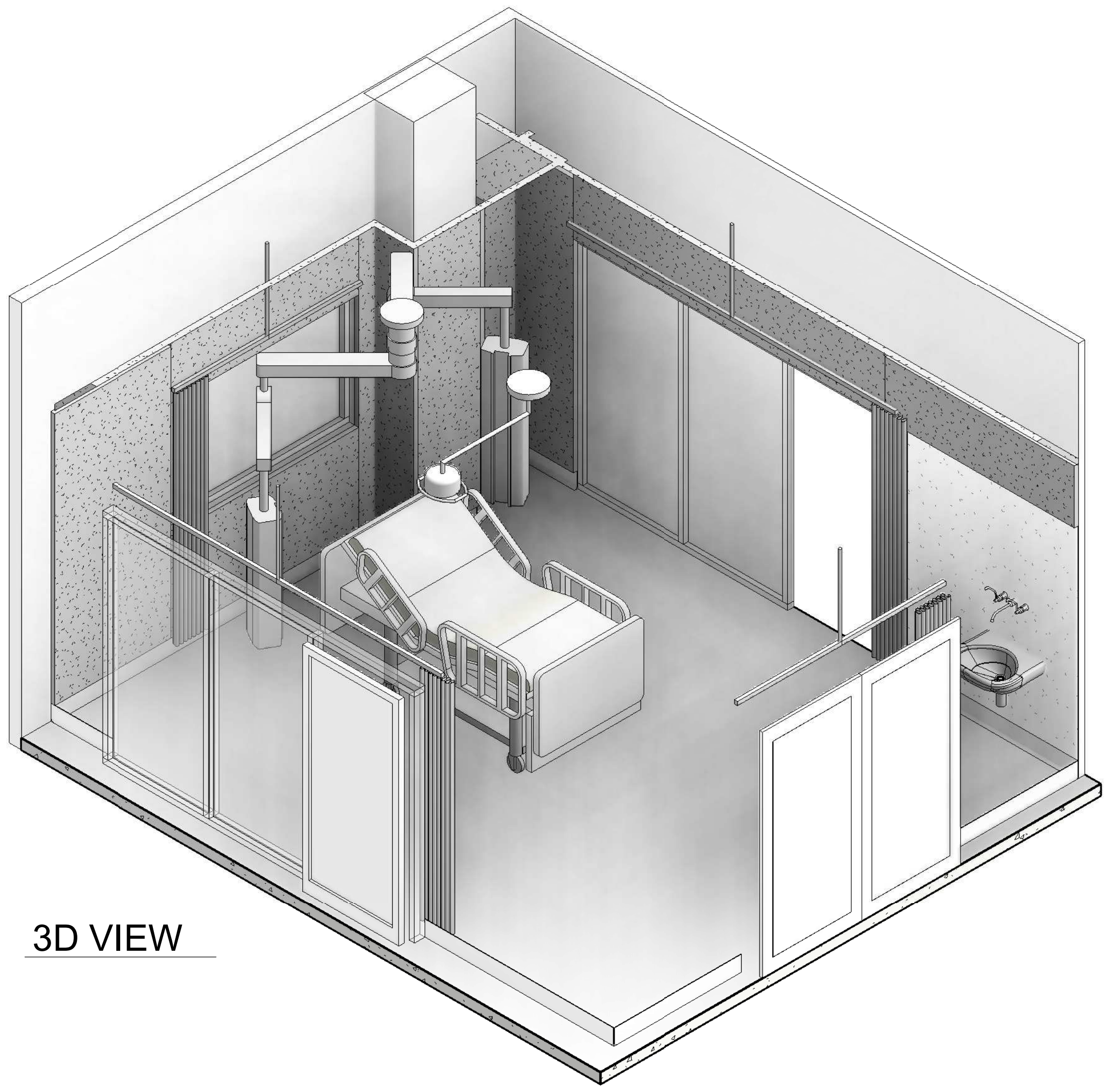

3D VIEW

ROOM LAYOUT - FSH ICU BED (OPTION A)

FSH ICU NOISE STUDY - FSH QA 46840

Status: PRELIMINARY
Path:

Scale: 1:50 @ A2
0 3

© Hames Sharley:

Project Number: 46840
Drawing Number: A700
Revision:
Date:

HAMES SHARLEY
