# Supplementary material for: Mitigating Intensive Care Unit Noise: Design-Led Modeling Solutions, Calculated Acoustic Outcomes, and Cost Implications
Source: HERD. 2024 Mar 21;17(3):220–38. doi: 10.1177/19375867241237501 (PMC11457460; doi:10.1177/19375867241237501)
Supplement: Supplemental Material, sj-pdf-4-her-10.1177_19375867241237501 - Mitigating Intensive Care Unit Noise: Design-Led Modeling Solutions, Calculated Acoustic Outcomes, and Cost Implications [file sj-pdf-4-her-10.1177_19375867241237501.pdf]

LEGEND

CWT50 — MATERIAL / ITEM - REFER LEGEND

PNT02-01 — SELECTED FINISH - REFER SCHEDULES

MATERIAL / ITEM SCHEDULE

TAG	DESCRIPTION
APW001	40mm THICK ECOPHON HYGIENE PEFORMANCE CARE WALL PANEL
APW002	40mm THICK ECOPHON HYGIENE PEFORMANCE CARE WALL PANEL 400mm CORNICE
CFB001	HEMP CURTAIN WITH ANTIMICROBIAL COATING
CSU001	ECOPHON HYGIENE PROTEC Ds SUSPENDED GRID ACOUSTIC TILE CEILING
RES001	ECOSURFACES Aurora Rx SHEET VINYL WITH 5mm VULCANISED COMPOSITION RUBBER BASE LAYER
RES003	150mm H COVED VINYL SKIRTING
RES004	ACOUSTIC WALL VINYL

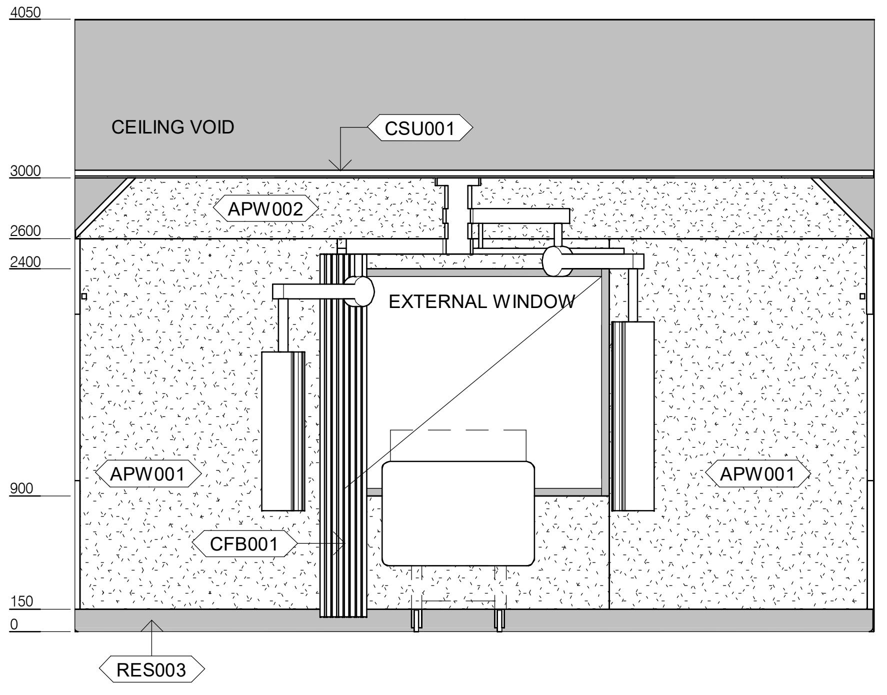

ELEVATION A
1 : 50

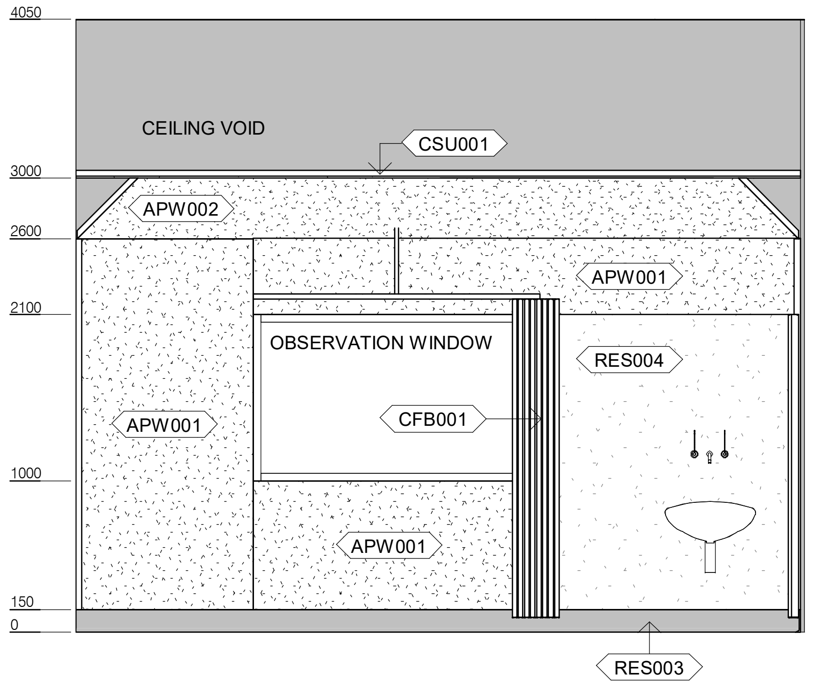

ELEVATION B
1 : 50

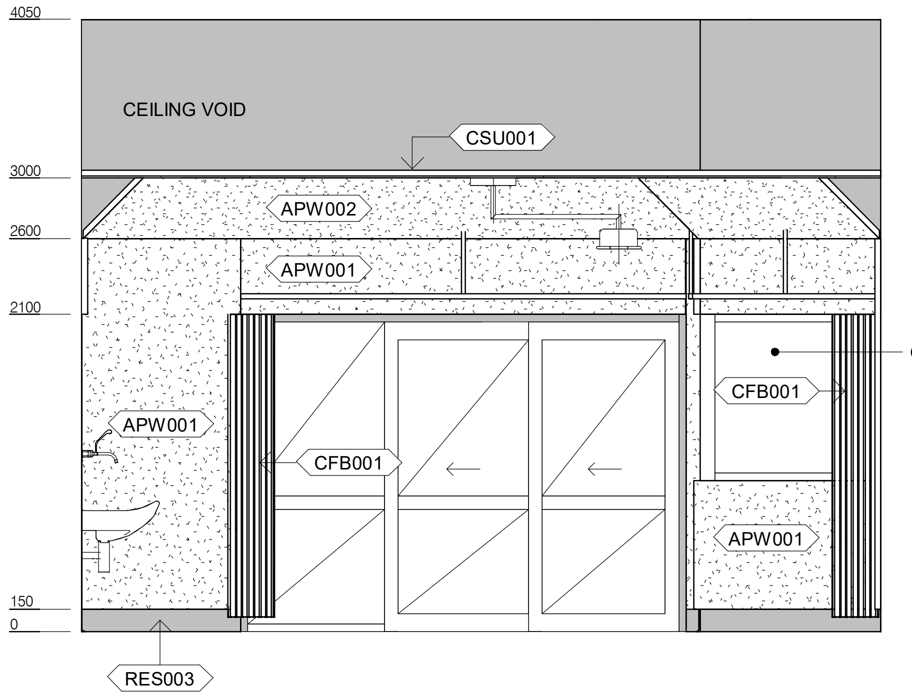

ELEVATION C
1 : 50

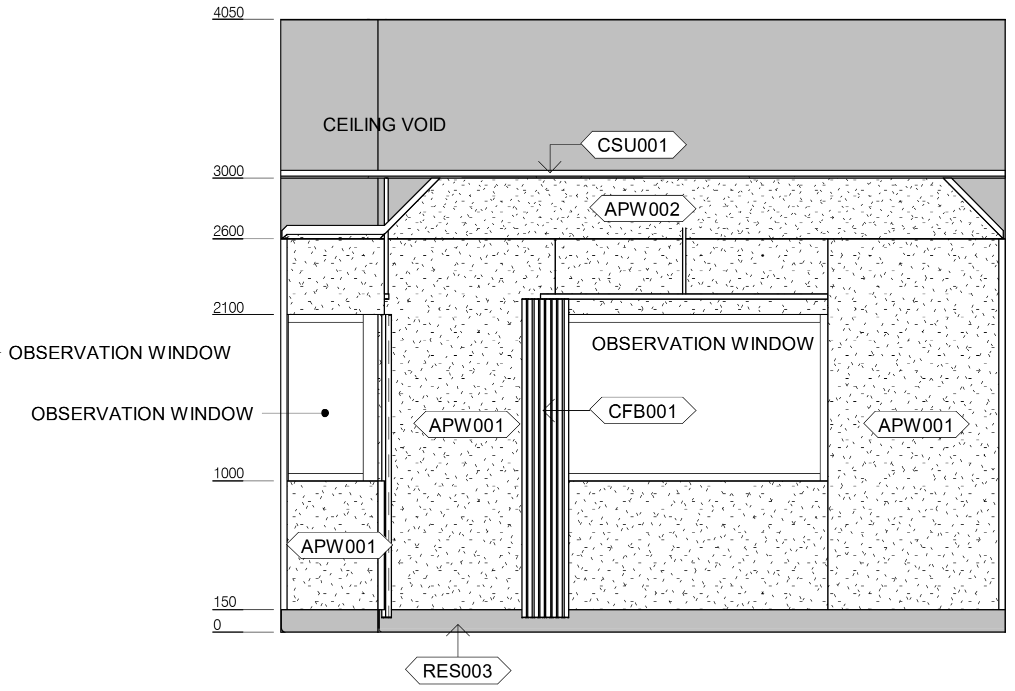

ELEVATION D
1 : 50

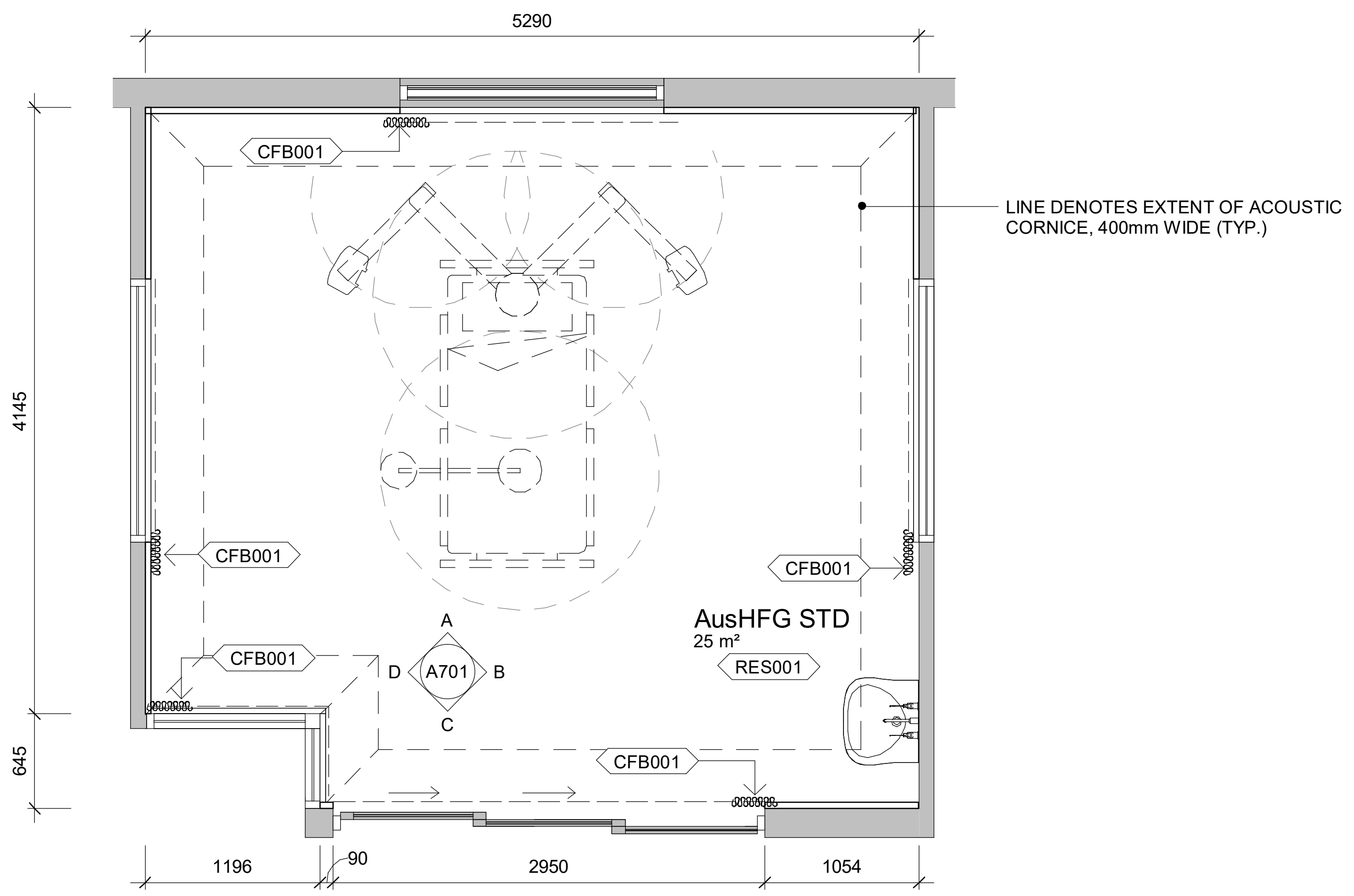

FLOOR PLAN
1 : 50

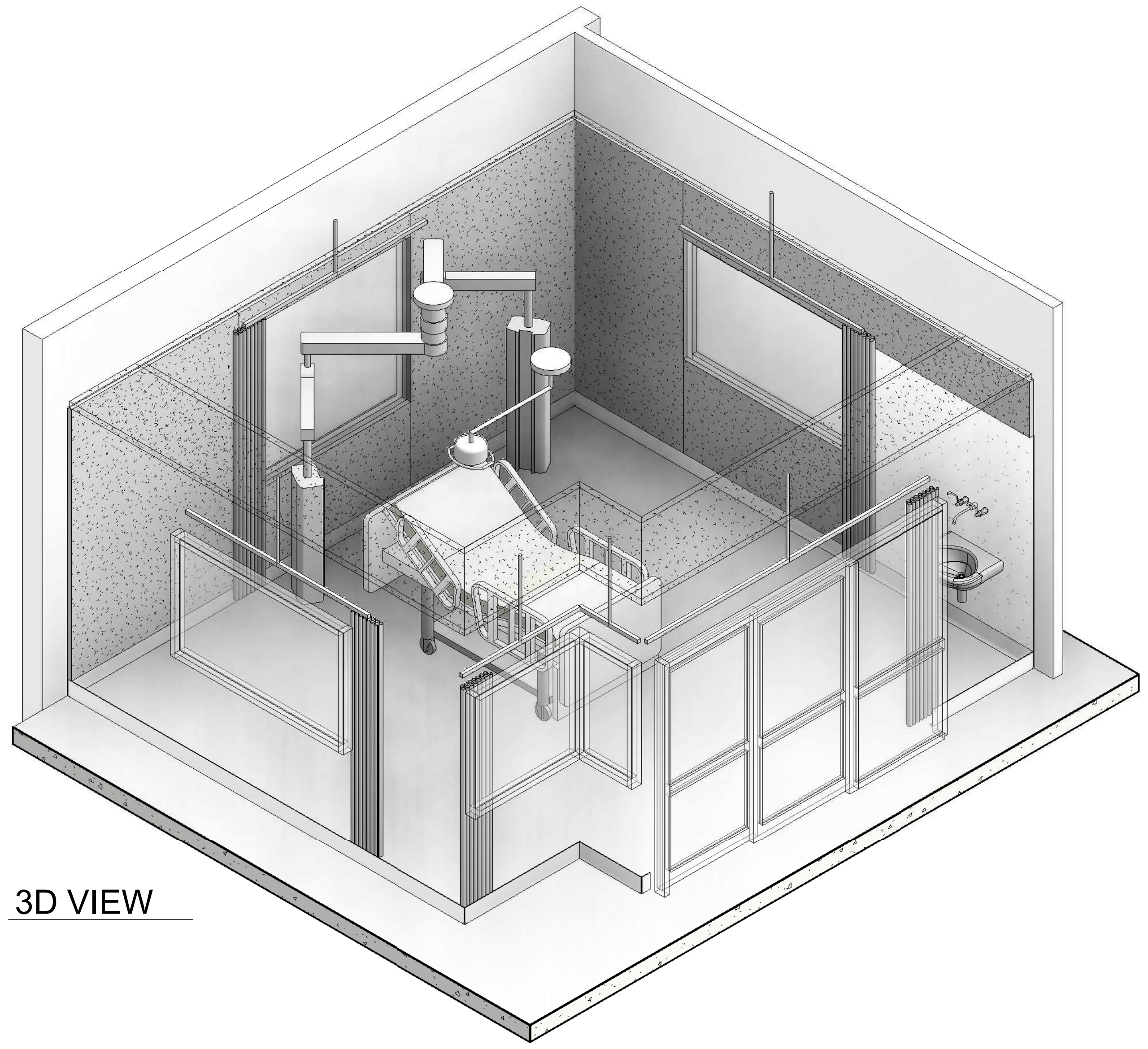

3D VIEW

ROOM LAYOUT - AusHFG ICU BED (OPTION B)
FSH ICU NOISE STUDY - FSH QA 46840

Status: PRELIMINARY
Path:

Scale: 1:50 @ A2
0 — 3

© Hames Sharley:

Project Number: 46840
Drawing Number: A701
Revision:
Date:

Hames Sharley
