# Supplementary material for: Mitigating Intensive Care Unit Noise: Design-Led Modeling Solutions, Calculated Acoustic Outcomes, and Cost Implications
Source: HERD. 2024 Mar 21;17(3):220–38. doi: 10.1177/19375867241237501 (PMC11457460; doi:10.1177/19375867241237501)
Supplement: Supplemental Material, sj-pdf-6-her-10.1177_19375867241237501 - Mitigating Intensive Care Unit Noise: Design-Led Modeling Solutions, Calculated Acoustic Outcomes, and Cost Implications [file sj-pdf-6-her-10.1177_19375867241237501.pdf]

Variable	ID	Experience_Healthcare	Experience_ICU	Current_Role	ICUP_01	ICUP_02	ICUP_03	ICUP_04	ICUP_05	ICUP_06	ICUP_07	ICUP_08	ICUP_09	ICUP_10	ICUP_11
Label	Participant's ID	Experience in the health profession (Years)	Years of ICU experience	Current designation or role	The way in which the ICU is designed helps me to perform my duties.	The ICU allows me to communicate efficiently with other staff in the ICU.	I am able to communicate safely and effectively without disrupting patient sleep.	Noise levels in the ICU negatively impact on clinical care.	Noise levels in the ICU negatively impact staff wellbeing.	Impact of noise in the ICU is different to other areas such as Trauma and Emergency.	Alarms are a significant source of noise in the ICU.	Talking between clinicians is a significant source of noise in the ICU.	Patient procedures are a significant source of noise in the ICU.	Managing ventilation and life support equipment is a significant source of noise in the ICU.	Visitors such as family members and/or ancillary staff is a significant source of noise in the ICU.
	1	25	3	1	5	2	1	7	6	2	6	7	5	6	6
	2	25	4	2	4	5	4	6	5	5	7	6	1	2	5
	3	15	1	2	6	6	4	5	5	5	6	5	5	6	5
	4	35	4	2	4	5	3	5	5	6	5	4	3	3	5
	5	36	4	2	3	3	3	6	5	4	6	4	2	4	7
	6	3	1	3	6	6	6	6	6	3	6	2	2	4	4
	7	35	4	1	2	2	2	6	5	5	6	6	5	6	6
	8	28	3	3	6	6	6	7	6	6	7	5	5	6	4
	9	30	4	1	6	5	5	7	6	5	6	6	4	5	4
	10	7	1	4	6	5	5	6	5	6	5	6	5	5	5
	11	23	4	1	6	6	7	6	6	5	6	6	2	2	2
	12	22	3	1	4	4	6	7	6	6	7	6	2	2	6
	13	8	2	3	6	6	5	5	4	7	2	6	2	2	2
	14	30	4	1	4	4	4	6	4	6	7	6	5	6	4
	15	32	4	1	6	6	5	6	6	6	5	5	6	5	4
	16	22	4	2	6	5	6	5	4	4	6	6	3	5	3
	17	3	1	3	6	6	5	6	5	5	7	4	3	6	3
	18	20	3	1	4	5	4	5	4	2	6	5	5	6	5
	19	18	2	1	5	6	4	5	2	2	7	1	2	5	5
	20	3	1	3	6	5	4	4	2	5	6	5	4	5	4
	21	26	4	1	6	5	7	6	3	6	5	5	4	5	4
	22	7	2	4	2	3	3	7	1	4	7	6	7	7	5
	23	12	3	1	5	3	6	4	2	6	5	3	2	4	2
	24	30	3	4	3	3	4	5	2	6	6	2	2	2	5
	25	10	2	3	3	4	1	7	5	3	5	6	5	6	3
	26	10	1	4	6	5	3	7	5	4	6	5	5	3	5
	27	17	3	3	6	6	6	6	6	6	7	4	4	4	4
	28	15	3	3	5	6	6	7	6	6	6	5	3	3	4
	29	10	2	3	5	5	3	3	4	5	7	6	5	6	5
	30	13	2	1	5	4	3	5	3	4	5	3	2	2	2
	31	13	1	2	5	6	5	6	4	6	5	5	3	4	6
	32	13	1	2	5	6	6	4	3	6		6	3	3	6
	33	19	3	1	5	6	6	6	6	6	5	4	5	6	6
	34	3	1	4	7	7	6	6	2	6	7	5	3	5	2
	35	8	2	2	5	4	5	4	3	4	6	5	4	5	4
	36	2	1	4	4	5	3	5	4		7	6	5	2	6
	37	7	1	2	1	1	1	7	7	7	7	4	4	4	7
	38	5	1	5	3	2	2	5	1	6	6	6	6	6	6
	39	14	3	3	2	3	2	6	6	3	6	7	5	6	5
	40	1	1	2	6	6	3	3	3	5	2	5	5	5	5
	41	25	3	1	6	5	4	5	5	5	6	6	6	6	6
	42	3	1	3	6	6	5	5	4	4	6	5	4	6	4
	43	22	3	1	6	6	3	6	5	5	5	6	5	5	5
	44	9	2	5	3	4	4	5	4	6	6	5	4	5	4
	45	7	2	2	4	4	4	4		4	4	4	4	4	4
	46	1	1	2	6	5	6	2	2	6	5	4	2	2	2
	47	20	4	5	6	6	5	6	6	2	7	7	6	6	5
	48	6	2	4	6	4	3	7	4	7	6	5	5	5	5
	49	7	2	5	6	4	5	6	1	4	7	6	5	5	5
	50	12	1	6	6	6	5	1	2	7	3	1	1	5	1
	51	5	1	4	6	6	6	3	2	4	7	5	5	5	2
	52	7	1	3	6	5	5	5	4	6	5	4	6	4	5
	53	25	3	1	5	6	5	6	4	6	7	6	6	3	6
	54	1	1	4	5	6	5	5	4	6	7	3	2	7	3
	55	9	1	3						5	7	5	4	7	3
	56	36	4	5	6	7	7	5	4	5	5	5	4	2	5
	57	3	1	6	7	6	6	3	2	6	6	5	5	5	5
	58	15	3	7	3	3	3	5	4	2	3	5	5	5	4
	59	40	2	7	6	6	6	5	4	7	6	6	6	6	6
	60	28	2	5	5	5	2	7	7	6	7		6	6	7

61	22	3	1	5	5	4	5	5	2	6	6	5	5	5
62	16	3	5	3	4	6	6	5	4	5	5	6	5	5
63	13		5	4	3	4	6	4	6	5	6	4	5	6
64	6	1	4	5	3	4	7	3	7	6	6	6	5	3
65	12	3	4	6	2	4	3	3	6	7	6	6	4	5
66	3	1	3	6	5	5	3	4	6	4	2	2	3	3
67	22	4	2	6	6	6	5	5	6	5	5	2	2	2
68	8	1	3	4	2	2	7	7	7	7	7	7	7	5
69	12	2	3	5	6	4	6	5	5	6	5	5	5	4
70	10	2	3	3	2	4	6	6	4	6	3	1	1	6
71	4	1	4	6	6	4	3	4	5	6	5	4	4	5
72	15	2	5	3	5	2	6	4	7	7	5	5	7	4
73	8	1	4	5	4	4	6	4	4	5	5	4	4	4
74	11	1	3	7	7	5	5	3	6	6	4	4	2	4

ICUP_12<br>I believe a 'quieter' space provides clinicians with an increased ability to think.	ICUP_13<br>I believe the quality of my work is negatively impacted by a noisy environment.	ICUP_14<br>I believe noise could contribute to medical errors.	ICUP_15<br>I believe noise decreases my sense of connection with patients.	ICUP_16<br>I believe noise decreases staff sense of satisfaction.	ICUP_17<br>I believe noise negatively impacts on patient sleep quality in the ICU.
7	7	5	5	6	7
7	7	7	5	6	7
5	2	5	5	4	6
5	5	6	6	6	6
7	6	4	4	6	7
7	7	6	6	5	7
5	5	5	4	5	7
7	6	7	7	6	6
5	6		6	6	6
5	4	4	5	4	6
7	6	6	6	6	6
7	7	7	7	6	7
2	2	3	2	5	5
6	5	4	5	5	7
7	6	6	5	6	7
6	3	3	3	4	6
5	3	3	3	3	7
6	5	6	6	5	6
7	6	7	5	2	7
4	3	6	3	3	6
5	5	5	5	5	7
5	6	6	3	1	7
5	5	5	2	4	7
5	2	6	3	3	6
5	5	6	3	6	7
6	5	7	3	4	7
6	6	6	6	6	6
6	6	5	5	2	6
4	5	4	5	4	7
4	2	3	5	3	5
5	5	5	6	5	5
6		5	5	5	6
6	6	6	6	5	7
6	5	5	2	5	7
6	6	6	6	4	6
6	6	6	5	4	7
7	7	6	6	6	7
6	4	5	4	6	6
7	7	7	6	7	7
5	1	4	4	4	6
6	6	6	6	5	7
6	5	6	5	4	7
5	5	5	5	5	6
5	6	6	6	6	7
5	5	5	5	5	5
5	2	6	5	5	6
7	5	6	5	4	7
6	6	6	5	6	7
4	3	4	4	4	7
6	6	4	2	5	5
6	4	4	4	4	5
4	3	5	5	4	7
6	6	6	4	5	7
6	4	6	6	6	7
6	6	4	4	4	7
6	4	7	4	5	7
5	6	6	6	5	6
5	5	5	5	5	5
6	6	7	6	6	7
7	7	7	7	7	7

5	5	5	4	5	5
6	4	5	3	4	7
7	5	6	5	4	7
6	5	6	3	3	7
5	5	5	5	5	5
6	4	6	5	4	4
7	7	6	3	5	6
7	7	7	7	7	7
7	6	6	6	6	6
6	7	6	4	6	7
6	4	5	5	4	7
7	6	7	6	4	7
5	5	5	5	5	6
6	3	5	5	5	7

<table>
<tr><td>Variable</td><td>ID</td><td>Experience_Healthcare</td><td>Experience_ICU</td><td>Current_Role</td><td>ICUP_01</td><td>ICUP_02</td><td>ICUP_03</td><td>ICUP_04</td><td>ICUP_05</td><td>ICUP_06</td><td>ICUP_07</td><td>ICUP_08</td><td>ICUP_09</td><td>ICUP_10</td><td>ICUP_11</td></tr>
<tr><td>Question</td><td>Participant's ID</td><td>Experience in the health profession (Years)</td><td>Years of ICU experience</td><td>Current designation or role</td><td>The way in which the ICU is designed helps me to perform my duties.</td><td>The ICU allows me to communicate efficiently with other staff in the ICU.</td><td>I am able to communicate safely and effectively without disrupting patient sleep.</td><td>Noise levels in the ICU negatively impact on clinical care.</td><td>Noise levels in the ICU negatively impact staff wellbeing.</td><td>Impact of noise in the ICU is different to other areas such as Trauma and Emergency.</td><td>Alarms are a significant source of noise in the ICU.</td><td>Talking between clinicians is a significant source of noise in the ICU.</td><td>Patient procedures are a significant source of noise in the ICU.</td><td>Managing ventilation and life support equipment is a significant source of noise in the ICU.</td><td>Visitors such as family members and/or ancillary staff is a significant source of noise in the ICU.</td></tr>
<tr><td>Subquestion</td><td></td><td></td><td></td><td></td><td></td><td></td><td></td><td></td><td></td><td></td><td></td><td></td><td></td><td></td><td></td></tr>
<tr><td>Scale</td><td>Nominal</td><td>Nominal</td><td>Nominal</td><td>Nominal</td><td>Scale</td><td>Scale</td><td>Scale</td><td>Scale</td><td>Scale</td><td>Scale</td><td>Scale</td><td>Scale</td><td>Scale</td><td>Scale</td><td>Scale</td></tr>
<tr><td>Missings</td><td></td><td></td><td></td><td></td><td></td><td></td><td></td><td></td><td></td><td></td><td></td><td></td><td></td><td></td><td></td></tr>
<tr><td>Values/Labels</td><td></td><td></td><td>1 | <5
2 | 6-9
3 | 10-19
4 | 20+</td><td>1 | Consultant
2 | Allied health professi
3 | Registrar
4 | RN
5 | CN
6 | RMO
7 | CNS/NUM</td><td>1 | Strongly Disagree
2 | Disagree
3 | Somewhat Disagree
4 | Neither Agree Nor Dis
5 | Somewhat Agree
6 | Agree
7 | Strongly Agree</td><td>1 | Strongly Disagree
2 | Disagree
3 | Somewhat Disagree
4 | Neither Agree Nor Dis
5 | Somewhat Agree
6 | Agree
7 | Strongly Agree</td><td>1 | Strongly Disagree
2 | Disagree
3 | Somewhat Disagree
4 | Neither Agree Nor Dis
5 | Somewhat Agree
6 | Agree
7 | Strongly Agree</td><td>1 | Strongly Disagree
2 | Disagree
3 | Somewhat Disagree
4 | Neither Agree Nor Dis
5 | Somewhat Agree
6 | Agree
7 | Strongly Agree</td><td>1 | Strongly Disagree
2 | Disagree
3 | Somewhat Disagree
4 | Neither Agree Nor Dis
5 | Somewhat Agree
6 | Agree
7 | Strongly Agree</td><td>1 | Strongly Disagree
2 | Disagree
3 | Somewhat Disagree
4 | Neither Agree Nor Dis
5 | Somewhat Agree
6 | Agree
7 | Strongly Agree</td><td>1 | Strongly Disagree
2 | Disagree
3 | Somewhat Disagree
4 | Neither Agree Nor Dis
5 | Somewhat Agree
6 | Agree
7 | Strongly Agree</td><td>1 | Strongly Disagree
2 | Disagree
3 | Somewhat Disagree
4 | Neither Agree Nor Dis
5 | Somewhat Agree
6 | Agree
7 | Strongly Agree</td><td>1 | Strongly Disagree
2 | Disagree
3 | Somewhat Disagree
4 | Neither Agree Nor Dis
5 | Somewhat Agree
6 | Agree
7 | Strongly Agree</td><td>1 | Strongly Disagree
2 | Disagree
3 | Somewhat Disagree
4 | Neither Agree Nor Dis
5 | Somewhat Agree
6 | Agree
7 | Strongly Agree</td><td>1 | Strongly Disagree
2 | Disagree
3 | Somewhat Disagree
4 | Neither Agree Nor Dis
5 | Somewhat Agree
6 | Agree
7 | Strongly Agree</td></tr>
</table>

ICUP_12	ICUP_13	ICUP_14	ICUP_15	ICUP_16	ICUP_17
I believe a 'quieter' space provides clinicians with an increased ability to think.<br><br>Scale<br><br>1 | Strongly Disagree<br>2 | Disagree<br>3 | Somewhat Disagree<br>4 | Neither Agree Nor Dis<br>5 | Somewhat Agree<br>6 | Agree<br>7 | Strongly Agree	I believe the quality of my work is negatively impacted by a noisy environment.<br><br>Scale<br><br>1 | Strongly Disagree<br>2 | Disagree<br>3 | Somewhat Disagree<br>4 | Neither Agree Nor Dis<br>5 | Somewhat Agree<br>6 | Agree<br>7 | Strongly Agree	I believe noise could contribute to medical errors.<br><br>Scale<br><br>1 | Strongly Disagree<br>2 | Disagree<br>3 | Somewhat Disagree<br>4 | Neither Agree Nor Dis<br>5 | Somewhat Agree<br>6 | Agree<br>7 | Strongly Agree	I believe noise decreases my sense of connection with patients.<br><br>Scale<br><br>1 | Strongly Disagree<br>2 | Disagree<br>3 | Somewhat Disagree<br>4 | Neither Agree Nor Dis<br>5 | Somewhat Agree<br>6 | Agree<br>7 | Strongly Agree	I believe noise decreases staff sense of satisfaction.<br><br>Scale<br><br>1 | Strongly Disagree<br>2 | Disagree<br>3 | Somewhat Disagree<br>4 | Neither Agree Nor Dis<br>5 | Somewhat Agree<br>6 | Agree<br>7 | Strongly Agree	I believe noise negatively impacts on patient sleep quality in the ICU.<br><br>Scale<br><br>1 | Strongly Disagree<br>2 | Disagree<br>3 | Somewhat Disagree<br>4 | Neither Agree Nor Dis<br>5 | Somewhat Agree<br>6 | Agree<br>7 | Strongly Agree

#	Content	Reference	Text	Comment
1	QUESTION	[...]	**Participant's ID**	
	VARIABLES	ID		
	VALUES			
2	QUESTION	[...]	**Experience in the health profession (Years)**	
	VARIABLES	Experience_Healthcare		
	VALUES			
3	QUESTION	[...]	**Years of ICU experience**	
	VARIABLES	Experience_ICU		
	VALUES	1	<5	
		2	6-9	
		3	10-19	
		4	20+	
4	QUESTION	[...]	**Gender**	
	VARIABLES	Gender		
	VALUES	1	Male	
		2	Female	
		3	Non-binary	
		4	Prefer not to say	
5	QUESTION	[...]	**Current designation or role**	
	VARIABLES	Current_Role		
	VALUES	1	Consultant	
		2	Allied health professional	
		3	Registrar	
		4	RN	
		5	CN	
		6	RMO	
		7	CNS/NUM	
6	QUESTION	[...]	**[...]**	
	VARIABLES	ICUP_01	The way in which the ICU is designed helps me to perform my duties.	
		ICUP_02	The ICU allows me to communicate efficiently with other staff in the ICU.	
	VALUES	1	Strongly Disagree	
		2	Disagree	
		3	Somewhat Disagree	
		4	Neither Agree Nor Disagree	
		5	Somewhat Agree	
		6	Agree	
		7	Strongly Agree	
7	QUESTION	[...]	**I am able to communicate safely and effectively without disrupting patient sleep.**	
	VARIABLES	ICUP_03		
	VALUES	1	Strongly Disagree	
		2	Disagree	
		3	Somewhat Disagree	
		4	Neither Agree Nor Disagree	
		5	Somewhat Agree	
		6	Agree	
		7	Strongly Agree	
8	QUESTION	[...]	**[...]**	
	VARIABLES	ICUP_04	Noise levels in the ICU negatively impact on clinical care.	
		ICUP_05	Noise levels in the ICU negatively impact staff wellbeing.	
	VALUES	1	Strongly Disagree	
		2	Disagree	

			3	Somewhat Disagree
			4	Neither Agree Nor Disagree
			5	Somewhat Agree
			6	Agree
			7	Strongly Agree
9	QUESTION	[...]		**Impact of noise in the ICU is different to other areas such as Trauma and Emergency.**
	VARIABLES		ICUP_06	
	VALUES		1	Strongly Disagree
