# Supplementary material for: Mitigating Intensive Care Unit Noise: Design-Led Modeling Solutions, Calculated Acoustic Outcomes, and Cost Implications
Source: HERD. 2024 Mar 21;17(3):220–38. doi: 10.1177/19375867241237501 (PMC11457460; doi:10.1177/19375867241237501)
Supplement: Supplemental Material, sj-pdf-7-her-10.1177_19375867241237501 - Mitigating Intensive Care Unit Noise: Design-Led Modeling Solutions, Calculated Acoustic Outcomes, and Cost Implications [file sj-pdf-7-her-10.1177_19375867241237501.pdf]

Section 1. Questions about you

1. Male, Female, rather not say
2. How long have you been in the health profession?
3. In which ICU are you located (which hospital)?
4. How many years of ICU experience do you have?
    a. <5
    b. 6-9
    c. 10-19
    d. 20+

5. What is your designation or role?
    - RMO
    - Registrar
    - Consultant
    - EN
    - RN
    - CN
    - CNS/NUM
    - Allied health professional

Section 2. Multiple Choice

Rate the following statements on a scale of 1-7 where (1) = Strongly Disagree, (2) = Disagree, (3) = Somewhat Disagree, (4) = Neither Agree nor Disagree, (5) = Somewhat Agree, (6) Agree, (7) Strongly Agree:

6. The way in which the ICU is designed helps me to perform my duties
7. The ICU allows me to communicate efficiently with other staff in the ICU
8. I am able to communicate safely and effectively without disrupting patient sleep
9. Noise levels in the ICU negatively impact on clinical care
10. Noise levels in the ICU negatively impact staff well-being
11. Impact of noise in the ICU is different to other areas such as Trauma and Emergency

Rate the following statements on a scale of 1-7 where (1) = Strongly Disagree, (2) = Disagree, (3) = Somewhat Disagree, (4) = Neither Agree nor Disagree, (5) = Somewhat Agree, (6) Agree, (7) Strongly Agree:

12. Alarms
13. Talking between clinicians
14. Patient procedures
15. Managing ventilation and life support equipment
16. Visitors such as family members and/ or ancillary staff

Rate the following statements on a scale of 1-7 where (1) = Strongly Disagree, (2) = Disagree, (3) = Somewhat Disagree, (4) = Neither Agree nor Disagree, (5) = Somewhat Agree, (6) Agree, (7) Strongly Agree:

17. I believe a ‘quieter’ space provides clinicians with an increased ability to think

Rate the following statements on a scale of 1-7 where (1) = Strongly Disagree, (2) = Disagree, (3) = Somewhat Disagree, (4) = Neither Agree nor Disagree, (5) = Somewhat Agree, (6) Agree, (7) Strongly Agree:

18. I believe the quality of my work is negatively impacted by a noisy environment
19. I believe noise could contribute to medication errors
20. I believe noise decreases my sense of connection with patients
21. I believe noise decreases staff sense of satisfaction
22. I believe noise negatively impacts on patient sleep quality in the ICU

----End of Survey----
